# Supplementary material for: Dataset on a Benchmark for Equality Constrained Multi-objective Optimization
Source: Data Brief. 2020 Jan 11;29:105130. doi: 10.1016/j.dib.2020.105130 (PMC6994572; doi:10.1016/j.dib.2020.105130)
Supplement: Multimedia component 1 [file mmc1.zip › DataInBrief/datainbrief.docx]

**Title:** *Dataset on a Benchmark for Equality Constrained Multi-objective Optimization*

**Authors:** Oliver Cuate^1^, Lourdes Uribe^2^, Adriana Lara^2^, Oliver Schütze^1,3^

**Affiliations:**

1. Department of Computer Science, CINVESTAV-IPN, Mexico City, Mexico.
2. ESFM, Instituto Politécnico Nacional, Mexico City, Mexico.
3. Dr. Rodolfo Quintero Chair, UAM Cuajimalpa, Mexico City, Mexico.

**Corresponding author(s):** Oliver Cuate (ocuate@computacion.cs.cinvestav.mx)

**Abstract**

In this Data in Brief, we provide the source code for the equality constrained multi-objective optimization benchmark problems EqDTLZ 1-4 and EqIDTLZ 1-2 proposed in the research article “*A Benchmark for Equality Constrained Multi-objective Optimization”* [1]. Further, we provide the codes for the multi-objective evolutionary algorithms NSGA-II, NSGA-III, aNSGA-III, GDE3, MOEA/D/D and PPS and their numerical approximations on the above mentioned test functions. All codes are provided in Matlab using the PlatEMO classes version 2.0 in order to test different algorithms.

**Keywords**: Evolutionary computation, multi-objective optimization, equality constraints, benchmarking

**Specifications Table**

| **Subject** | Control and Optimization |
| --- | --- |
| **Specific subject area** | Constrained Multi-objective Optimization |
| **Type of data** | Table  Figure  Text file  Matlab file |
| **How data were acquired** | Matlab program, PlatEMO platform |
| **Data format** | Raw  Analysed |
| **Parameters for data collection** | The matlab files provide the proposed test functions, for this case D is the number of decision variables, M is the number of objectives, N is the population size, c and r are the centre and radius of each constraint.  Figures show the approximation of the Pareto set/front computed by the selected MOEAs for different number of: objectives (M = 3 or M =4), constraints (p = 1 and p =2) and function evaluations ( 150 000 and 500 000)  Table present the performance indicator value and the feasibility radio for a specific test function (Eq-DTLZ4) for |
| **Description of data collection** | For the figures present the approximation of the solution sets over 30 independent runs for each MOEA. The matlab files include the PlatEMO 2.0 code of each test function of the proposed benchmark. |
| **Data accessibility** | The data are in this article |
| **Related research article** | It is a direct submission to Data in Brief, the most relevant research is in [1]. |

**Value of the Data**

- This dataset shows the obtained Pareto sets and fronts by different state-of-the-art MOEAs for the proposed benchmark for different budgets of function calls
- This dataset includes the coded test functions in order to run over PlatEMO platform.
- The dataset provides empirical evidence that the proposed benchmark is a good candidate for testing EMO algorithms.
- The dataset reveals that the proposed problems are indeed hard to solve and further investigation has to be done for the reliable treatment of equality constrained MOPs.

**Data**

The dataset contains the obtained solution sets of the proposed equality constrained MOPs for the following state-of-the-art MOEAs: ANSGA-III, GDE3, MOEAD/D/D, NSGA-II, NSGA-III and PPS.

Figure 1 presents the obtained approximation of ANSGA-III on Eq-DTLZ1 and a budget of 150,000 function evaluations. (a) and (b) shows the one with best $\Delta_{2}$ value. (c) and (d) shows the one with best HV value. Although many solutions are feasible, the distributions are not satisfactory.


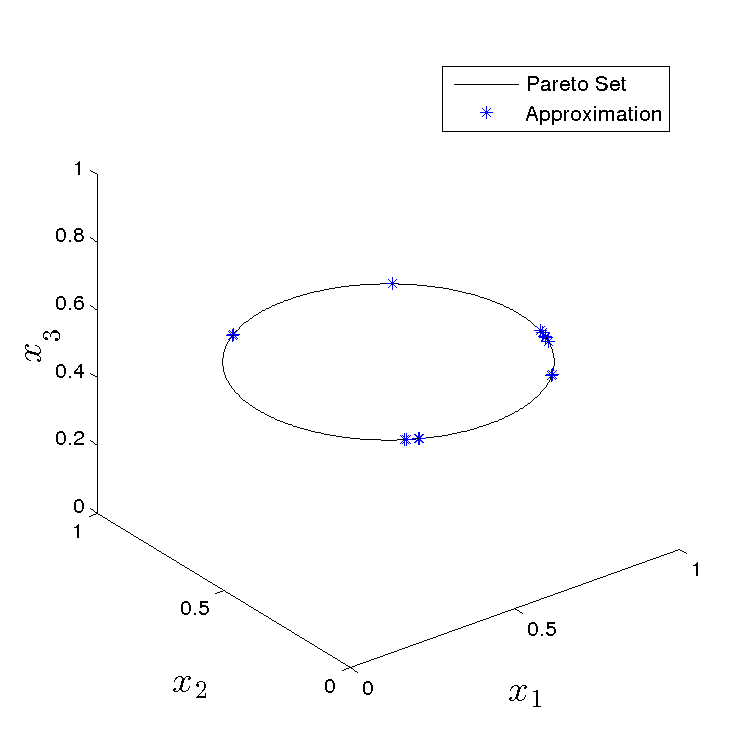

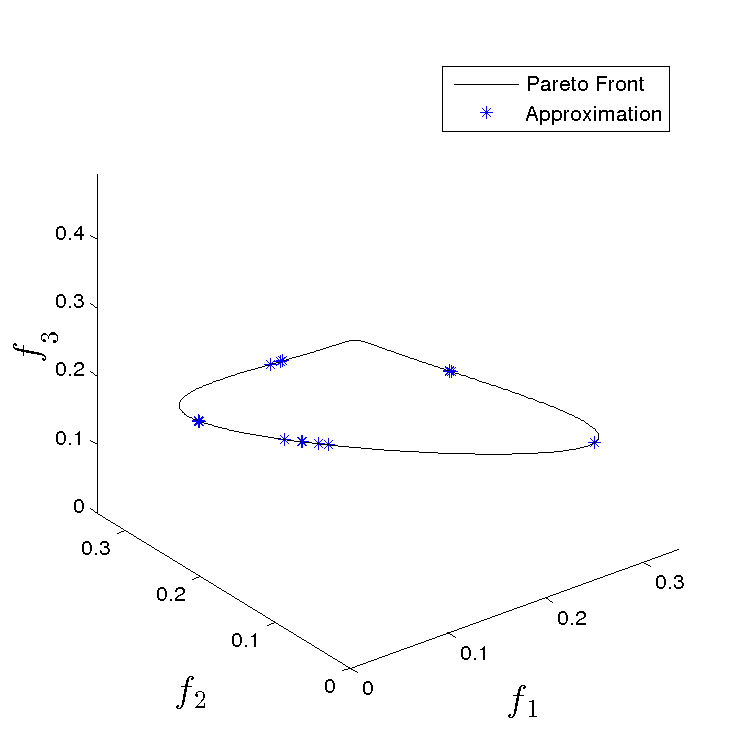


1. Pareto Set (b) Pareto Front


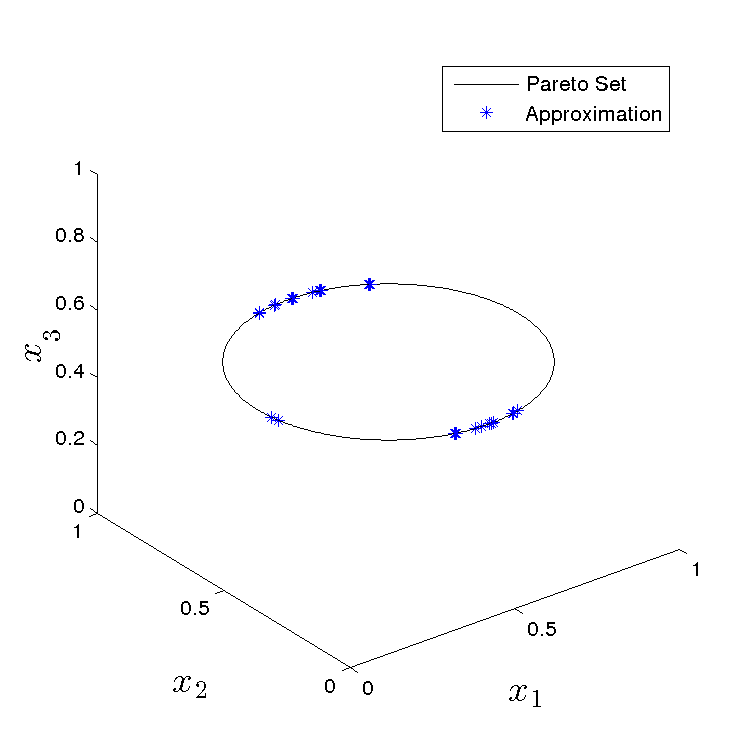

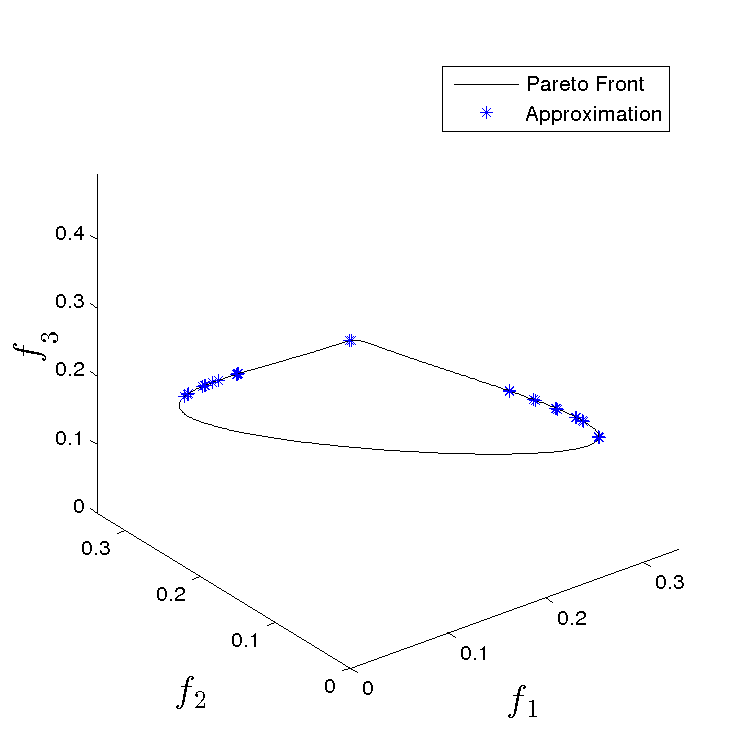


(c) Pareto Set (d) Pareto Front

Figure 1 ANSGA-III approximations on Eq-DTLZ1 and a budget of 150,000 functions evaluations.

Figure 2 presents the obtained approximation of GDE3 on Eq-DTLZ1 and a budget of 150,000 function evaluations. (a) and (b) shows the one with best $\Delta_{2}$ value. (c) and (d) shows the one with best HV value. Although many solutions are feasible, most of them are far from the Pareto set/front.


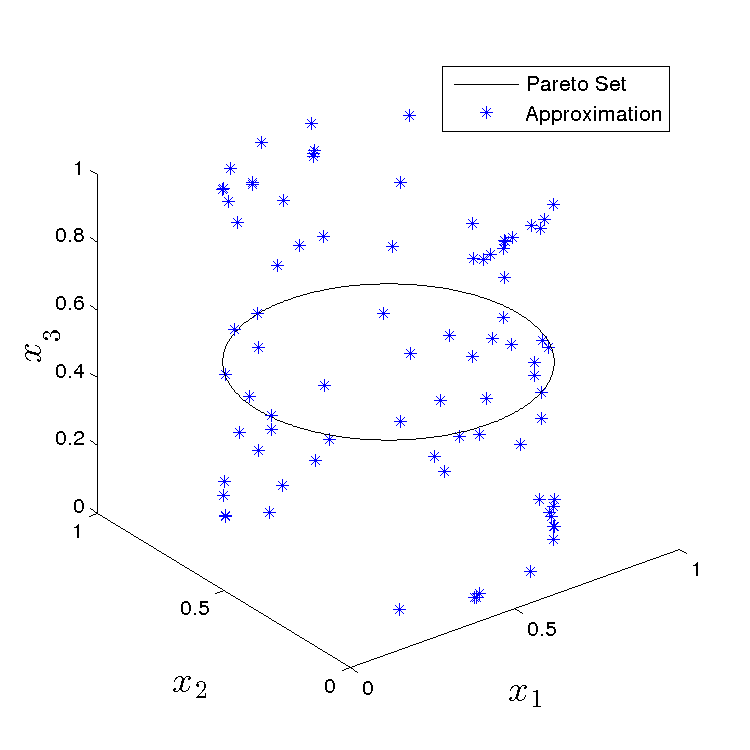

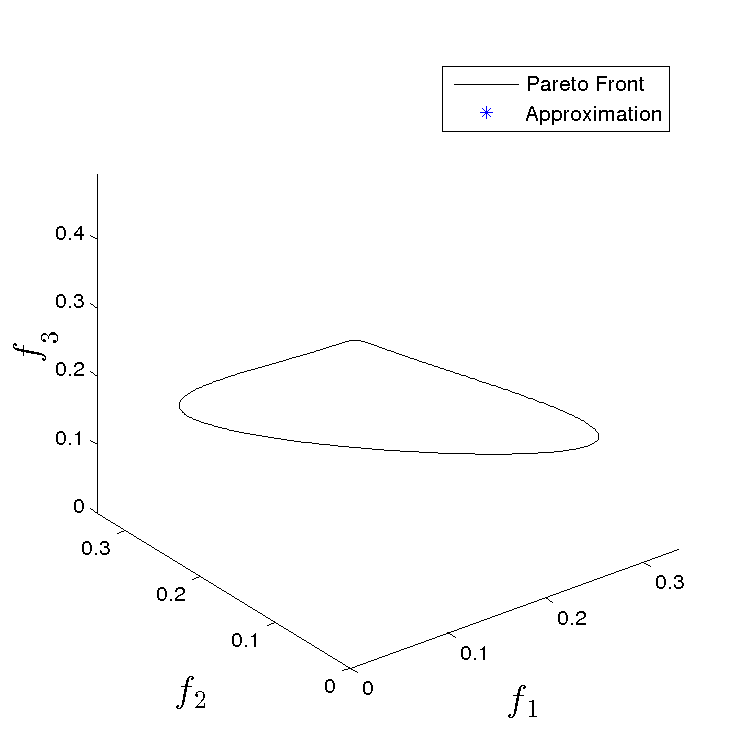


1. Pareto Set (b) Pareto Front


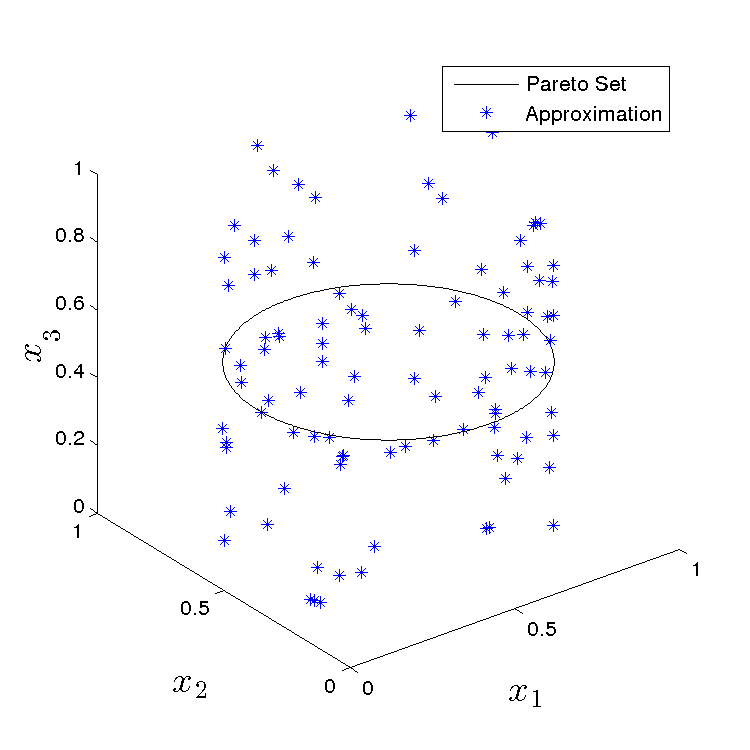

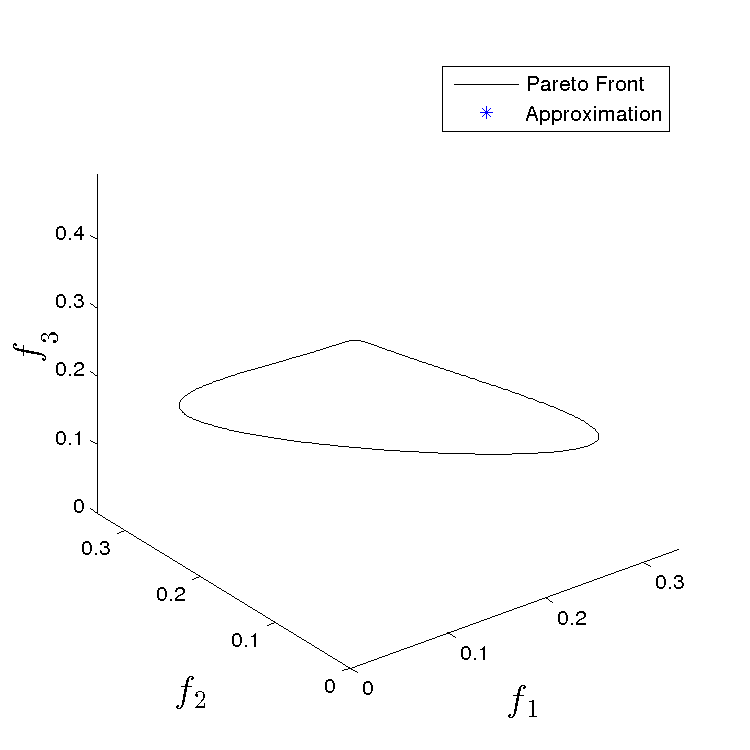


(c) Pareto Set (d) Pareto Front

Figure 2 GDE3 approximations on Eq-DTLZ1 and a budget of 150,000 functions evaluations.

Figure 3 presents the obtained approximation of MOEA/D/D on Eq-DTLZ1 and a budget of 150,000 function evaluations. (a) and (b) shows the one with best $\Delta_{2}$ value. (c) and (d) shows the one with best HV value. Though we observe a better variation of the solution, the overall distribution is still poor.


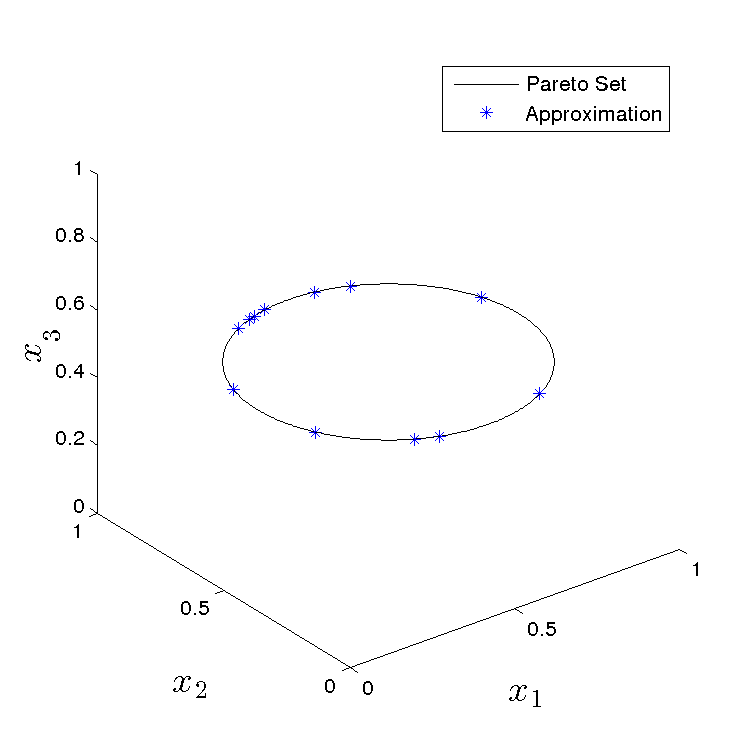

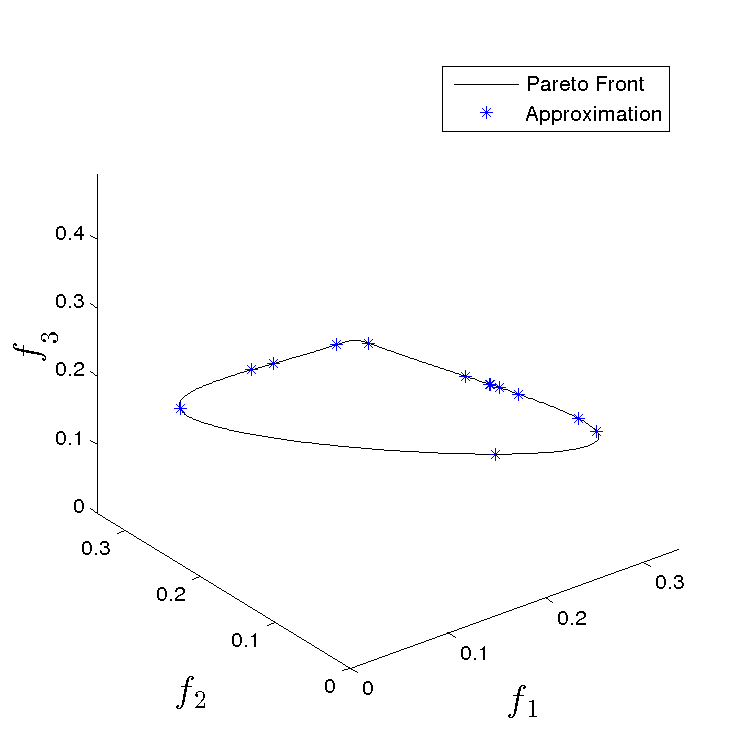


1. Pareto Set (b) Pareto Front


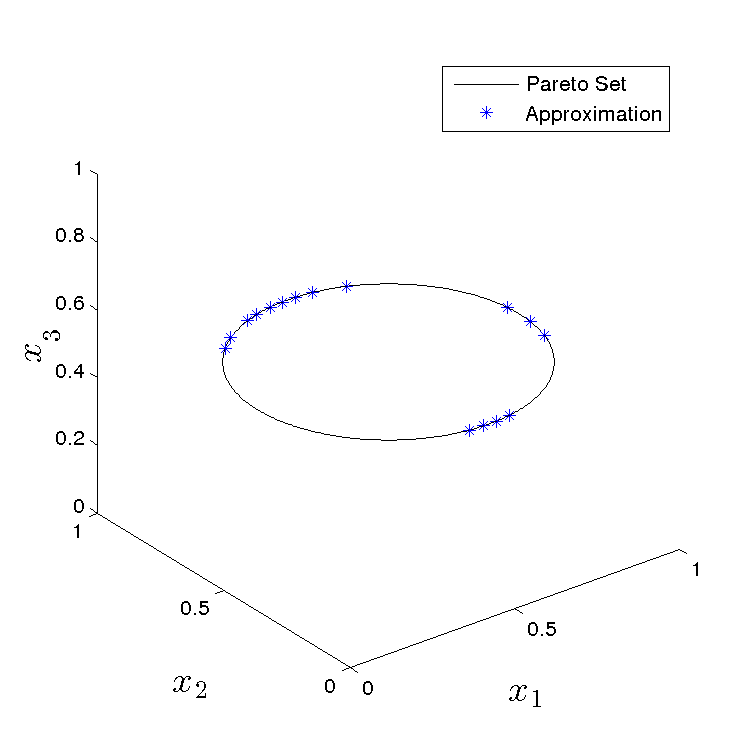

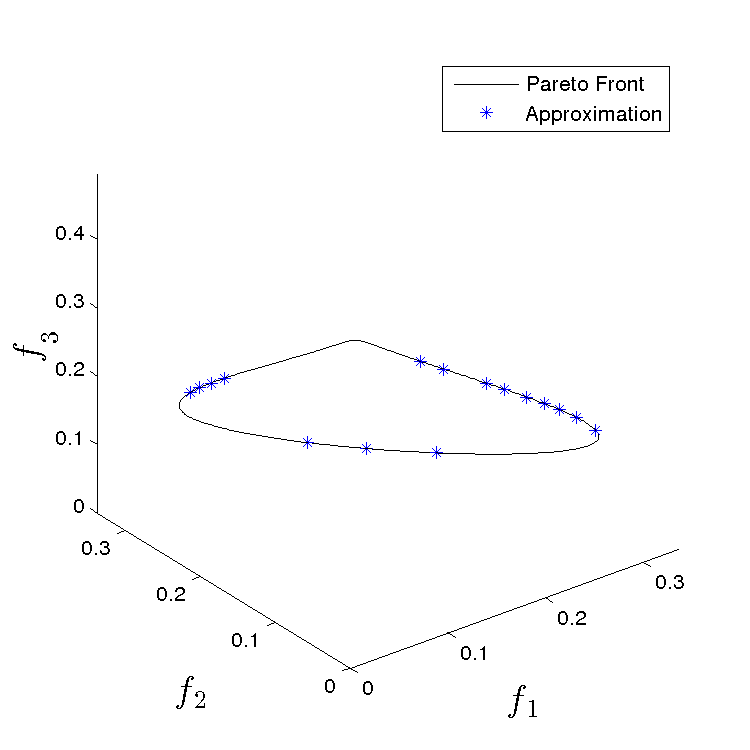


(c) Pareto Set (d) Pareto Front

Figure 3 MOEA/D/D approximations on Eq-DTLZ1 and a budget of 150,000 functions evaluations.

Figure 4 presents the obtained approximation of NSGA-II on Eq-DTLZ1 and a budget of 150,000 function evaluations. (a) and (b) shows the one with best $\Delta_{2}$ value. (c) and (d) shows the one with best HV value. This MOEA obtained the best approximation, however, still not satisfying.


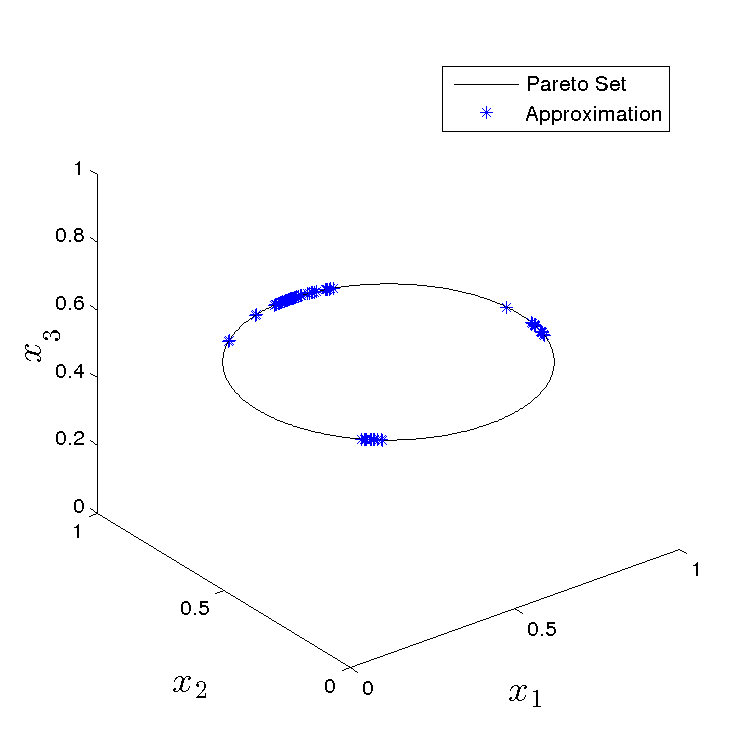

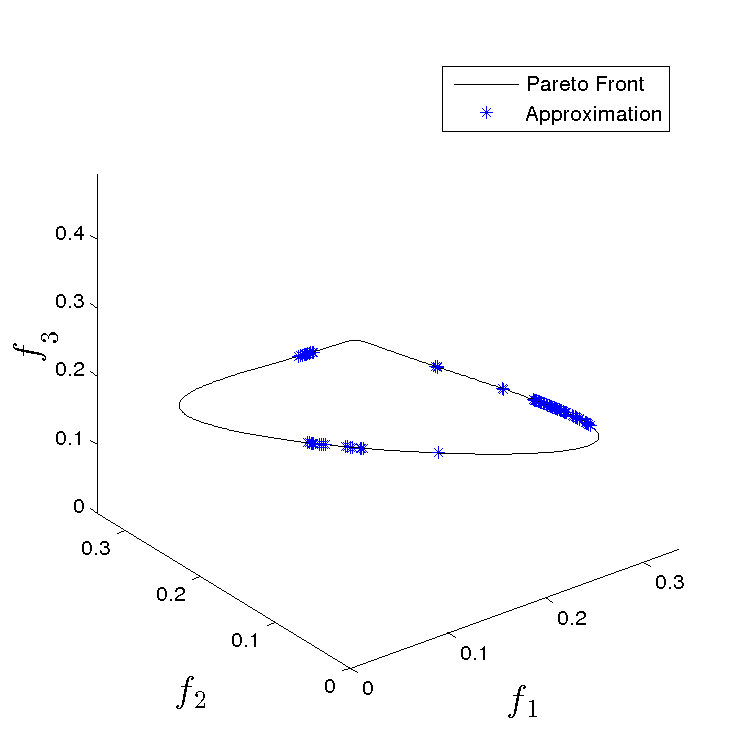


1. Pareto Set (b) Pareto Front


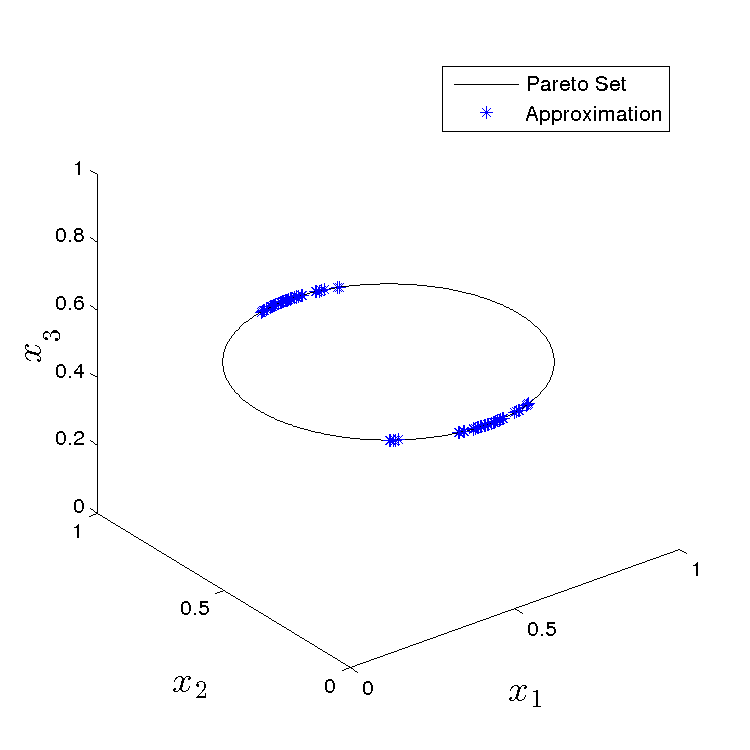

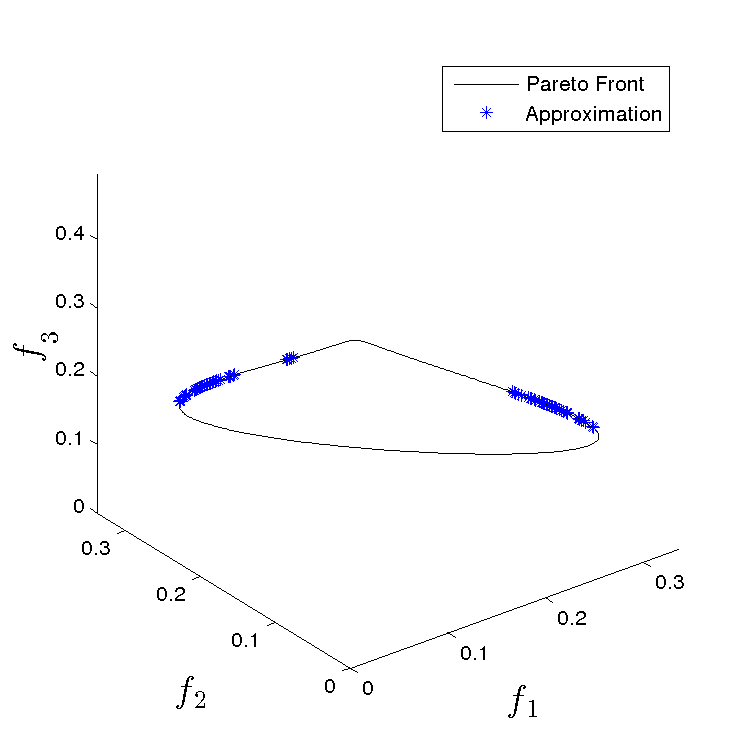


(c) Pareto Set (d) Pareto Front

Figure 4 NSGA-II approximations on Eq-DTLZ1 and a budget of 150,000 functions evaluations.

Figure 5 presents the obtained approximation of NSGA-III on Eq-DTLZ1 and a budget of 150,000 function evaluations. (a) and (b) shows the one with best $\Delta_{2}$ value. (c) and (d) shows the one with best HV value.


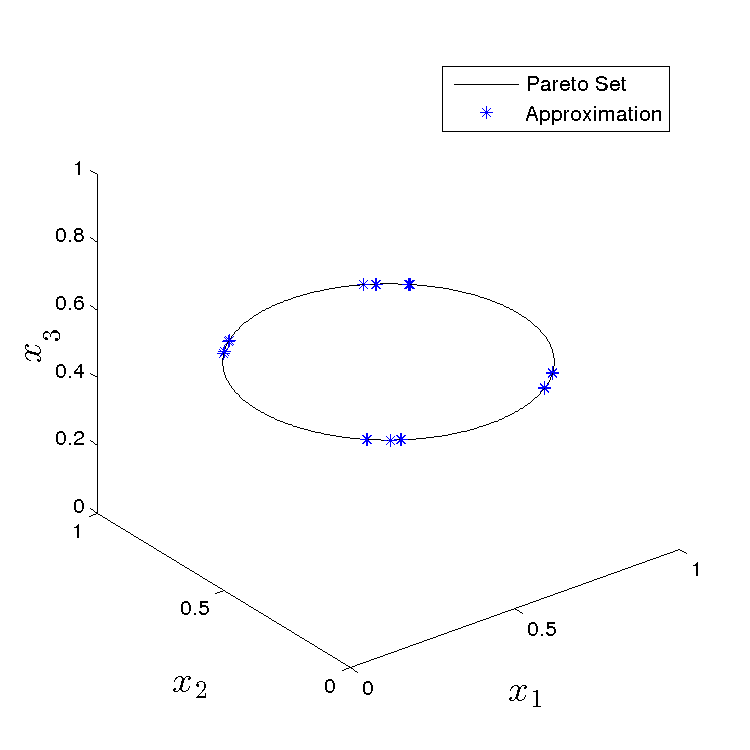

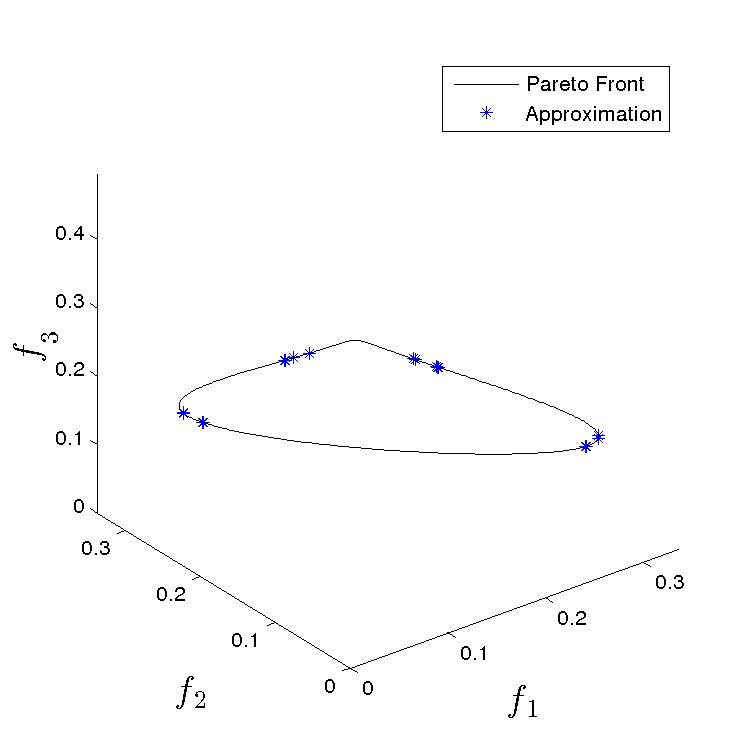


1. Pareto Set (b) Pareto Front


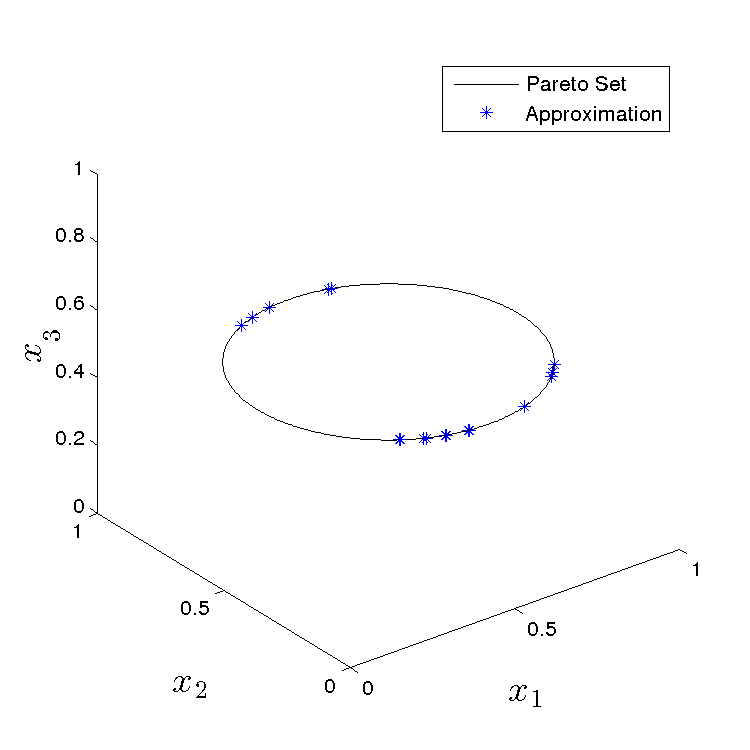

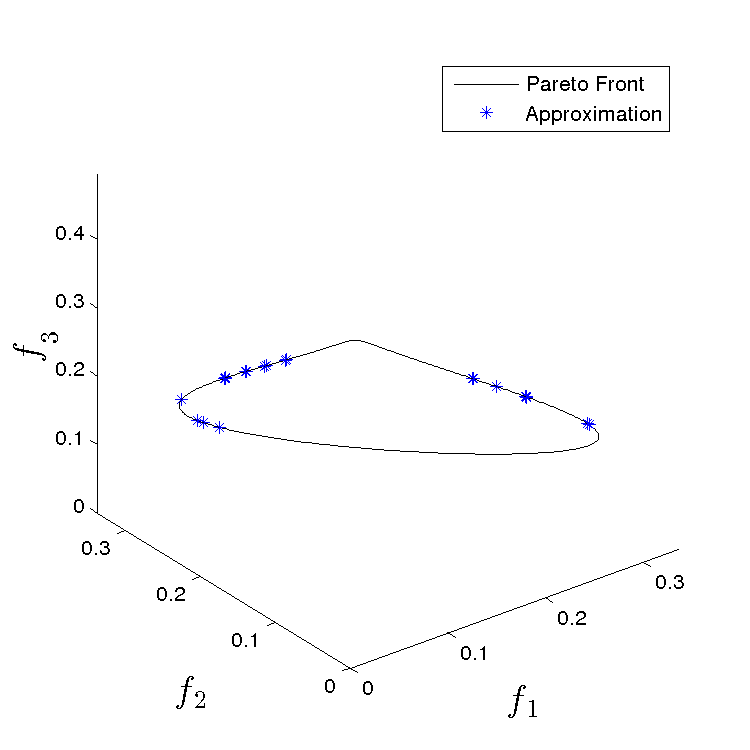


(c) Pareto Set (d) Pareto Front

Figure 5 NSGA-III approximations on Eq-DTLZ1 and a budget of 150,000 functions evaluations.

Figure 6 presents the obtained approximation of PPS on Eq-DTLZ1 and a budget of 150,000 function evaluations. (a) and (b) shows the one with best $\Delta_{2}$ value. (c) and (d) shows the one with best HV value.


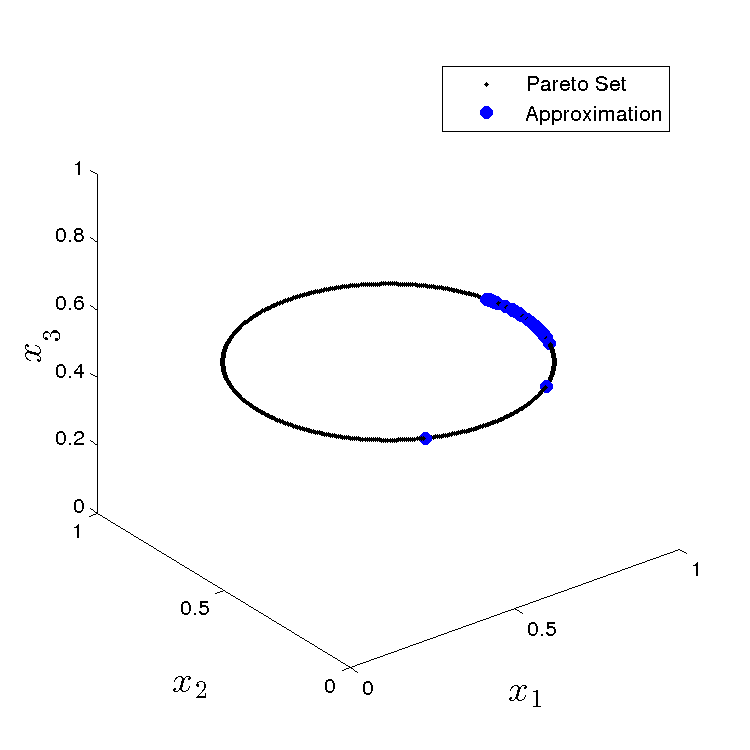

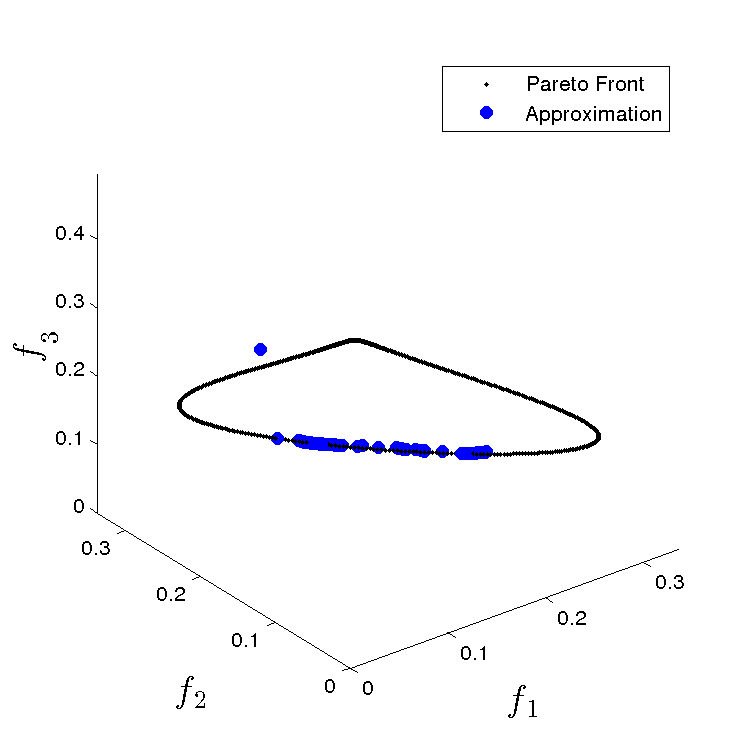


1. Pareto Set (b) Pareto Front


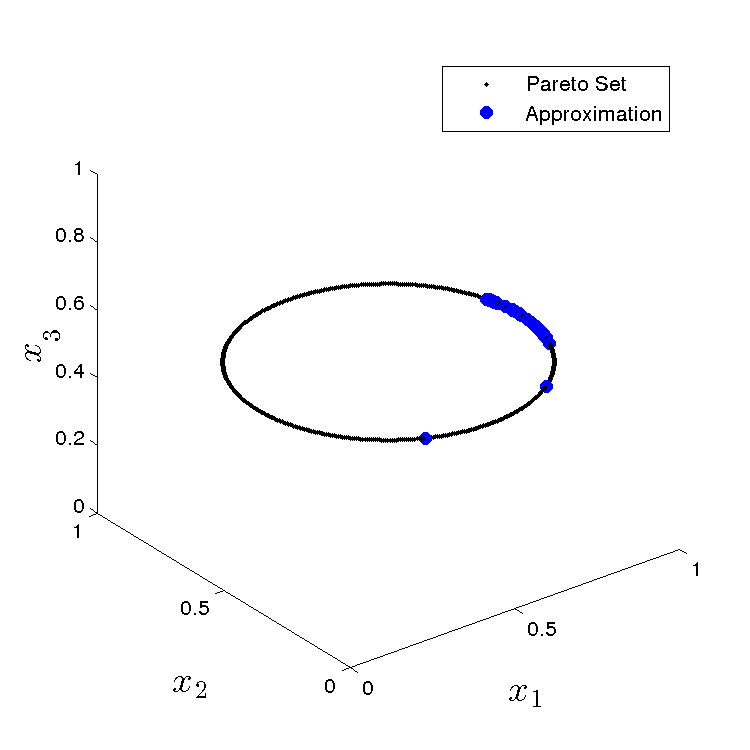

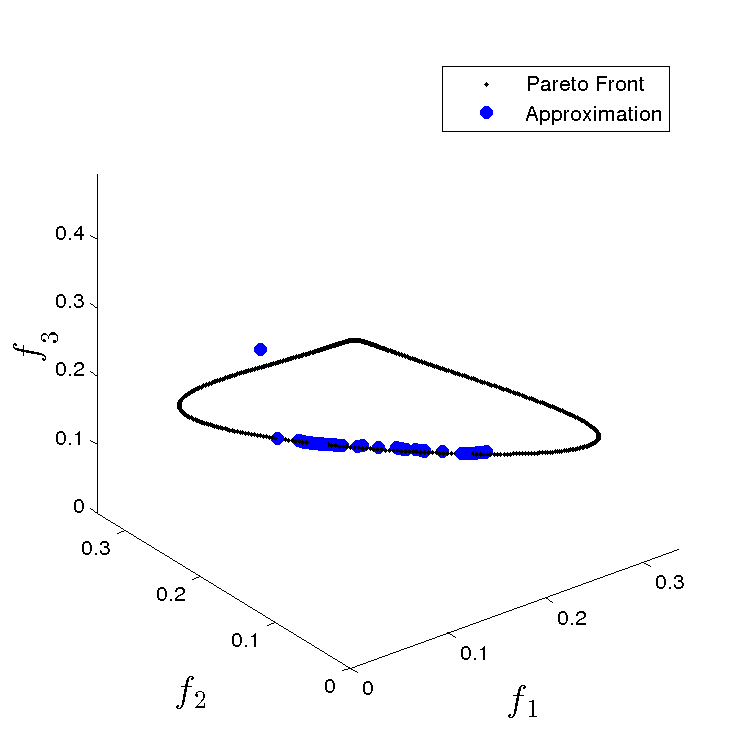


(c) Pareto Set (d) Pareto Front

Figure 6 PPS approximations on Eq-DTLZ1 and a budget of 150,000 functions evaluations.

Figure 7 presents the obtained approximation of ANSGA-III on Eq-DTLZ2 and a budget of 500,000 function evaluations. (a) and (b) shows the one with best $\Delta_{2}$ value. (c) and (d) shows the one with best HV value.


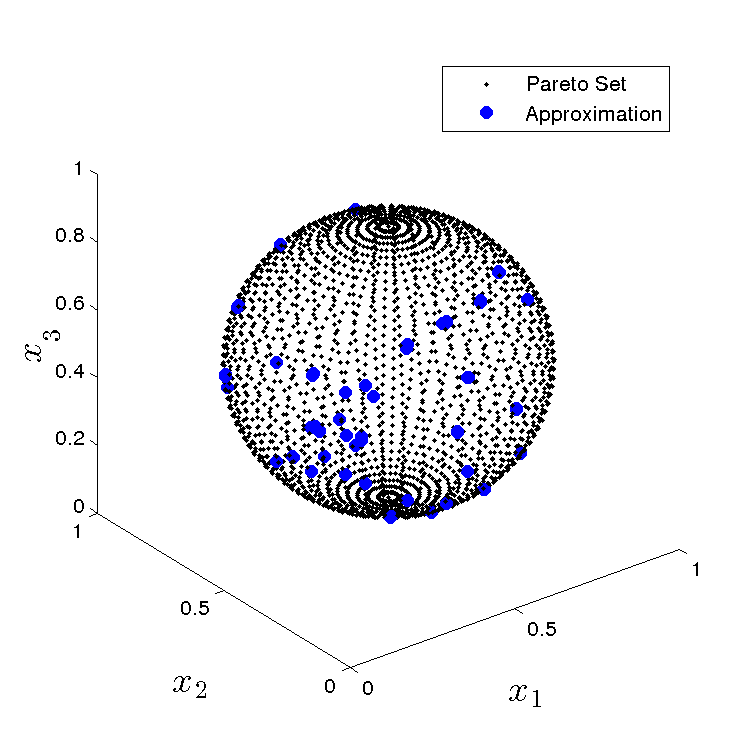

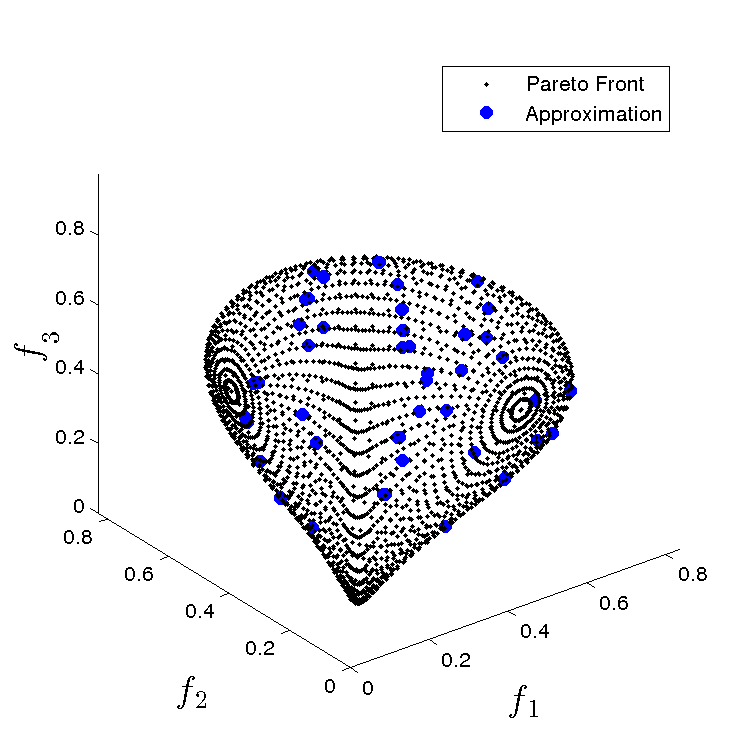


1. Pareto Set (b) Pareto Front


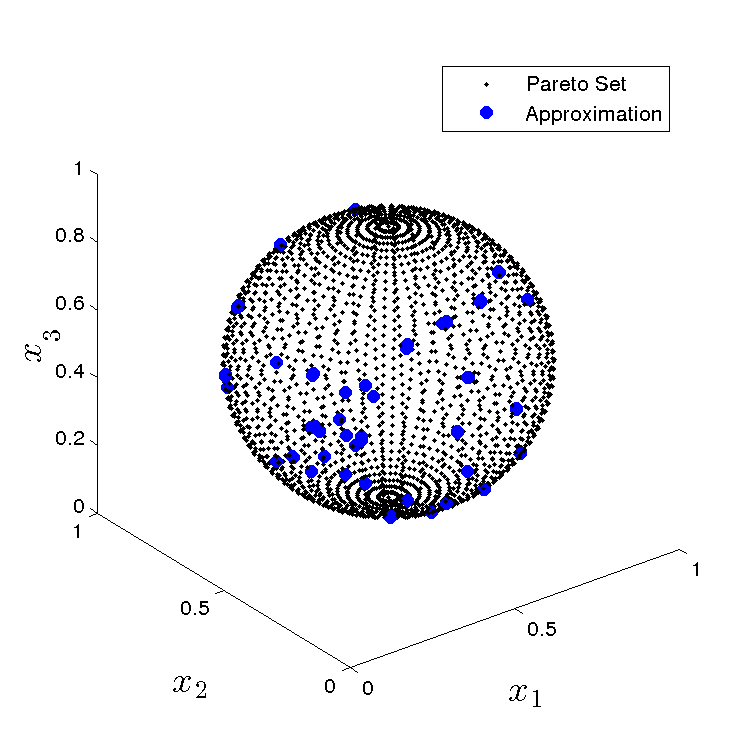

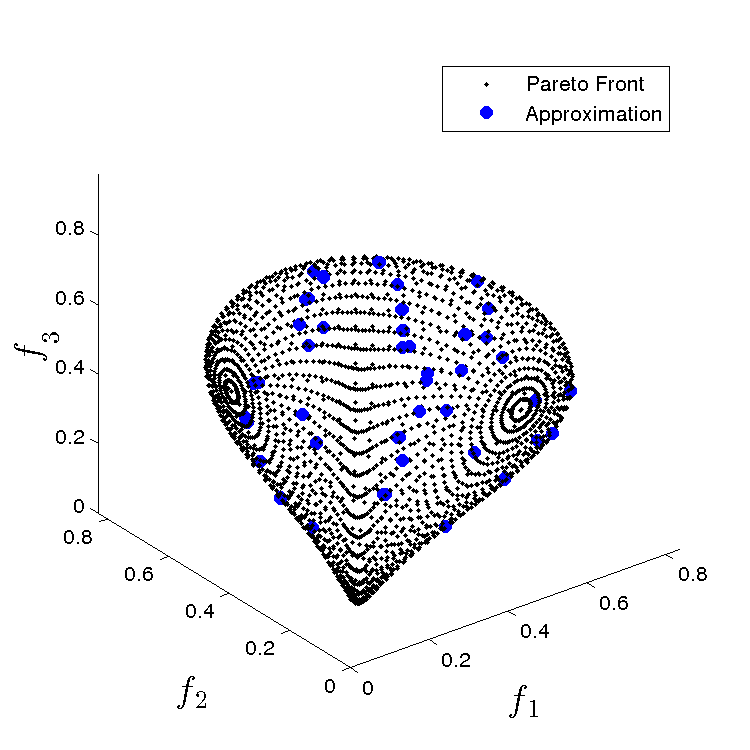


(c) Pareto Set (d) Pareto Front

Figure 7 ANSGA-III approximations on Eq-DTLZ2 and a budget of 500,000 functions evaluations.

Figure 8 presents the obtained approximation of GDE3 on Eq-DTLZ2 and a budget of 500,000 function evaluations. (a) and (b) shows the one with best $\Delta_{2}$ value. (c) and (d) shows the one with best HV value. The plots in decision space are three dimensional projections. Though most solutions appear to be on the Pareto set, they are in fact far away as can be seen in the respective plots in the image space.


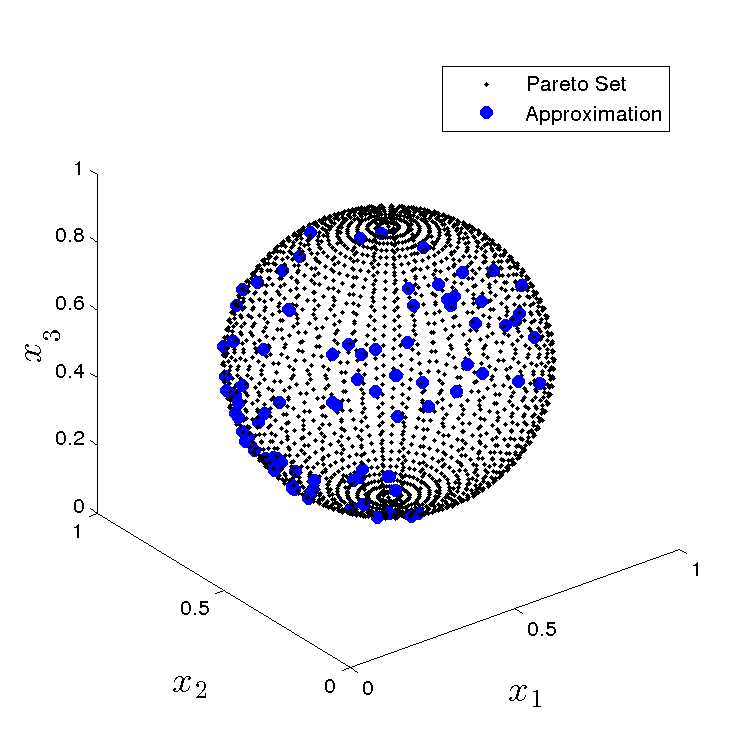

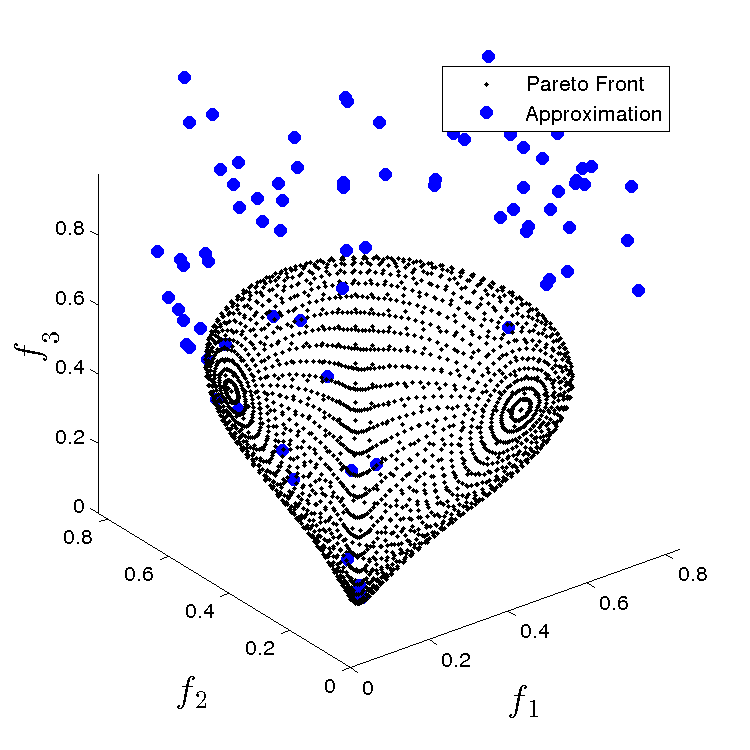


1. Pareto Set (b) Pareto Front


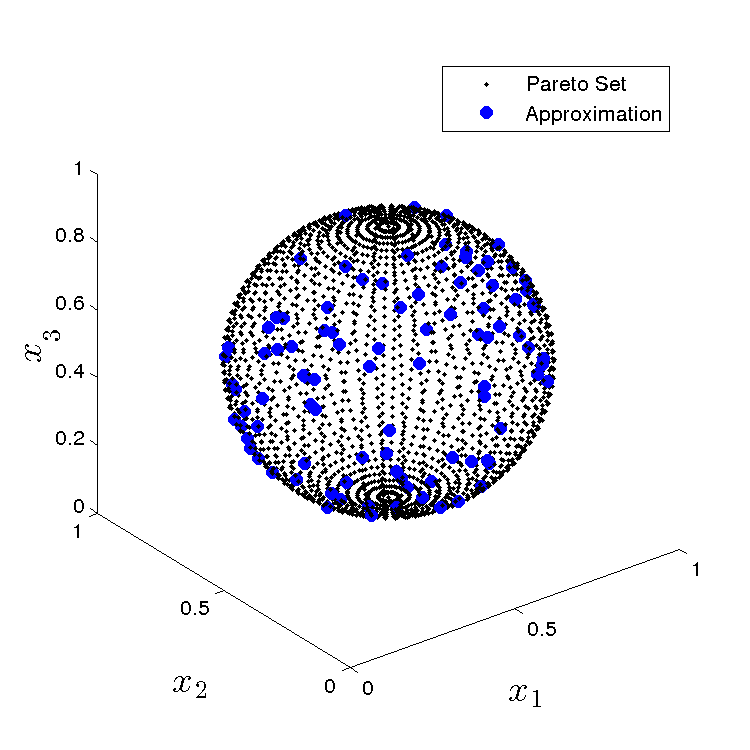

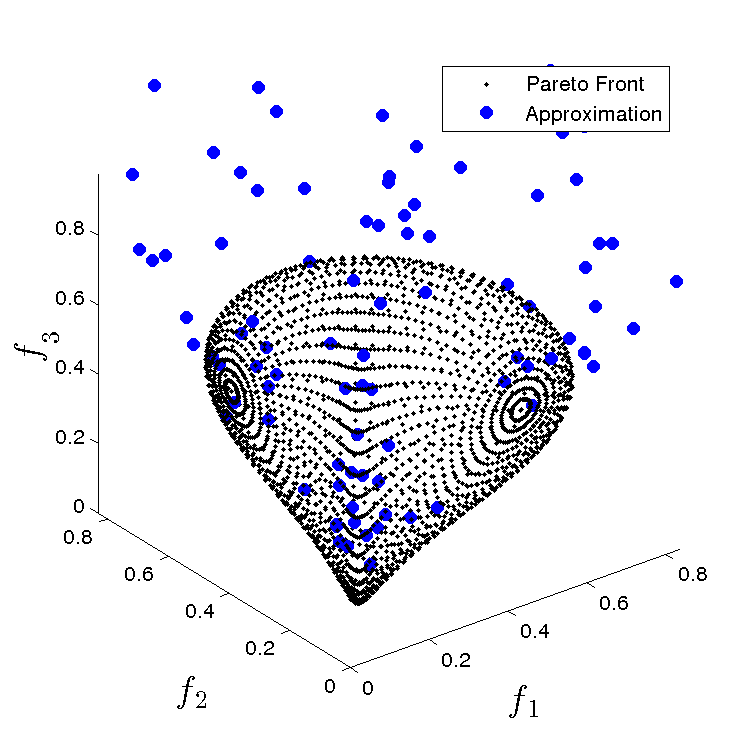


(c) Pareto Set (d) Pareto Front

Figure 8 GDE3 approximations on Eq-DTLZ2 and a budget of 500,000 functions evaluations.

Figure 9 presents the obtained approximation of MOEA/D/D on Eq-DTLZ2 and a budget of 500,000 function evaluations. (a) and (b) shows the one with best $\Delta_{2}$ value. (c) and (d) shows the one with best HV value. This method is capable of finding some Pareto optimal solutions, but the distribution is still poor.


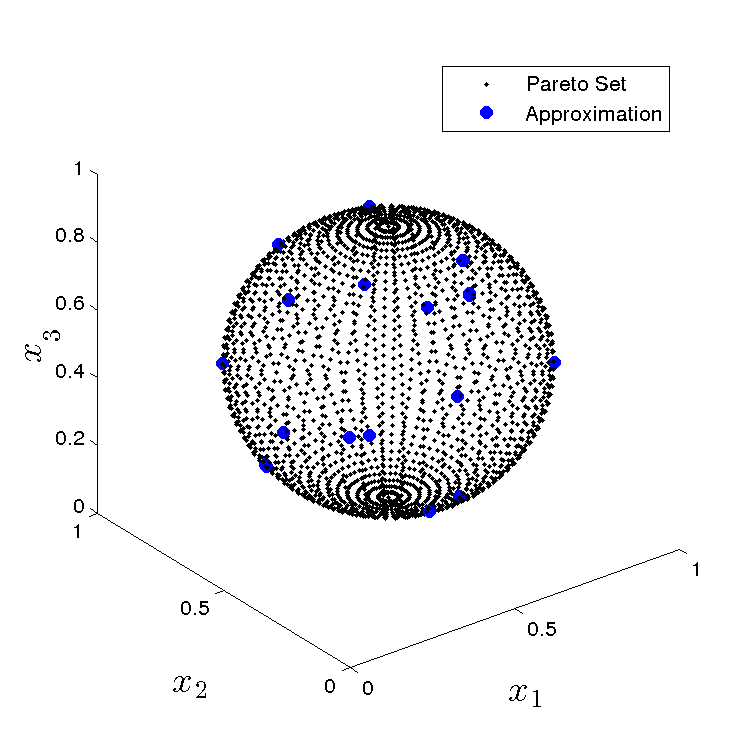

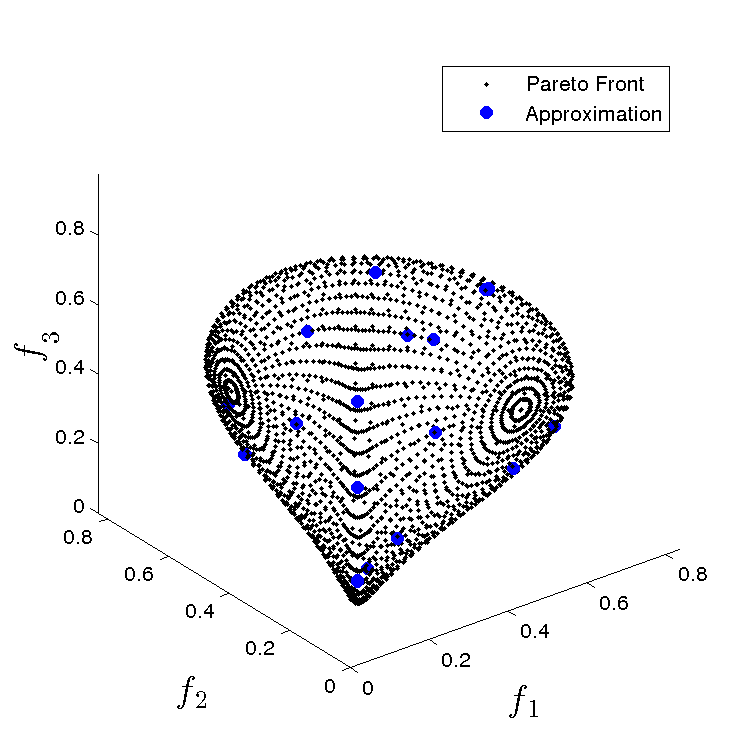


1. Pareto Set (b) Pareto Front


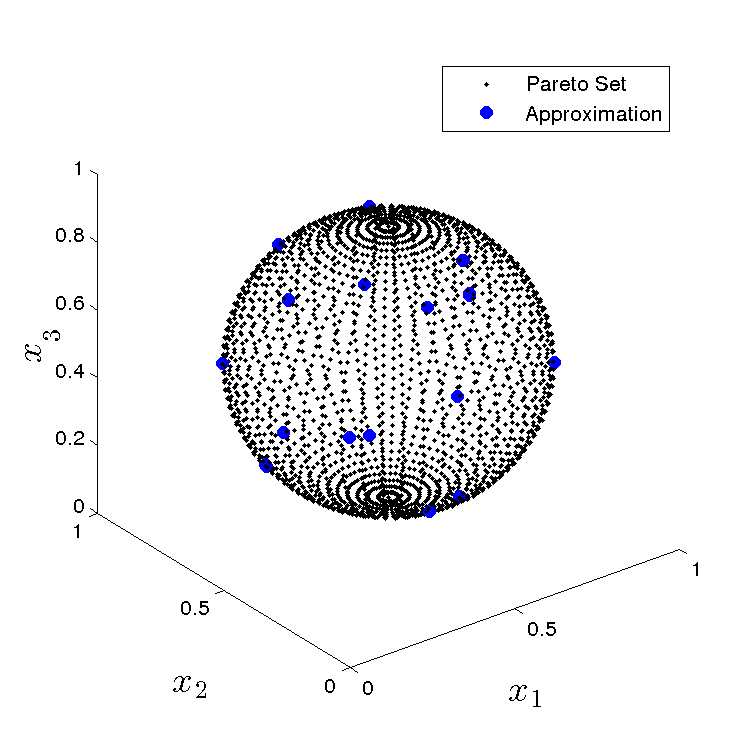

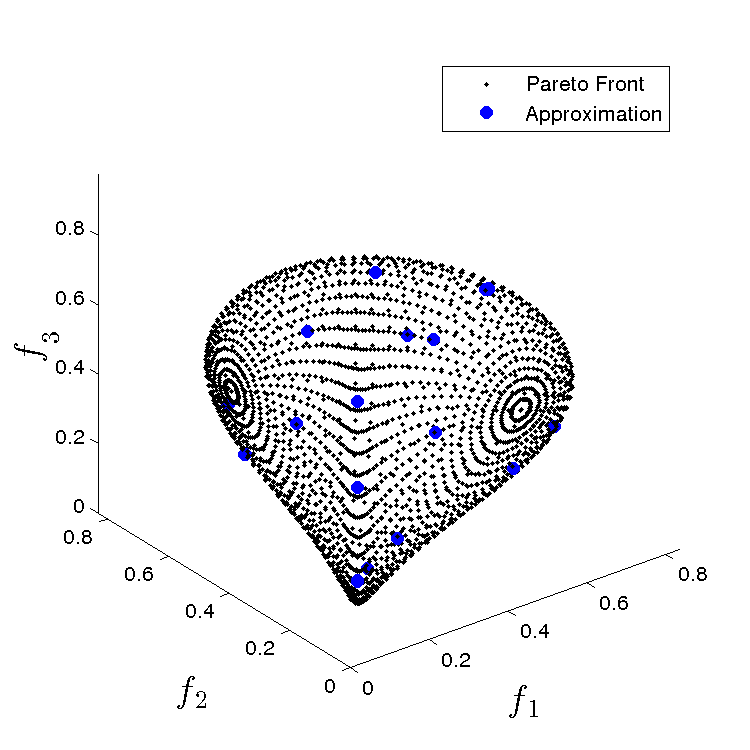


(c) Pareto Set (d) Pareto Front

Figure 9 MOEA/D/D approximations on Eq-DTLZ2 and a budget of 500,000 functions evaluations.

Figure 10 presents the obtained approximation of NSGA-II on Eq-DTLZ2 and a budget of 500,000 function evaluations. (a) and (b) shows the one with best $\Delta_{2}$ value. (c) and (d) shows the one with best HV value. This MOEA obtained the best approximation, however, still not satisfying.


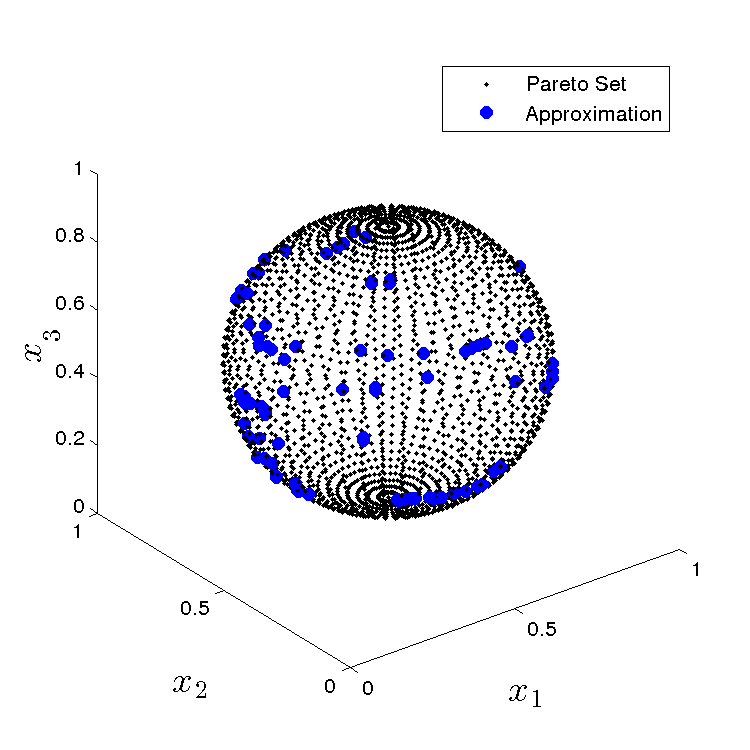

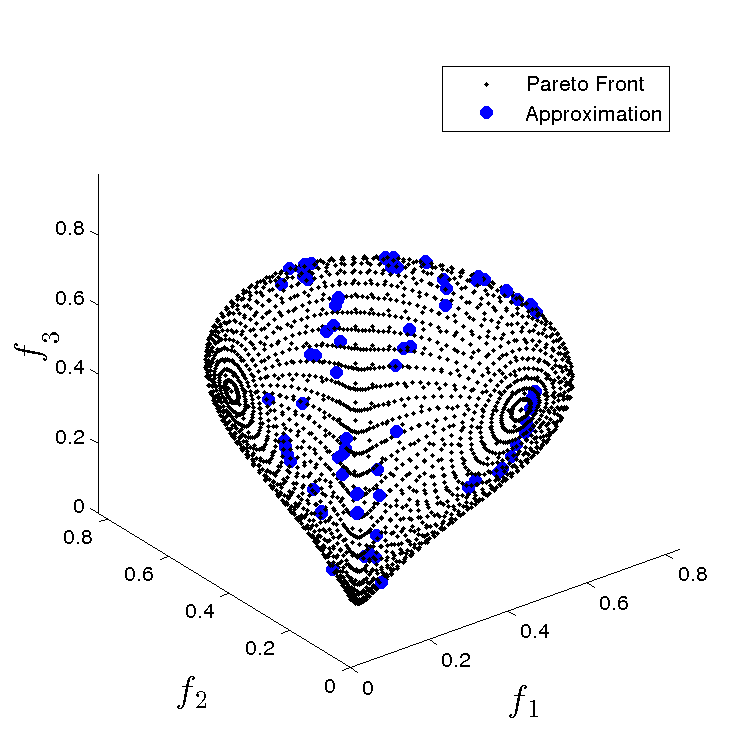


1. Pareto Set (b) Pareto Front


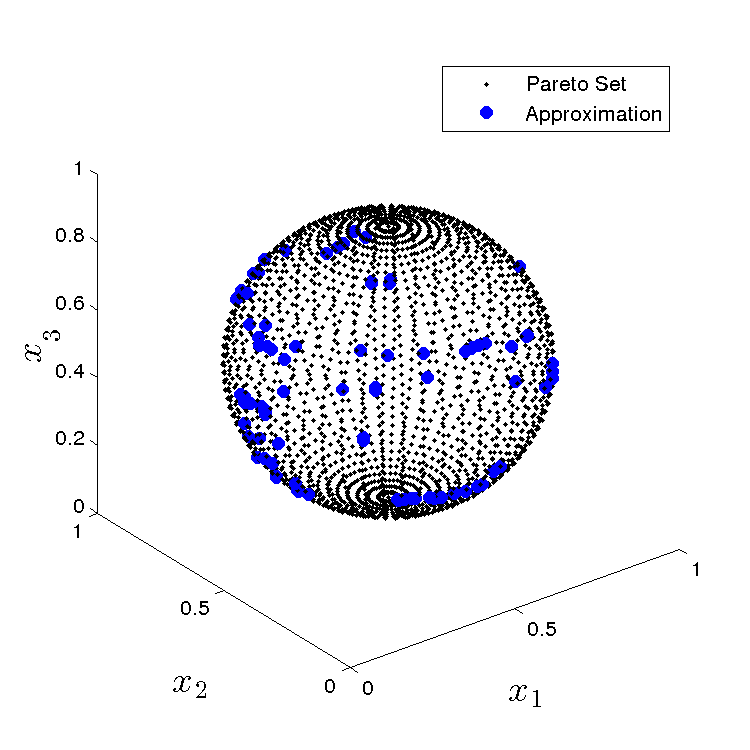

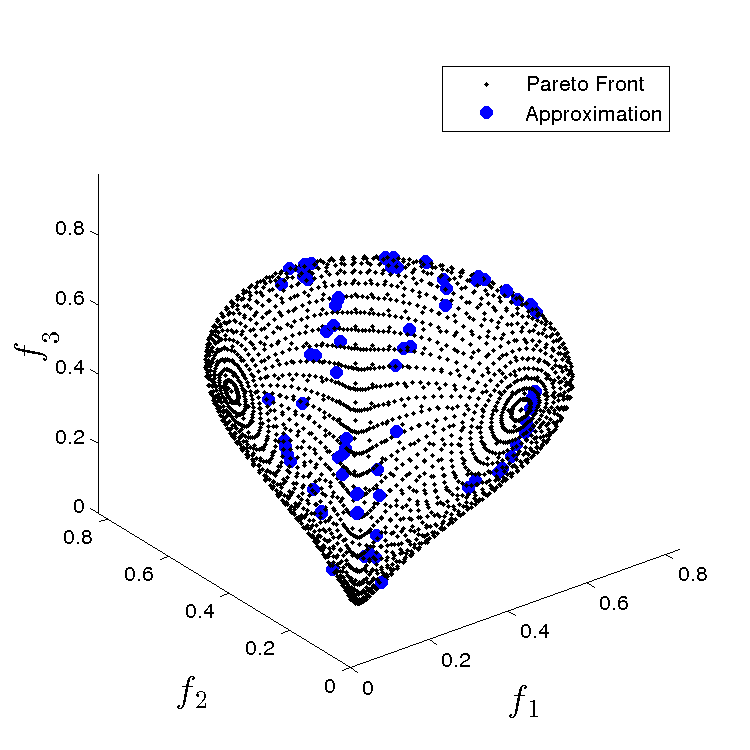


(c) Pareto Set (d) Pareto Front

Figure 10 NSGA-II approximations on Eq-DTLZ2 and a budget of 500,000 functions evaluations.

Figure 11 presents the obtained approximation of NSGA-III on Eq-DTLZ2 and a budget of 500,000 function evaluations. (a) and (b) shows the one with best $\Delta_{2}$ value. (c) and (d) shows the one with best HV value.


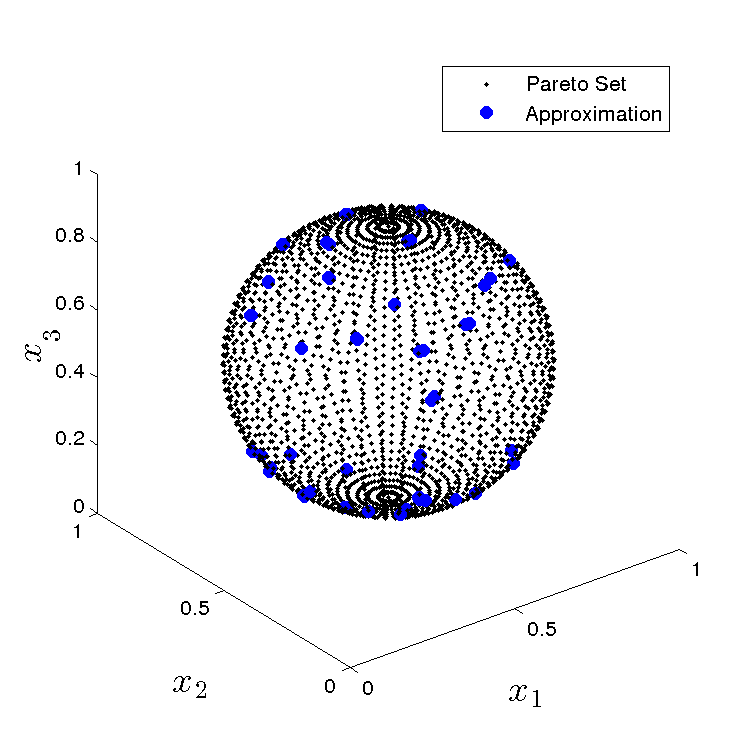

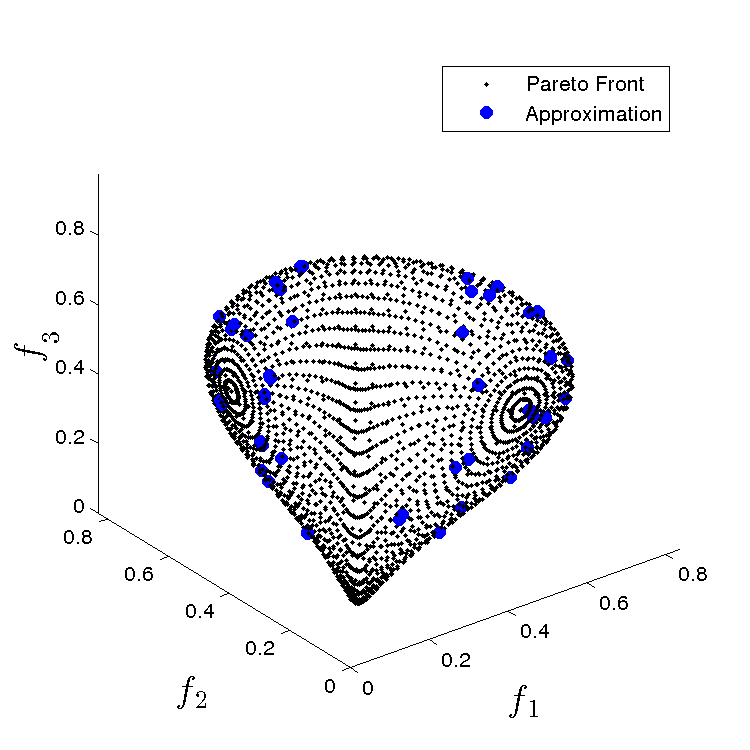


1. Pareto Set (b) Pareto Front


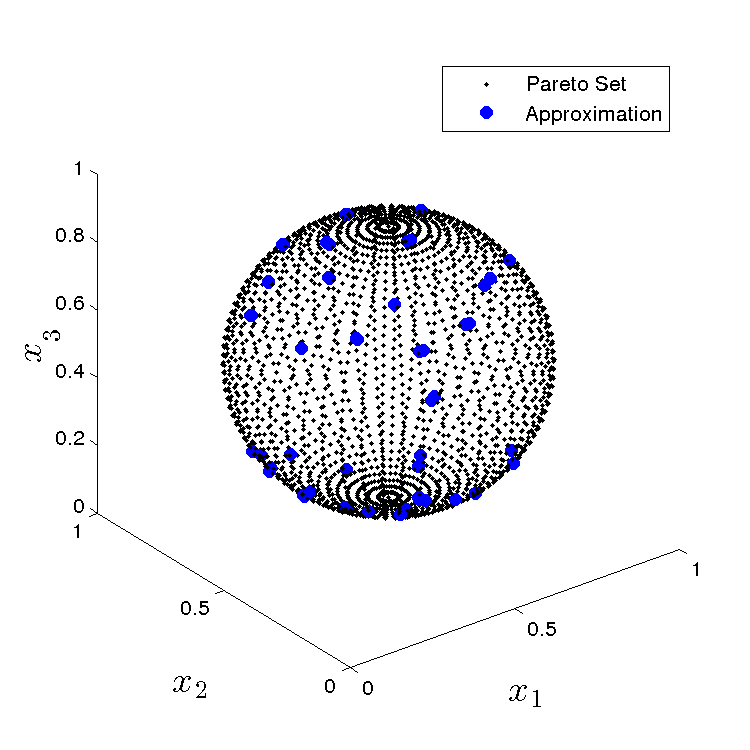

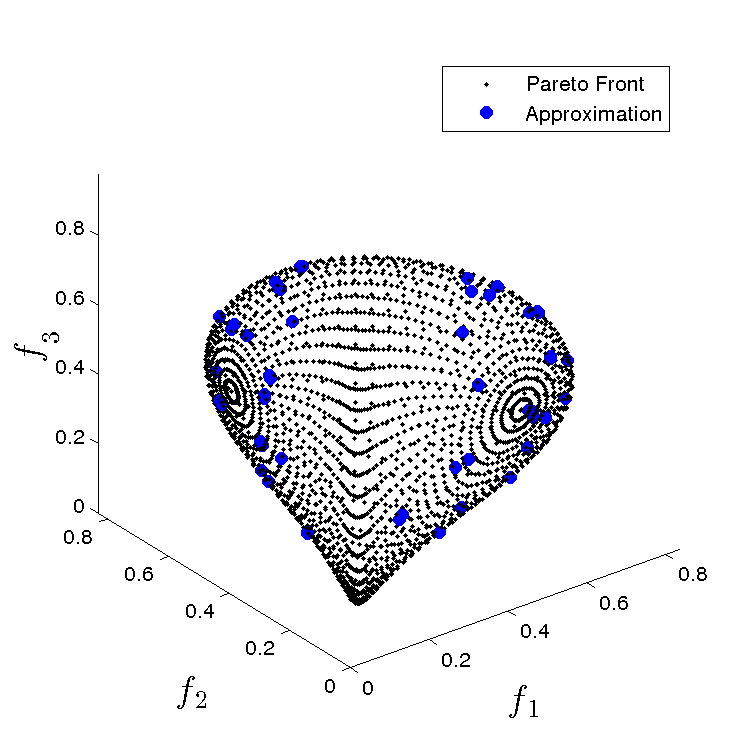


(c) Pareto Set (d) Pareto Front

Figure 11 NSGA-III approximations on Eq-DTLZ2 and a budget of 500,000 functions evaluations.

Figure 12 presents the obtained approximation of PPS on Eq-DTLZ2 and a budget of 500,000 function evaluations. (a) and (b) shows the one with best $\Delta_{2}$ value. (c) and (d) shows the one with best HV value.


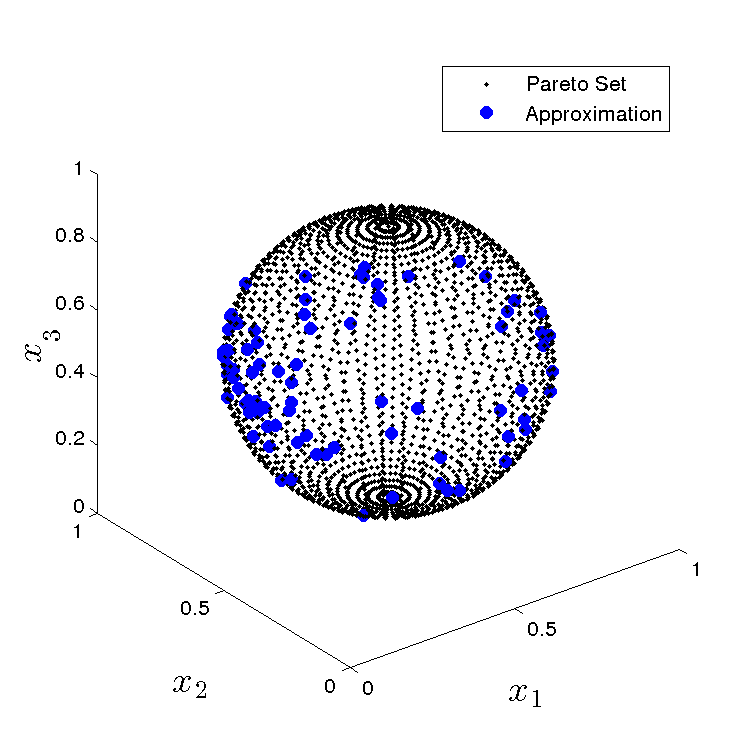

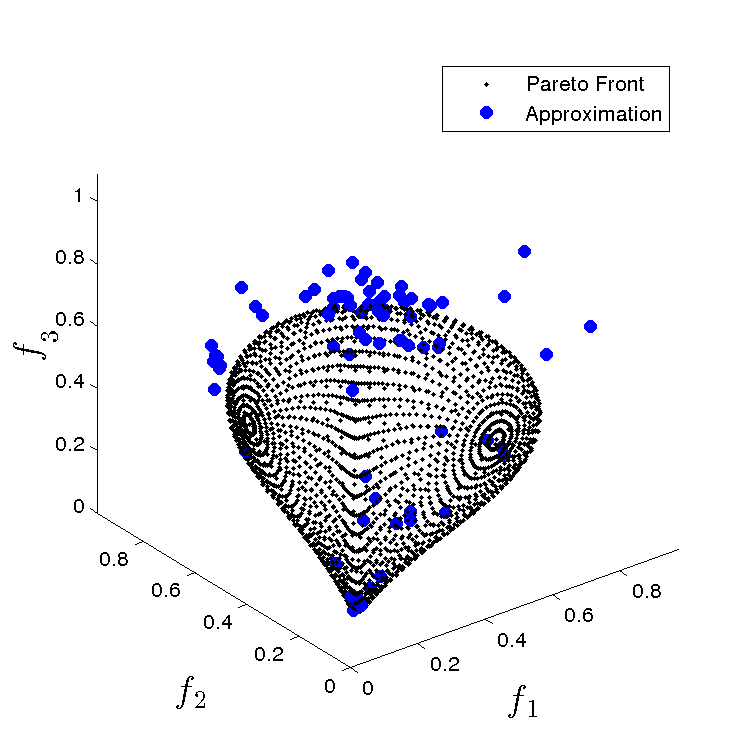


1. Pareto Set (b) Pareto Front


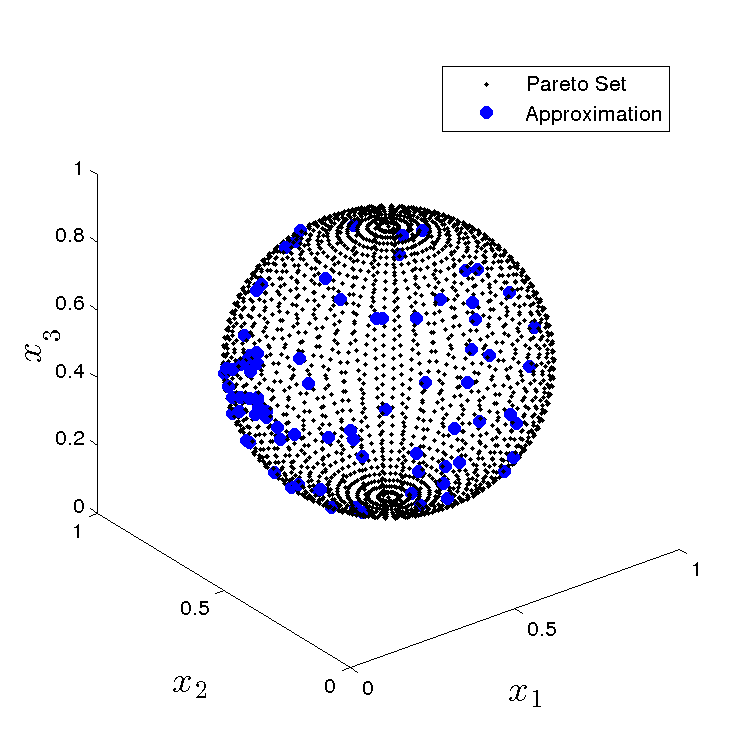

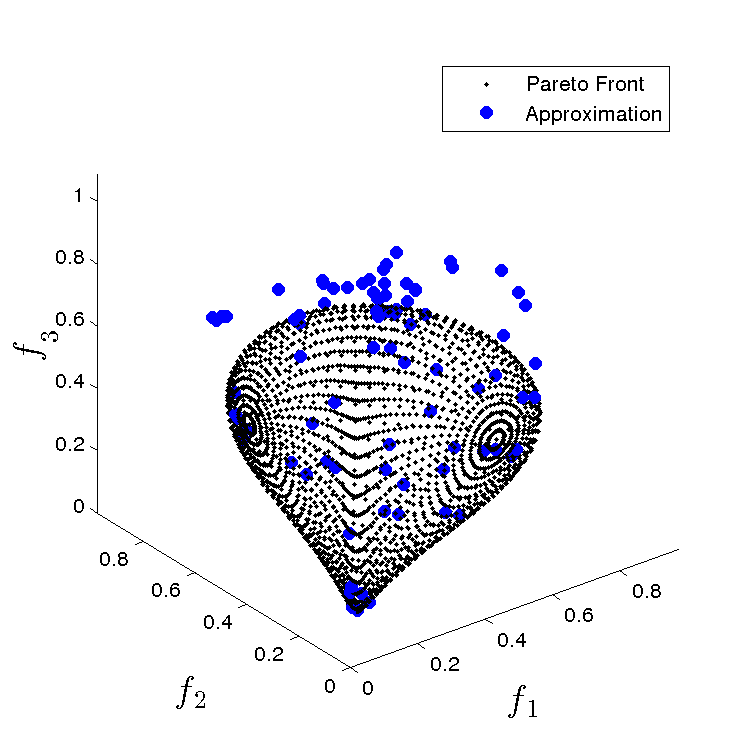


(c) Pareto Set (d) Pareto Front

Figure 12 PPS approximations on Eq-DTLZ2 and a budget of 500,000 functions evaluations.

Table 1 presents the performance indicator values ($\Delta_{2}$ and HV) and the ratio of feasible solutions for Eq-DTLZ4 with different number of objectives and equality constraints for a long run (1,000,000 functions evaluations).

Table 1: Performance for the long run of Eq-DTLZ4 of the different MOEAs.

| **METHOD** | $\Delta_{2}$ | **HV** | **RATIO OF FEASIBILITY** |
| --- | --- | --- | --- |
| **EQ-DTLZ4 (M= 3 and p=1)** | | | |
| **ANSGA-III** | 2.8724e-04 | 2.1059e-10 | 1.00 |
| **GDE3** | 4.3166e-01 | 2.1056e-10 | 1.00 |
| **MOEADD** | 1.2032e-05 | 2.0252e-10 | 1.00 |
| **NSGA-II** | 4.5451e-04 | 2.1059e-10 | 0.99 |
| **NSGA-III** | 2.8724e-04 | 2.1059e-10 | 1.00 |
| **PPS** | 5.4675e-03 | 2.1058e-10 | 0.99 |
| **EQ-DTLZ4 (M= 4 and p=1)** | | | |
| **ANSGA-III** | 4.9884e-03 | 8.9390e-15 | 1.00 |
| **GDE3** | 7.8509e-01 | 8.8768e-15 | 1.00 |
| **MOEADD** | 5.7475e-06 | 8.6261e-15 | 1.00 |
| **NSGA-II** | 4.0930e-03 | 8.9390e-15 | 1.00 |
| **NSGA-III** | 4.9884e-03 | 8.9390e-15 | 1.00 |
| **PPS** | 1.2327e-02 | 7.4647e-15 | 1.00 |
| **EQ-DTLZ4 (M= 4 and p=2)** | | | |
| **ANSGA-III** | 4.0995e-04 | 1.7954e-20 | 0.98 |
| **GDE3** | 1.0044e+00 | 0.0000e+00 | 0.06 |
| **MOEADD** | 4.3665e-04 | 1.3263e-20 | 1.00 |
| **NSGA-II** | 1.8104e-03 | 1.8035e-20 | 1.00 |
| **NSGA-III** | 4.0995e-04 | 1.7954e-20 | 0.98 |
| **PPS** | 9.5984e-03 | 1.7933e-20 | 1.00 |

Figure 13 shows the Pareto set/fronts approximations of Eq-DTLZ4 with M=3 and p=1 for: (a) ANSGA-III, (b) GDE3, (c) MOEA/D/D, (d) NSGA-II, (e) NSGA-III and (f) PPS for the long run (1,000,000 function calls).


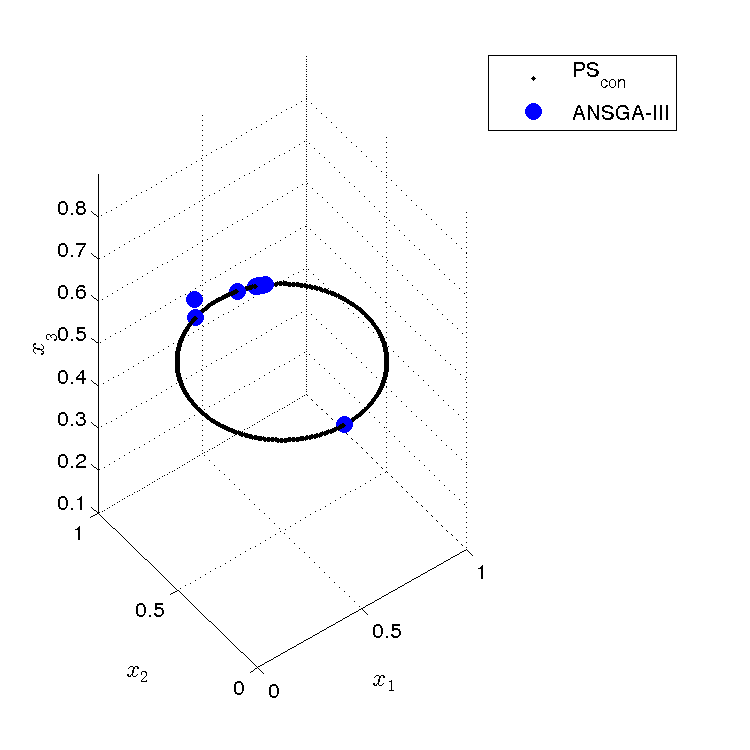

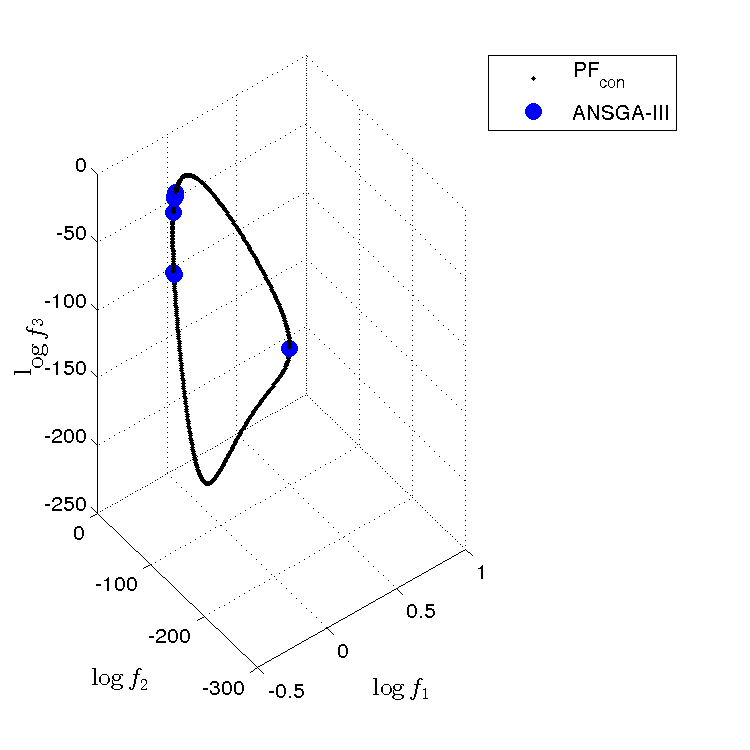


1. A-NSGAIII


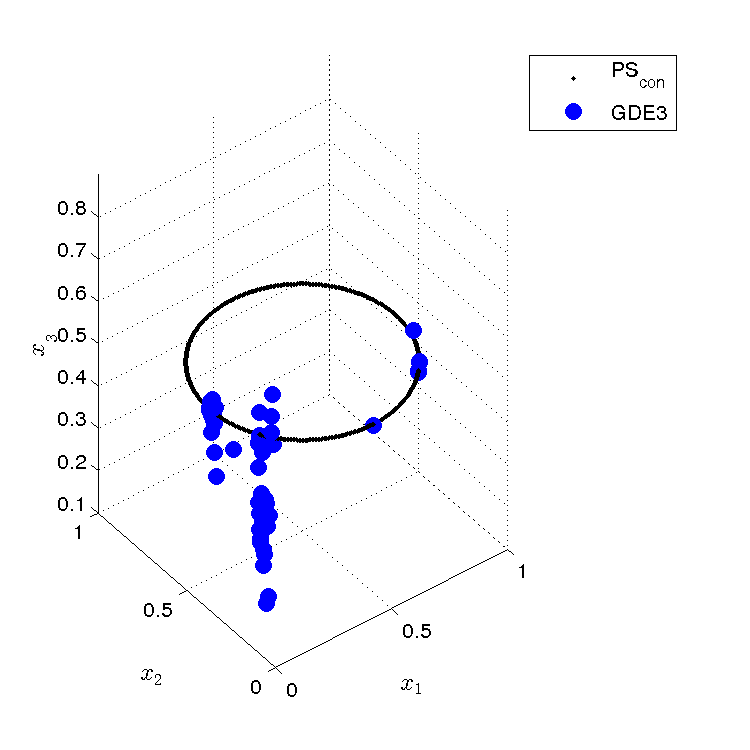

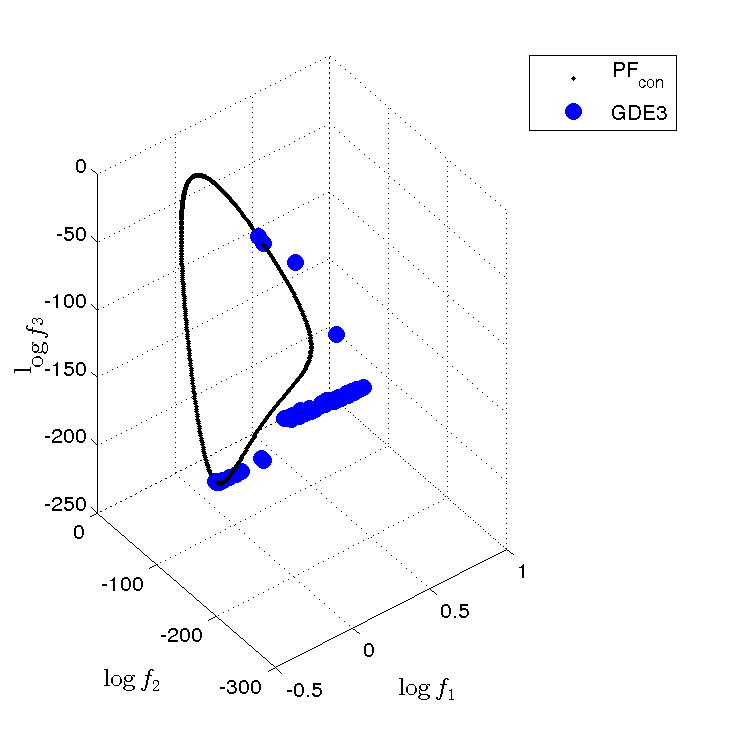


1. GDE3


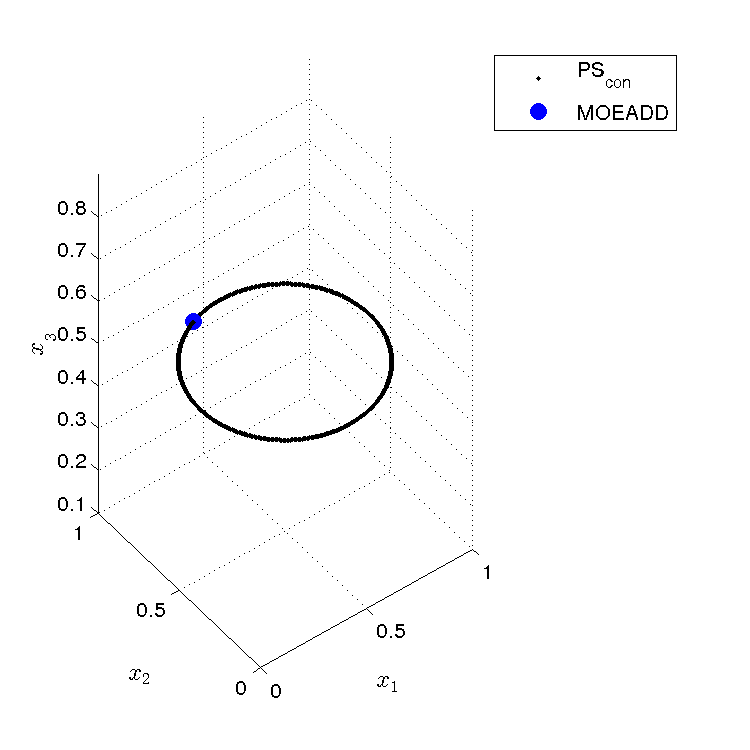

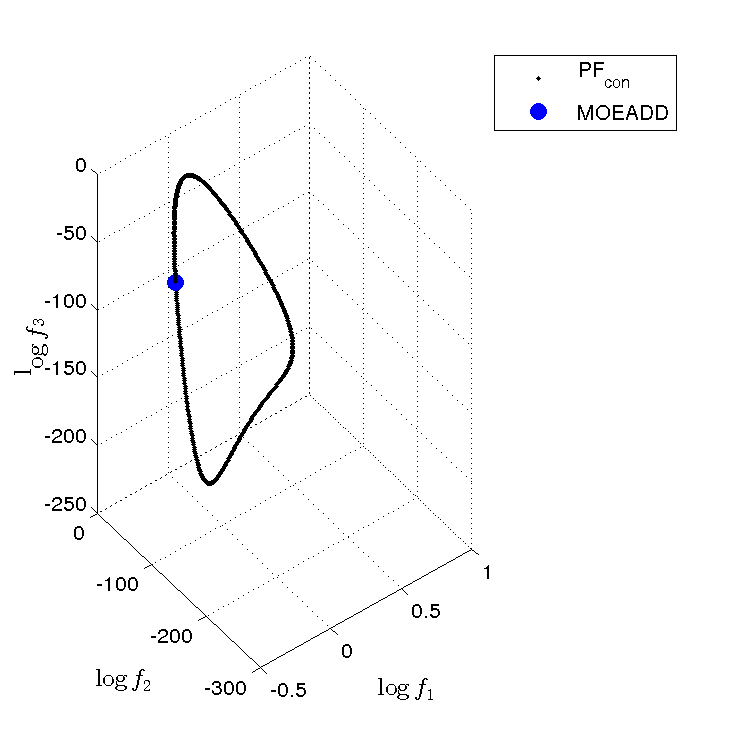


1. MOEA/D/D


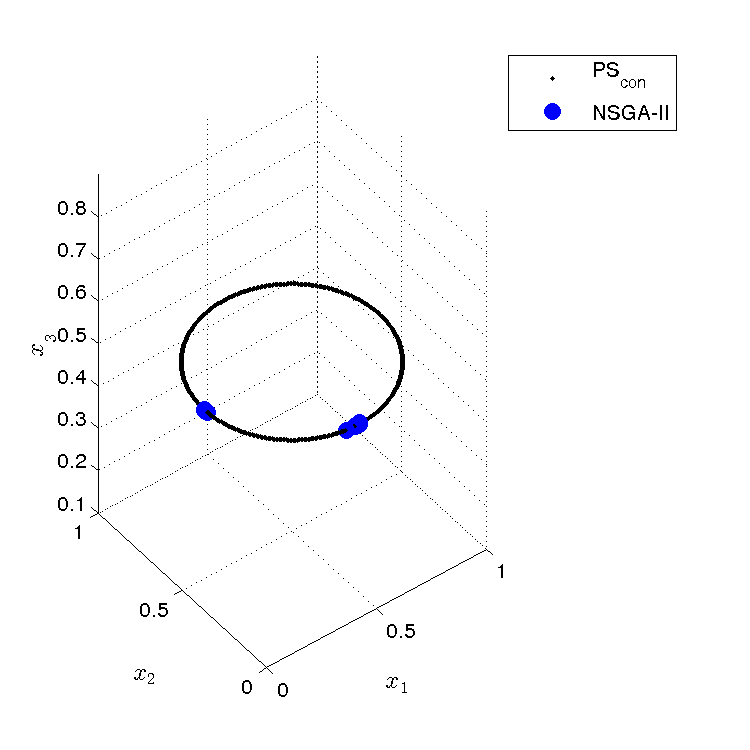

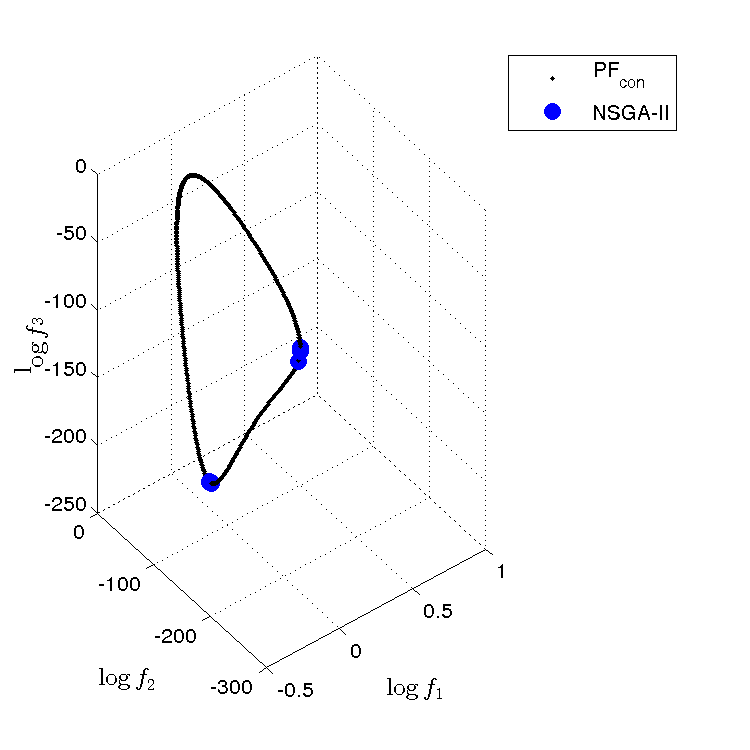


1. NSGA-II


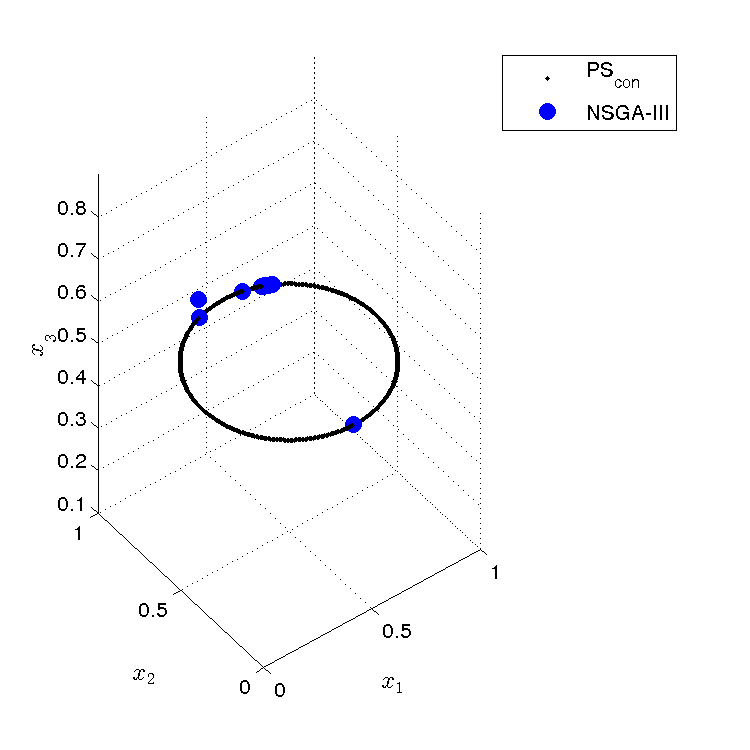

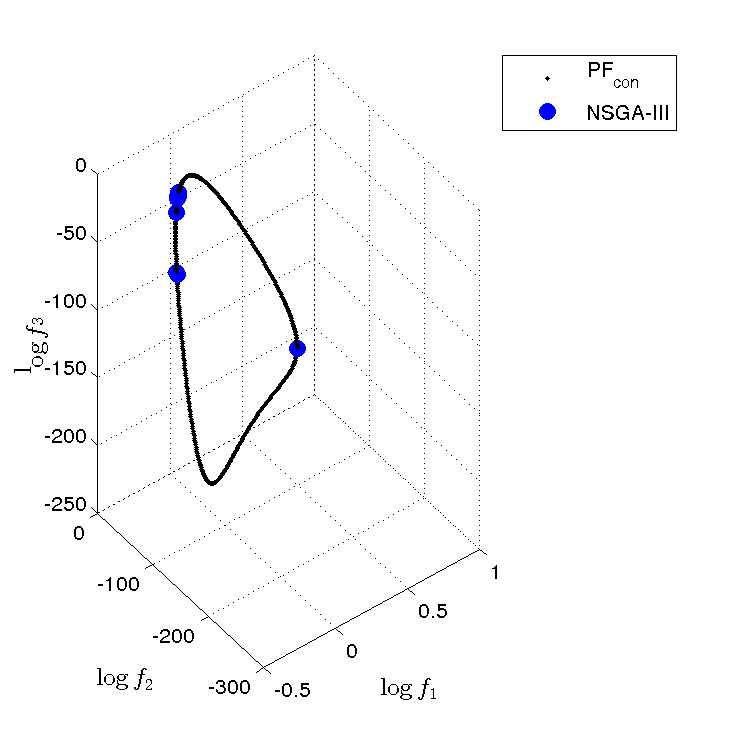


1. NSGA-III


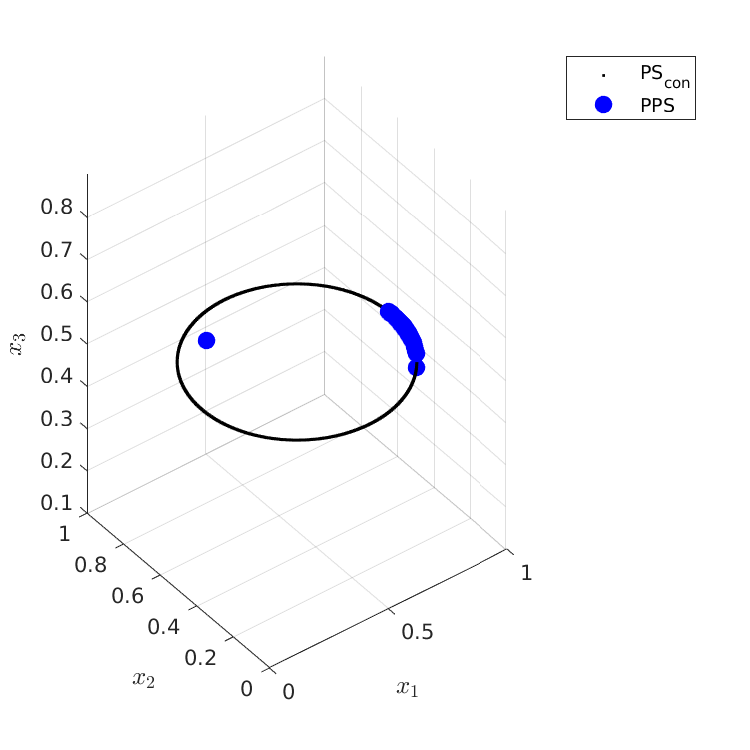

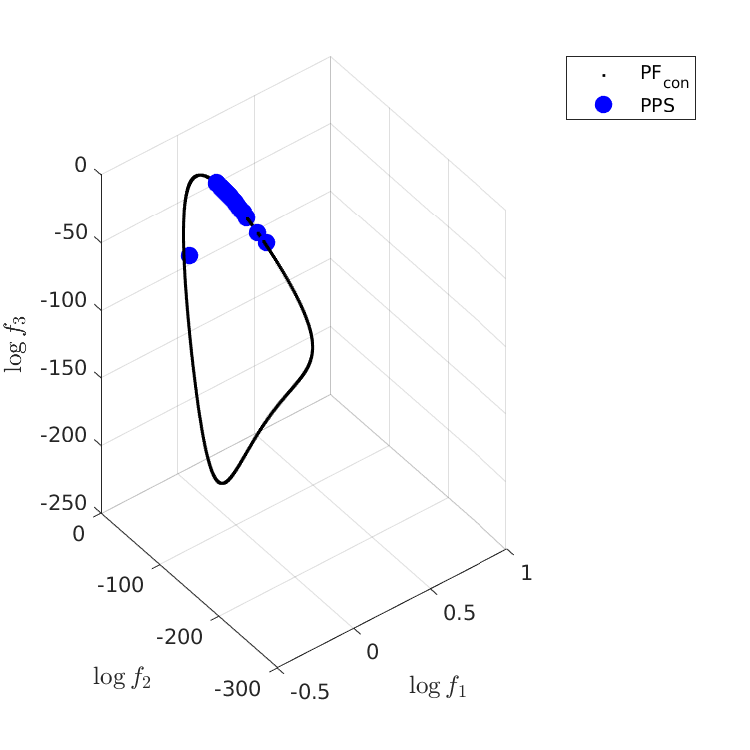


1. PPS

Figure 14 shows the Pareto set/fronts approximations of Eq-DTLZ4 with M=4 and p=1 for: (a) ANSGA-III, (b) GDE3, (c) MOEA/D/D, (d) NSGA-II, (e) NSGA-III and (f) PPS for the long run (1,000,000 function calls).


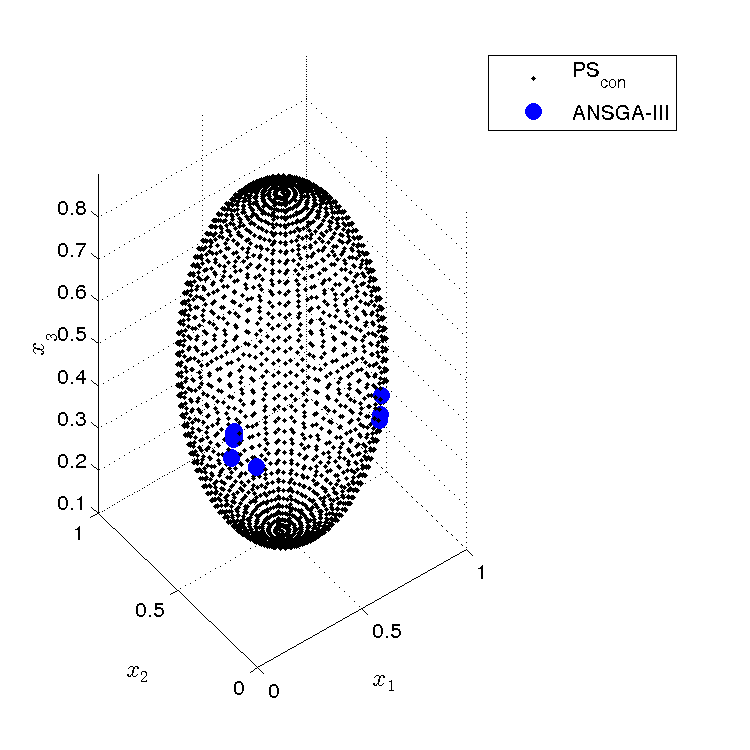

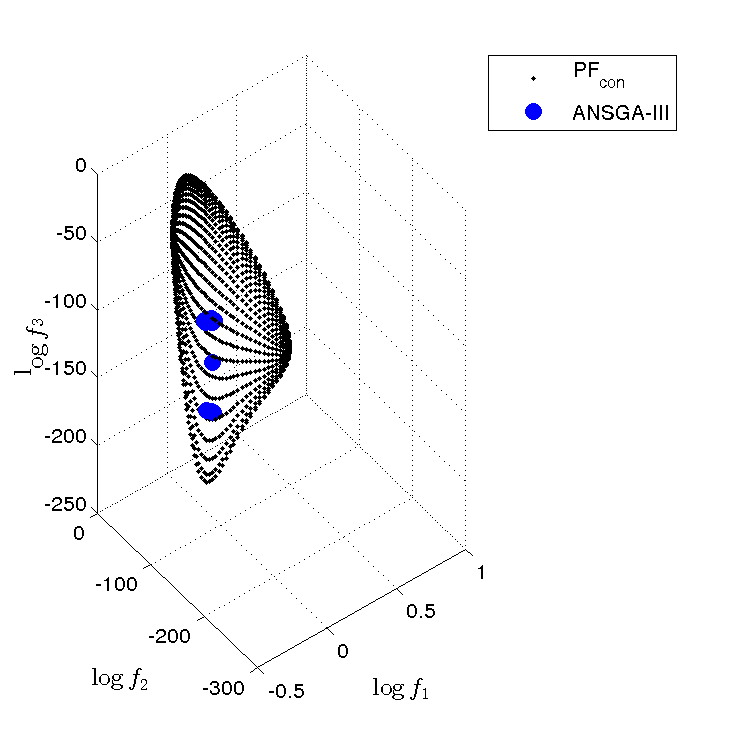


1. A-NSGAIII


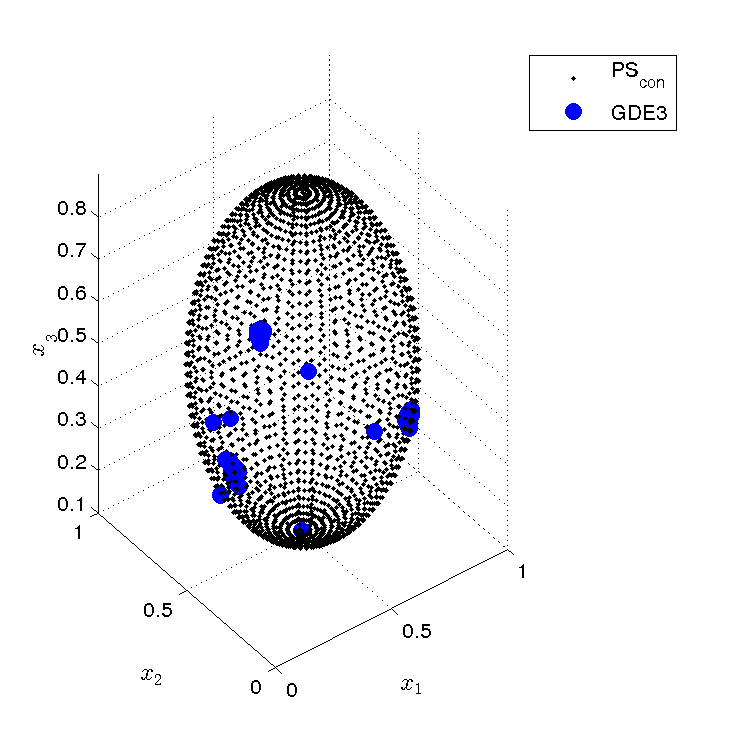

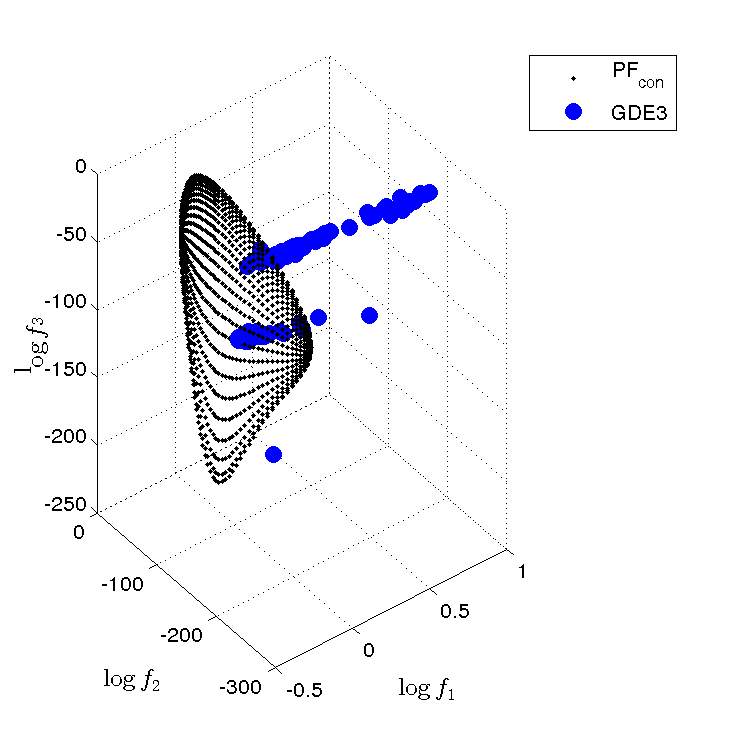


1. GDE3


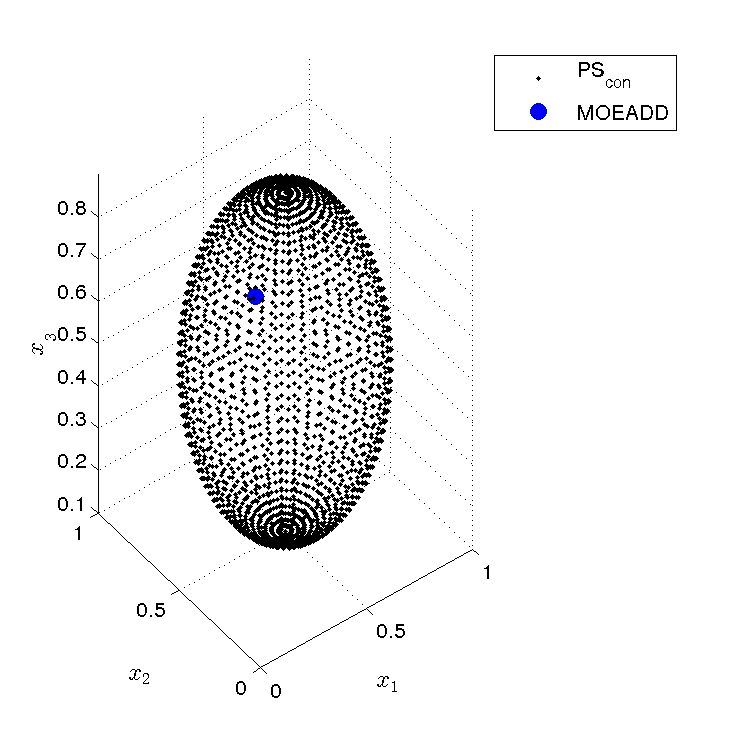

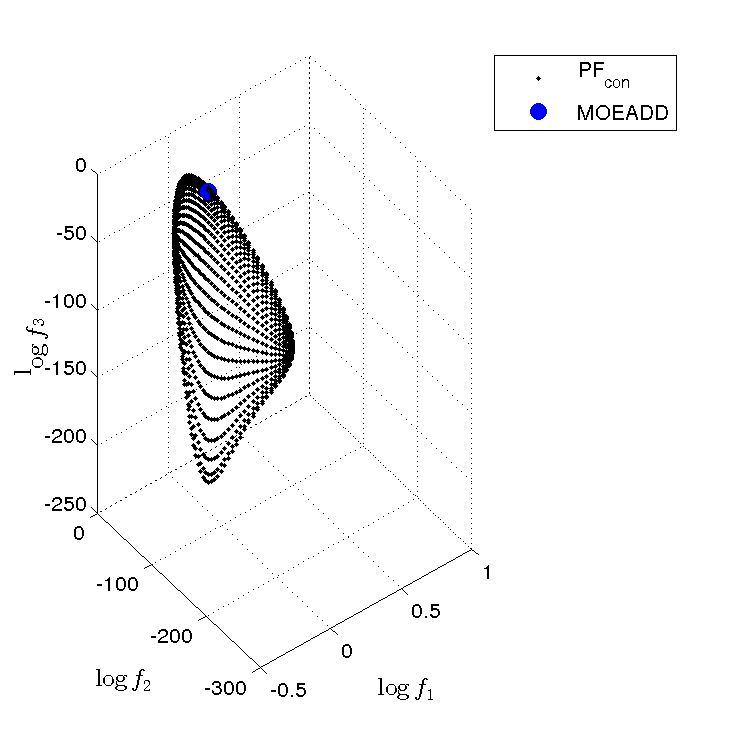


1. MOEA/D/D


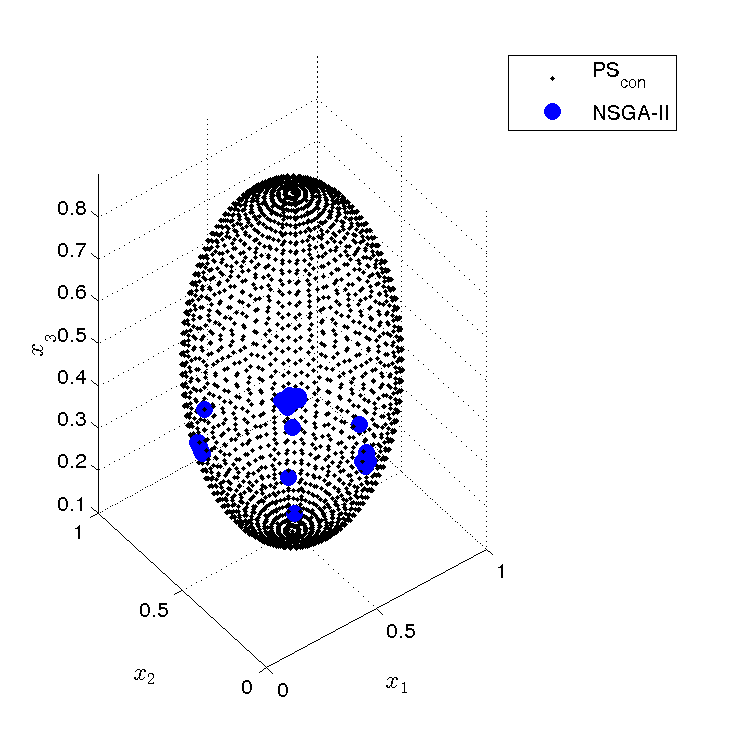

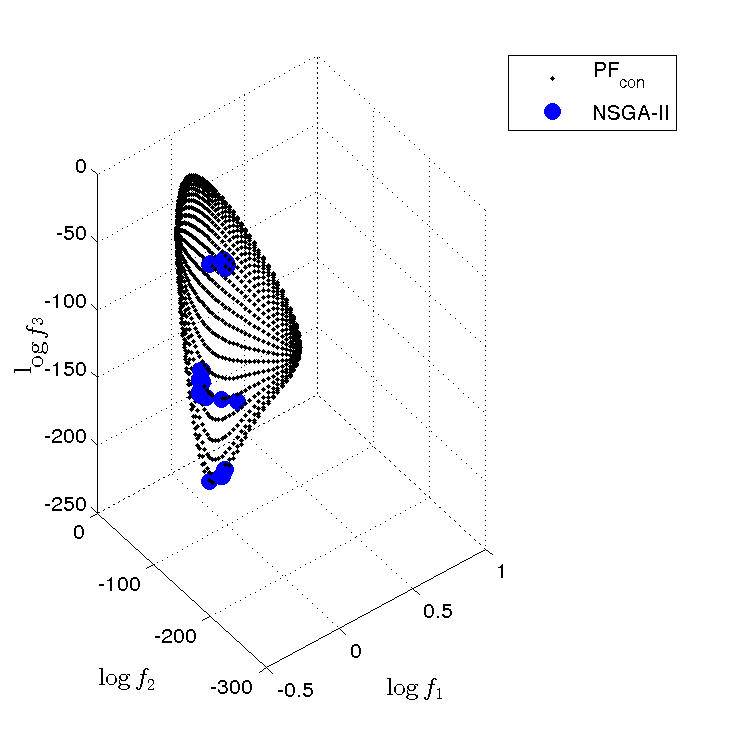


1. NSGA-II


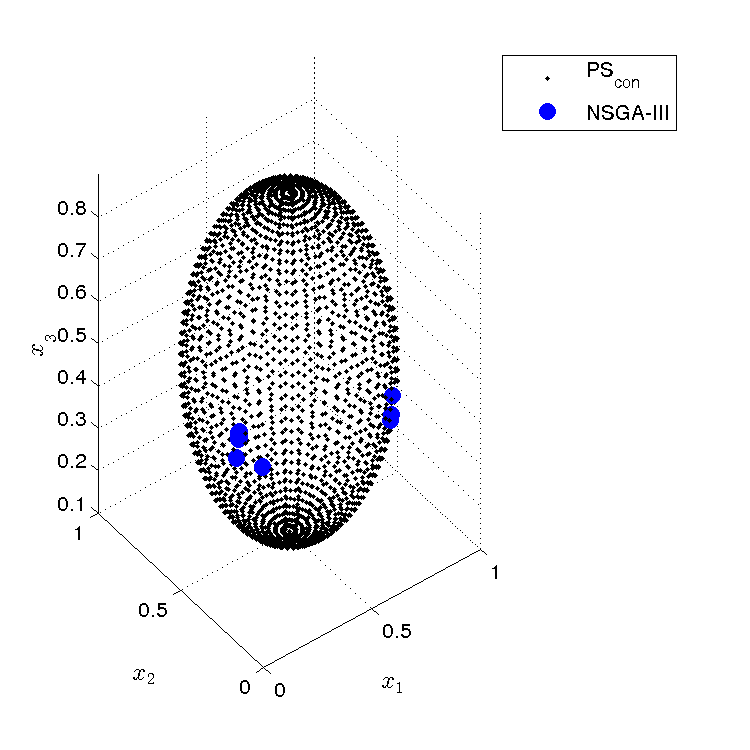

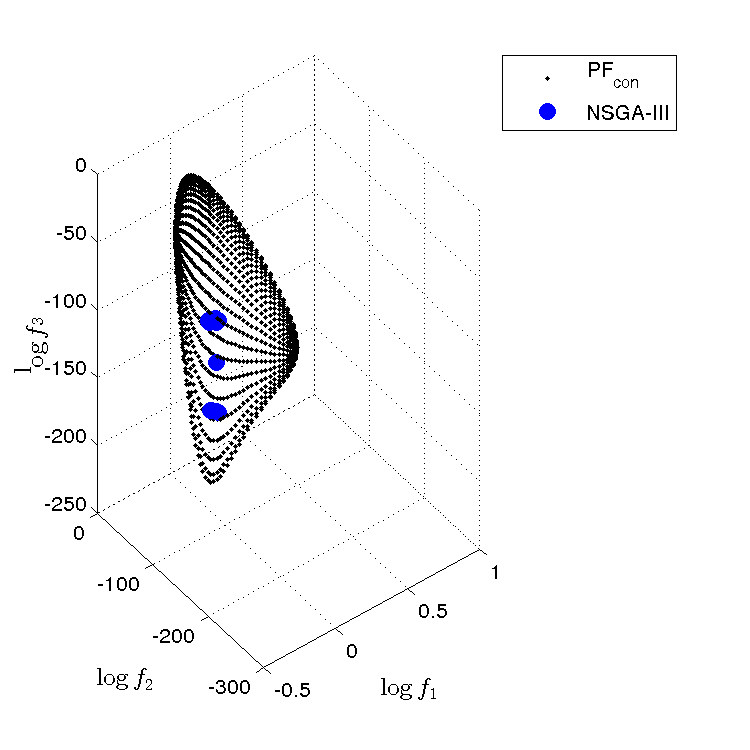


1. NSGA-III


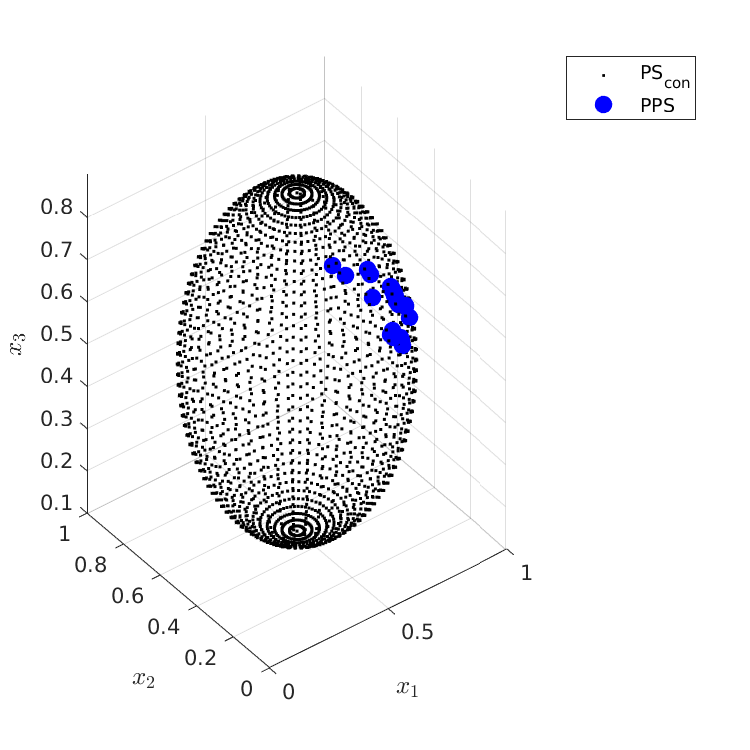

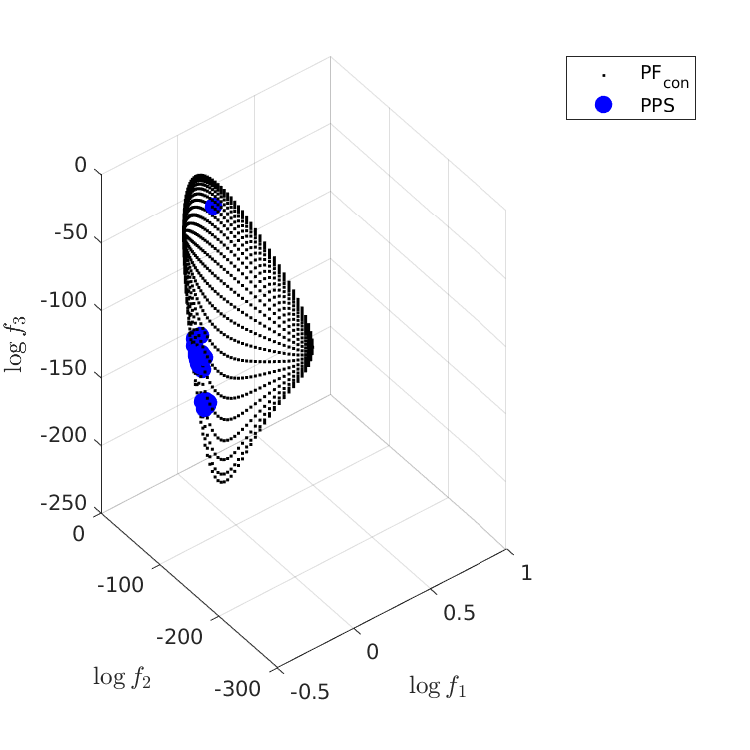


1. PPS

**Experimental Design, Materials, and Methods**

We have selected six state-of-the-art MOEAs in order to test the proposed benchmark. For all experiments we use the PlatEMO framework [2], where we have executed 30 independent runs of the following MOEAs: NSGA-II, NSGA-III, Adaptive NSGA-III, MOEA/ D/ D, GDE3 and PPS; using 50, 000, 100, 000 and 150, 000 function calls for MOPs with M = 3; and using 200, 000, 300, 000 and 500, 000 function calls for MOPs with M = 4. For the long run, we execute all the selected MOEAs for 1,000,000 function evaluations for Eq-DTLZ4 with different number of objectives and constraints We have selected six state-of-the-art MOEAS in order to test the proposed benchmark. For all experiments we use the PlatEMO framework [2], where we have executed 30 independent runs of the following MOEAs: NSGA-II, NSGA-III, Adaptive NSGA-III, MOEA/ D/ D, GDE3 and PPS; using 50, 000, 100, 000 and 150, 000 function calls for MOPs with M = 3; and using 200, 000, 300, 000 and 500, 000 function calls for MOPs with M = 4. For the long run, we execute all the selected MOEAs for 1,000,000 function evaluations for Eq-DTLZ4 with different number of objectives and constraints. We also use the Kruskal-Wallis test as statistical significance proof to validate the numerical approximations. For this, we apply the test to each function considering the different algorithms as the groups and each run as the sample. We set$\propto=0.05$; if the $p$ value of the test is less than $\propto$ then we reject the null hypothesis, which is that the sample data from each group comes from the same distribution, for more details see [1].

**Acknowledgments**

The authors acknowledge funding from the Conacyt Basic Science project No. 285599, SEP Cinvestav project No. 231, and IPN SIP project No. 20196444.

**References**

[1] Oliver Cuate, Lourdes Uribe, Adriana Lara, and Oliver Schütze. *A Benchmark for Equality Constrained Multi-objective Optimization,* Swarm and Evolutionary Computation. DOI: [https://doi.org/10.1016/j.swevo.2019.100619](https://doi.org/10.1016/j.swevo.2019.100619?fbclid=IwAR1gF8TX2sYhDxoba4Ttc4gyqKk3RKr8eNfpnP8LXVUqMMs_1vr1a53J3kc) in press.

[2] Ye Tian, Ran Cheng, Xingy Zhang, and Yaochu Jin. *PlatEMO: A Matlab Platform for Evolutionary Multi-Objective Optimization,* IEEE Computational Intelligence Magazine, 2017, 12(4): 73-87.
