# Supplementary figures and images for: Dataset on a Benchmark for Equality Constrained Multi-objective Optimization
Source: Data Brief. 2020 Jan 11;29:105130. doi: 10.1016/j.dib.2020.105130 (PMC6994572; doi:10.1016/j.dib.2020.105130)

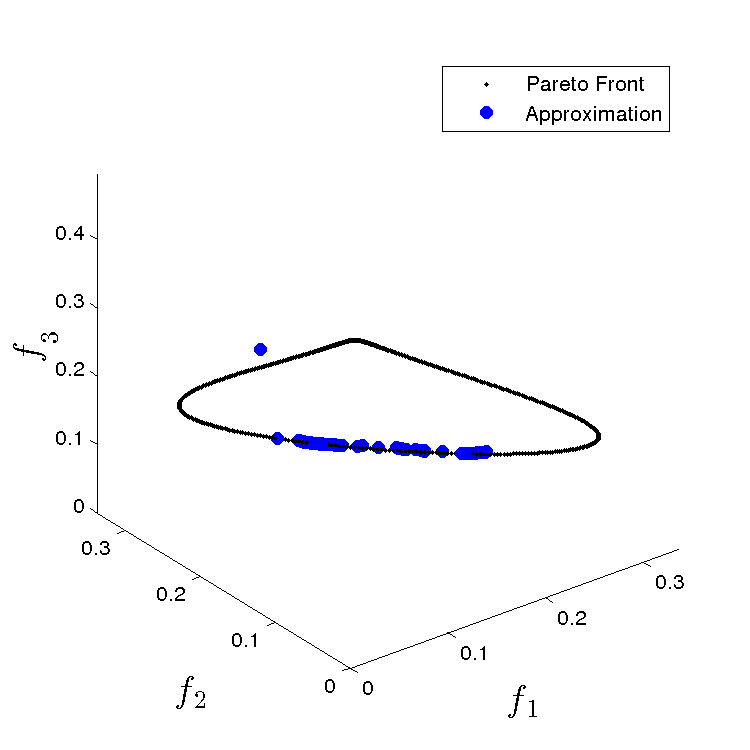

Supplement: Multimedia component 1 [file mmc1.zip › DataInBrief/Images/pf_DP_PPS_H1_DTLZ1.png]

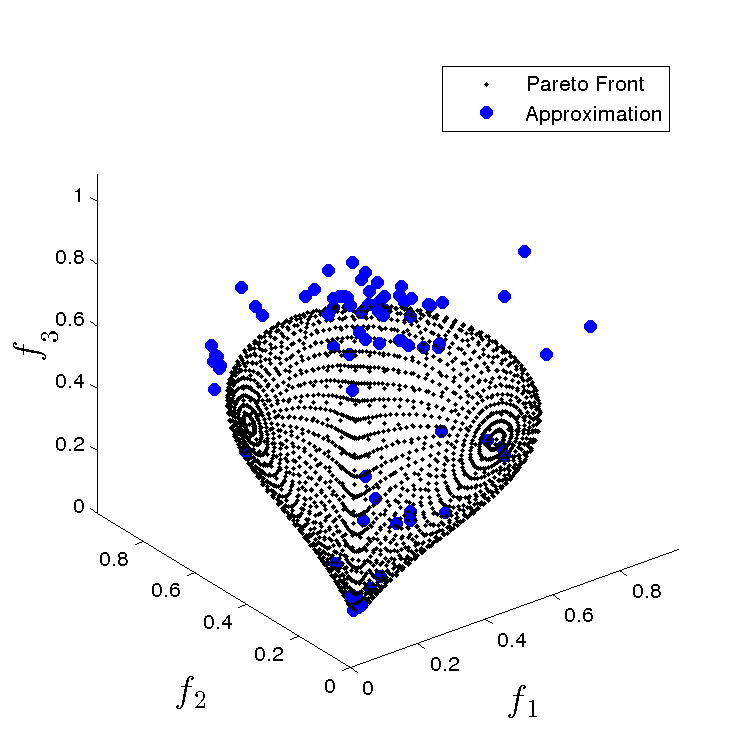

Supplement: Multimedia component 1 [file mmc1.zip › DataInBrief/Images/pf_DP_PPS_H1_DTLZ2.png]

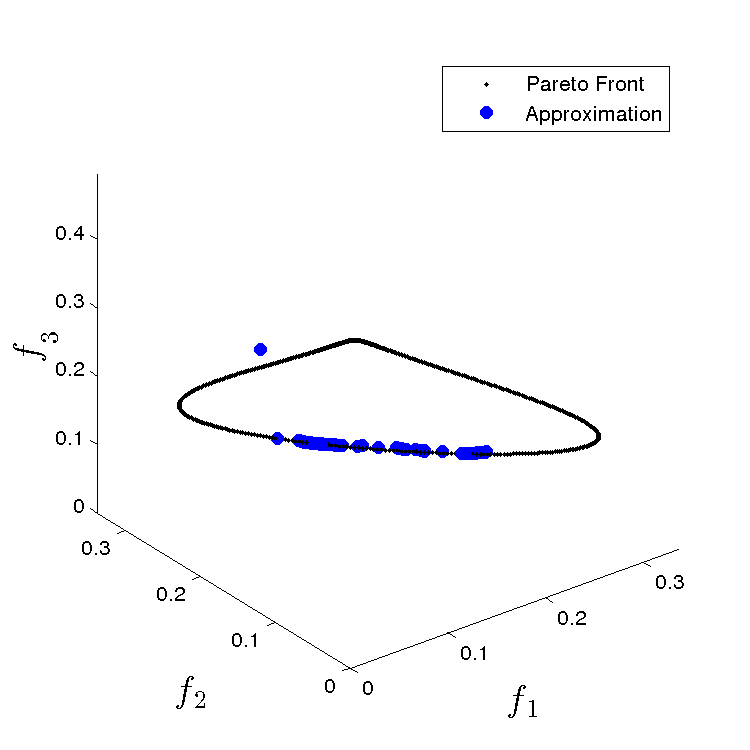

Supplement: Multimedia component 1 [file mmc1.zip › DataInBrief/Images/pf_HV_PPS_H1_DTLZ1.png]

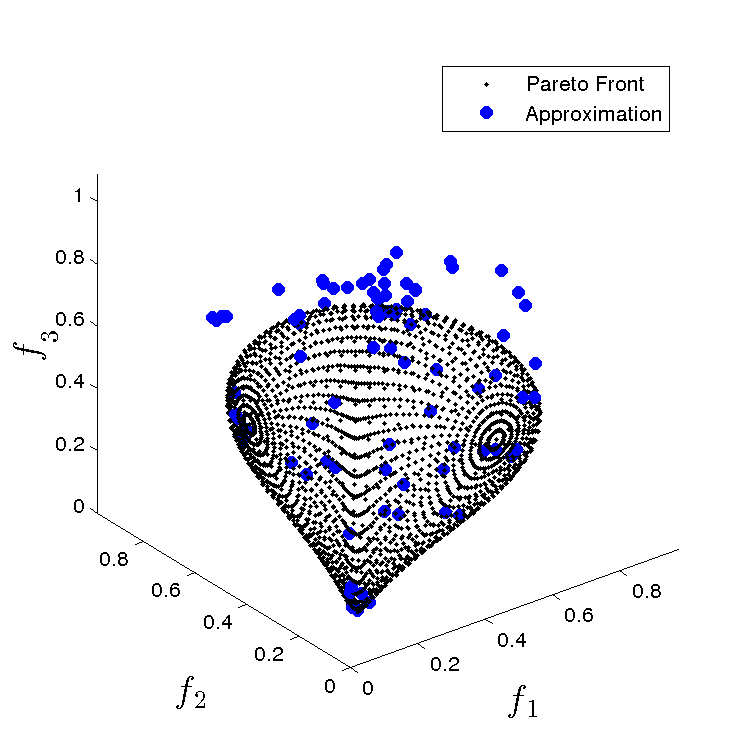

Supplement: Multimedia component 1 [file mmc1.zip › DataInBrief/Images/pf_HV_PPS_H1_DTLZ2.png]

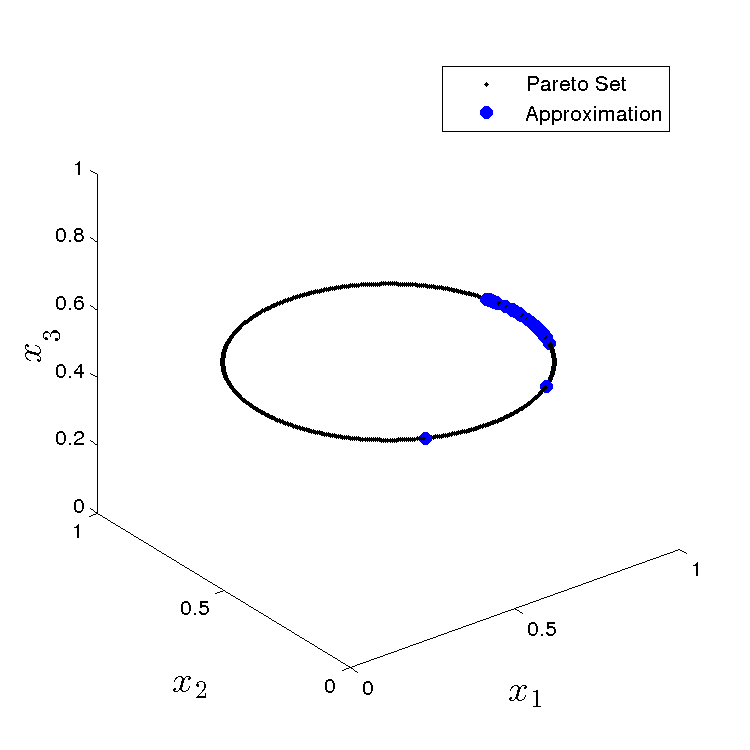

Supplement: Multimedia component 1 [file mmc1.zip › DataInBrief/Images/ps_DP_PPS_H1_DTLZ1.png]

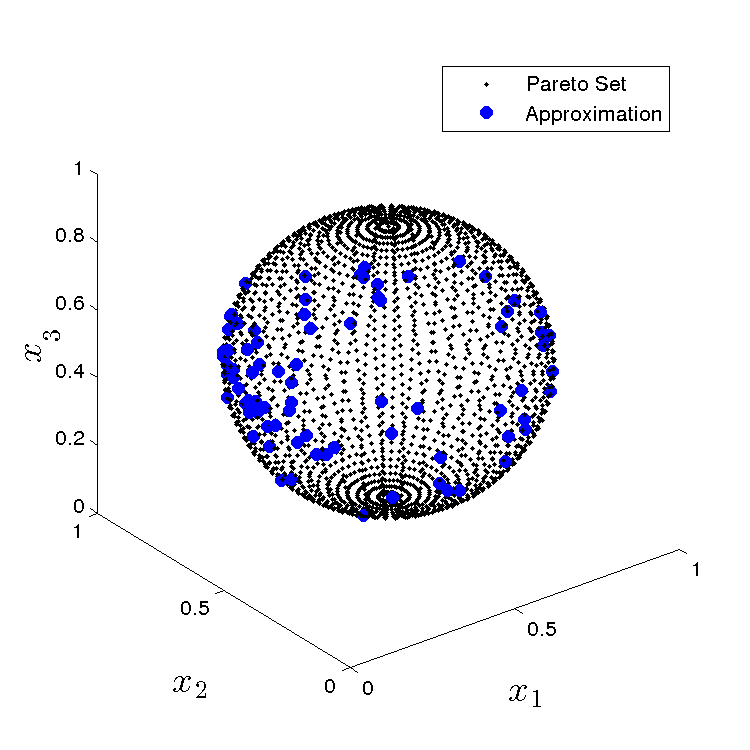

Supplement: Multimedia component 1 [file mmc1.zip › DataInBrief/Images/ps_DP_PPS_H1_DTLZ2.png]

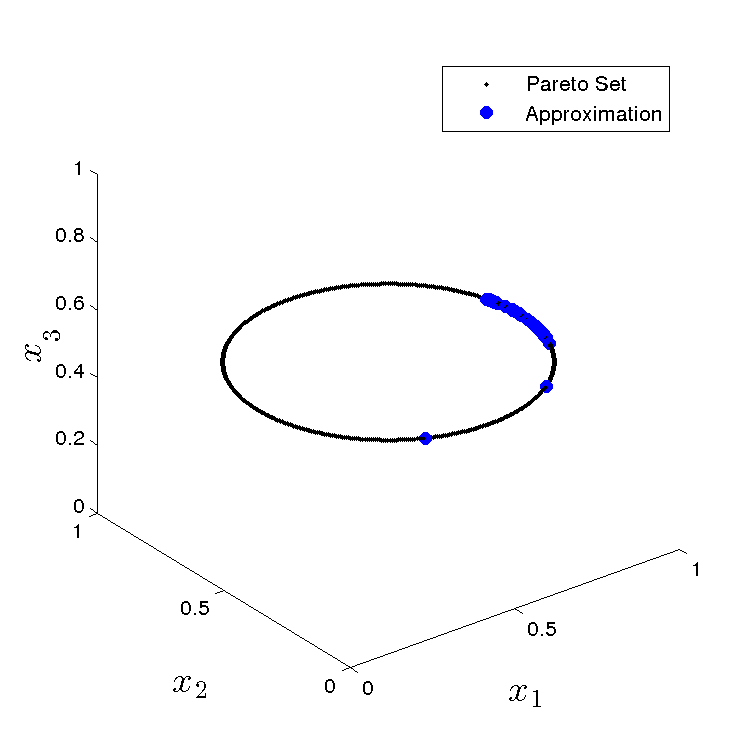

Supplement: Multimedia component 1 [file mmc1.zip › DataInBrief/Images/ps_HV_PPS_H1_DTLZ1.png]

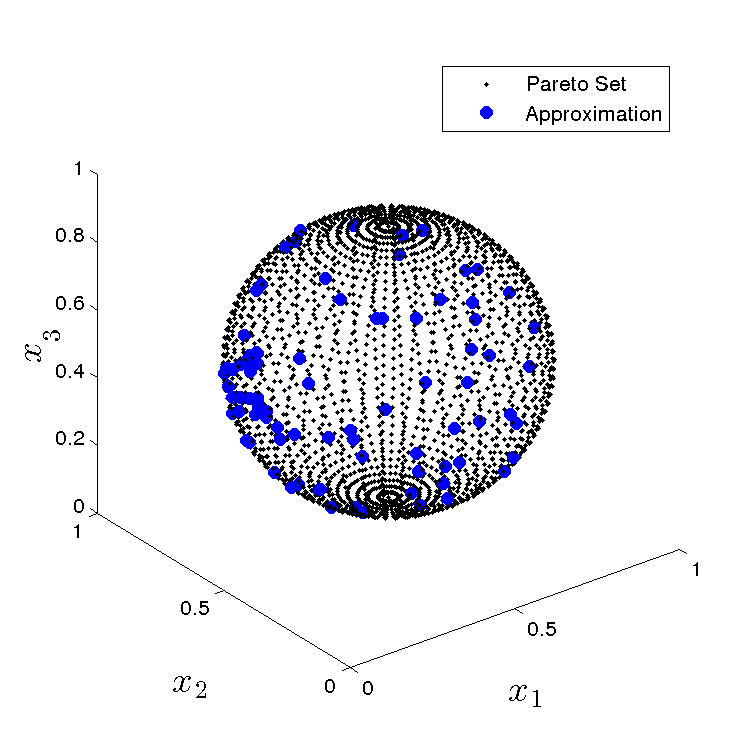

Supplement: Multimedia component 1 [file mmc1.zip › DataInBrief/Images/ps_HV_PPS_H1_DTLZ2.png]

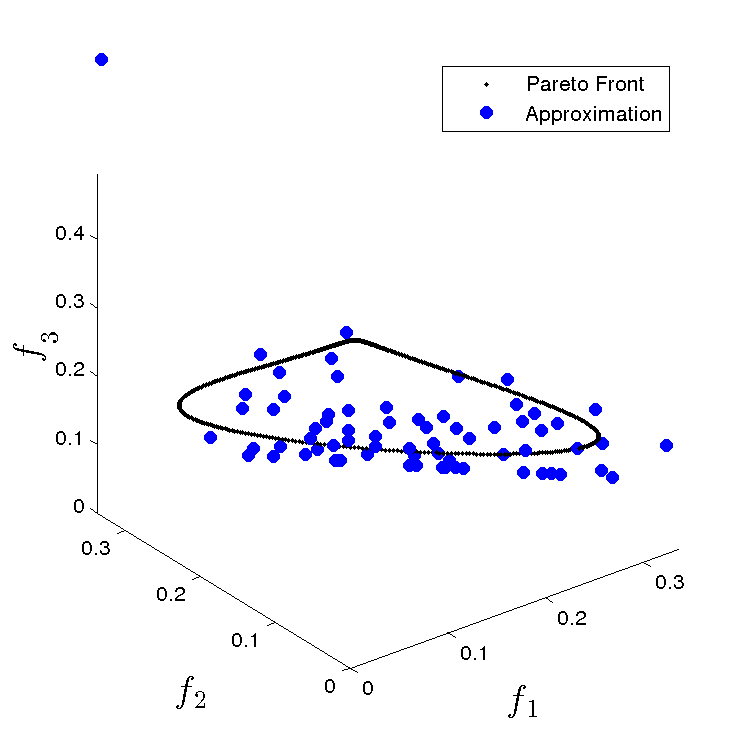

Supplement: Multimedia component 1 [file mmc1.zip › DataInBrief/Images/pf_DP_MTS_H1_DTLZ1_k3.png]

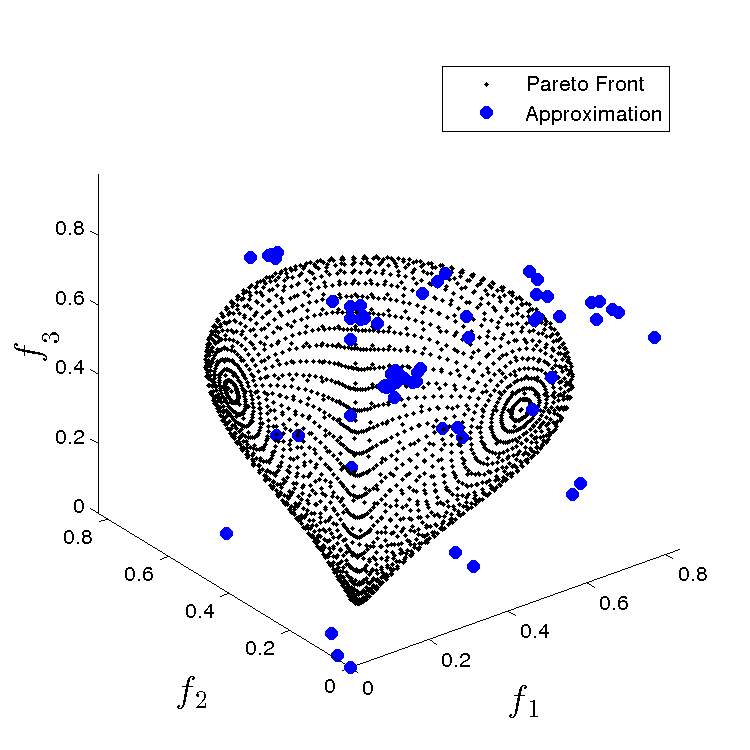

Supplement: Multimedia component 1 [file mmc1.zip › DataInBrief/Images/pf_DP_MTS_H1_DTLZ2_k4.png]

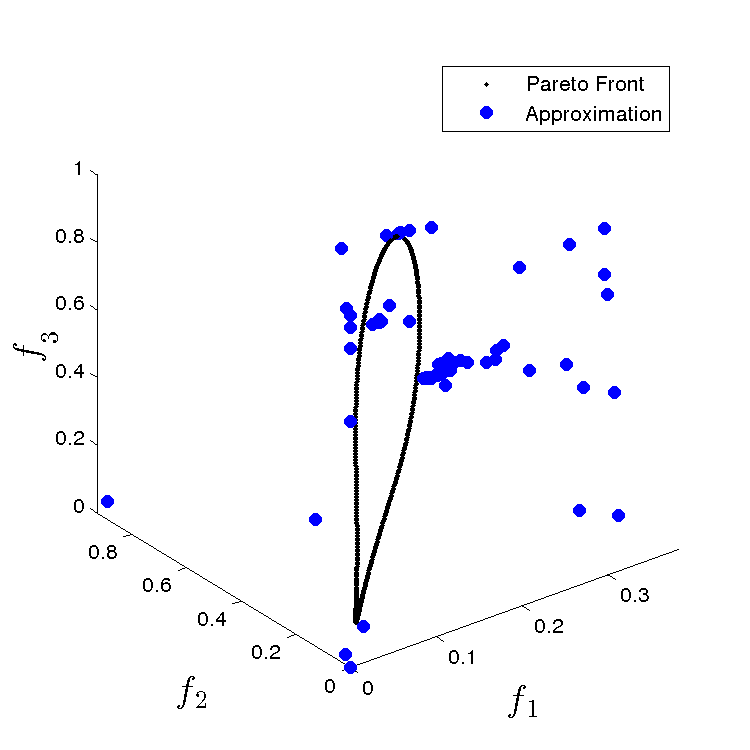

Supplement: Multimedia component 1 [file mmc1.zip › DataInBrief/Images/pf_DP_MTS_H2_DTLZ2_k4.png]

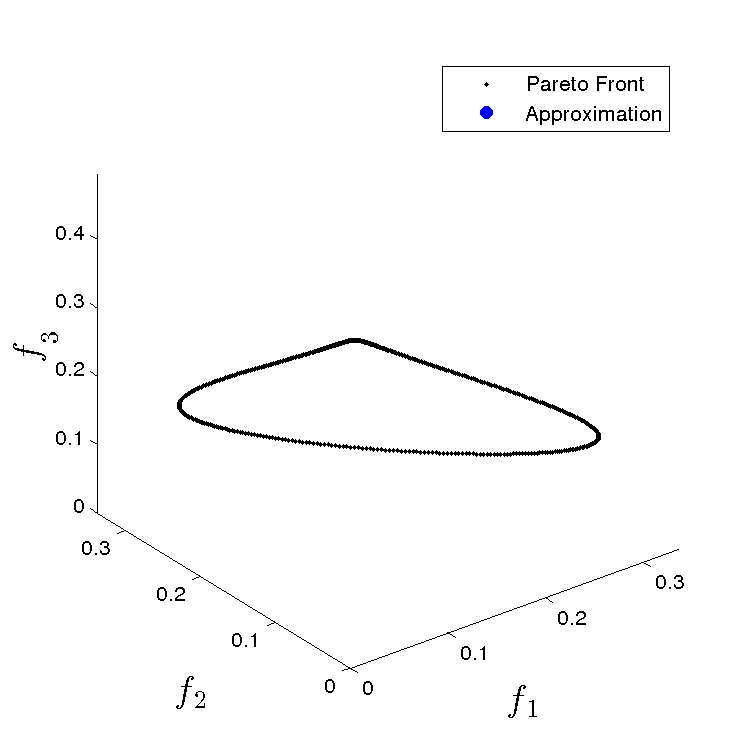

Supplement: Multimedia component 1 [file mmc1.zip › DataInBrief/Images/pf_HV_MTS_H1_DTLZ1_k3.png]

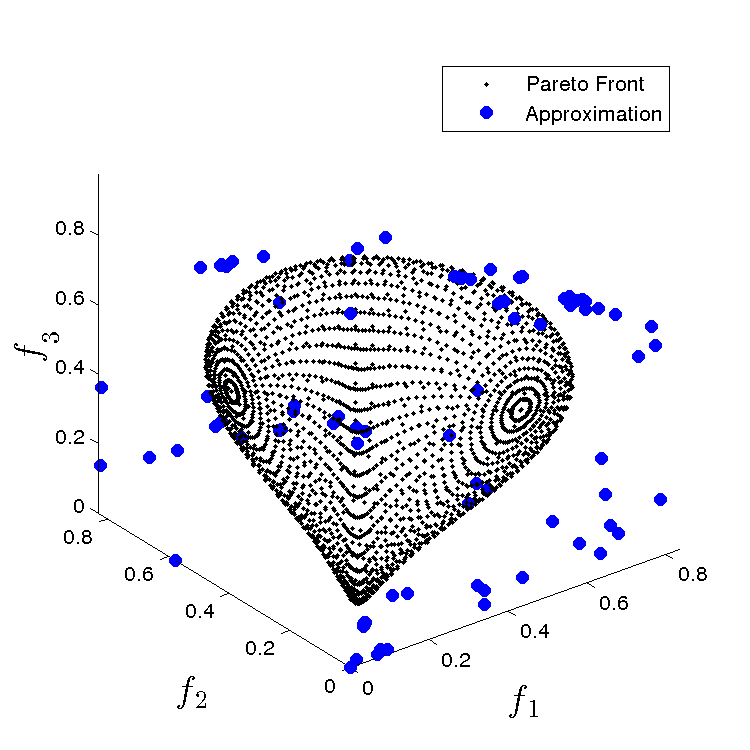

Supplement: Multimedia component 1 [file mmc1.zip › DataInBrief/Images/pf_HV_MTS_H1_DTLZ2_k4.png]

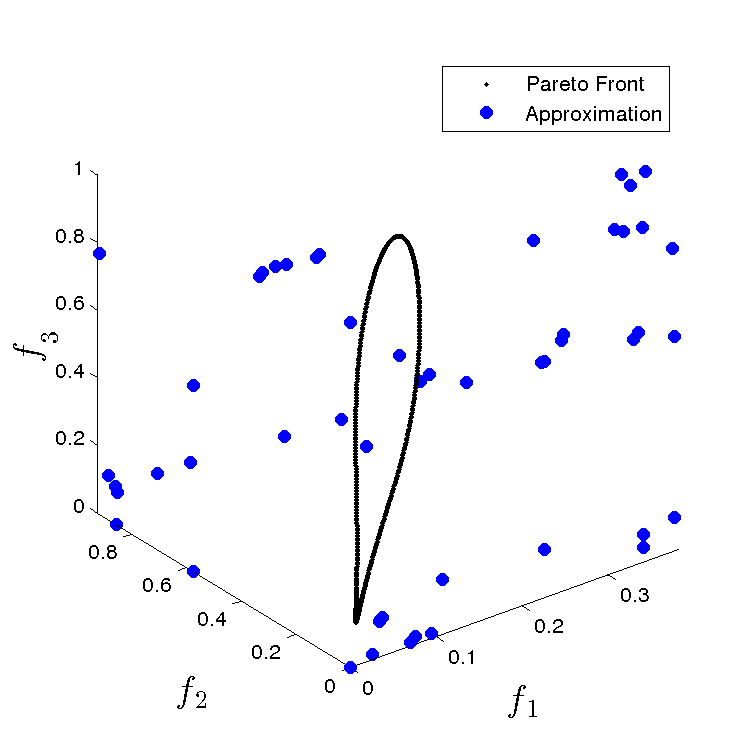

Supplement: Multimedia component 1 [file mmc1.zip › DataInBrief/Images/pf_HV_MTS_H2_DTLZ2_k4.png]

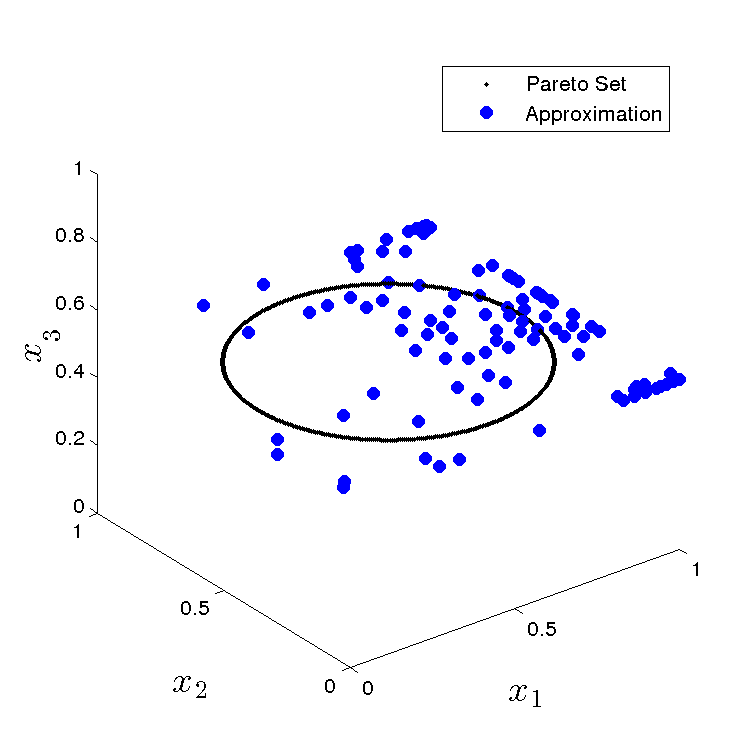

Supplement: Multimedia component 1 [file mmc1.zip › DataInBrief/Images/ps_DP_MTS_H1_DTLZ1_k3.png]

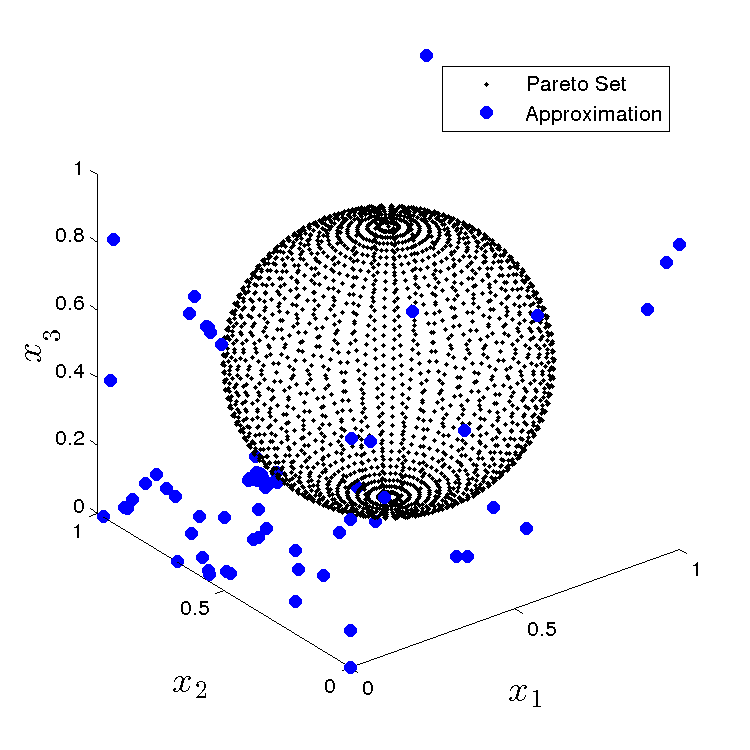

Supplement: Multimedia component 1 [file mmc1.zip › DataInBrief/Images/ps_DP_MTS_H1_DTLZ2_k4.png]

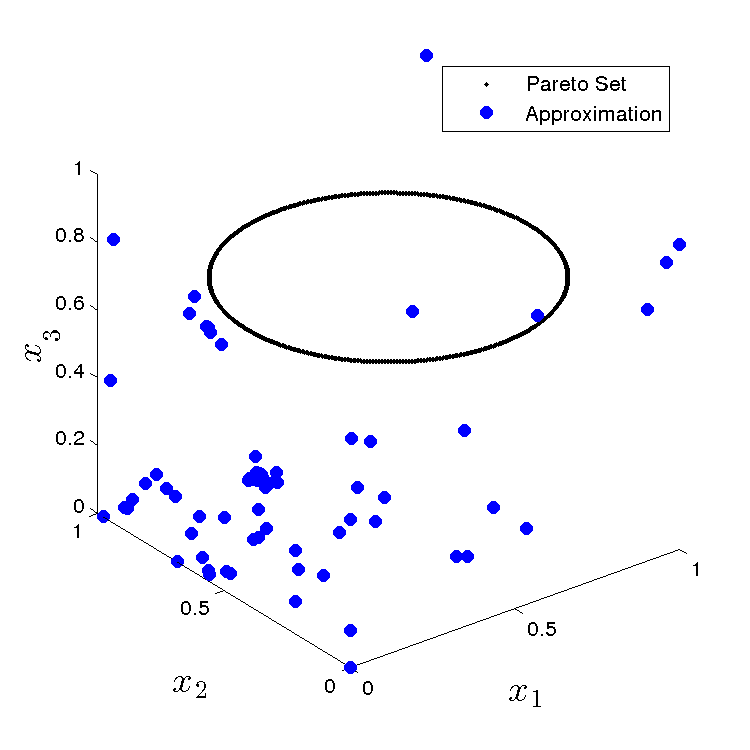

Supplement: Multimedia component 1 [file mmc1.zip › DataInBrief/Images/ps_DP_MTS_H2_DTLZ2_k4.png]

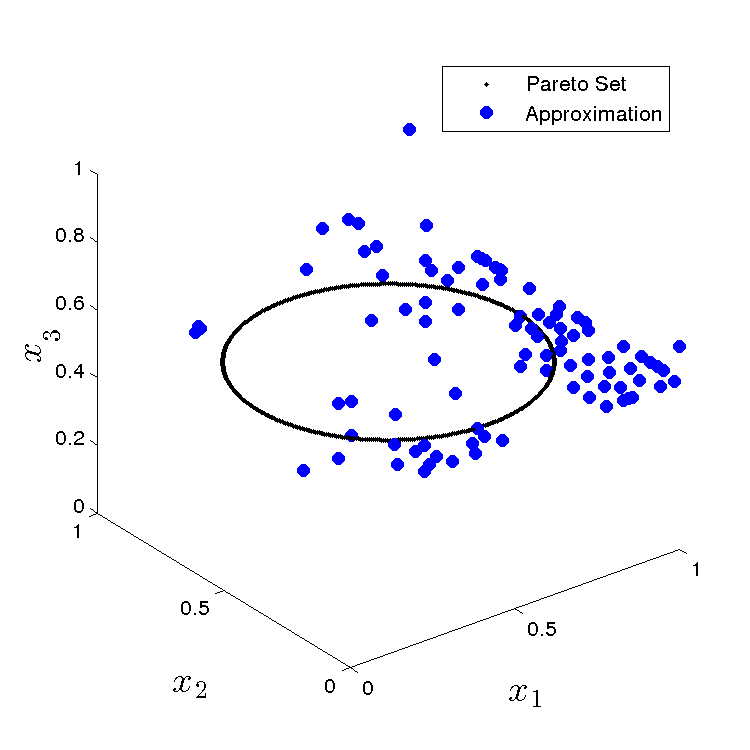

Supplement: Multimedia component 1 [file mmc1.zip › DataInBrief/Images/ps_HV_MTS_H1_DTLZ1_k3.png]

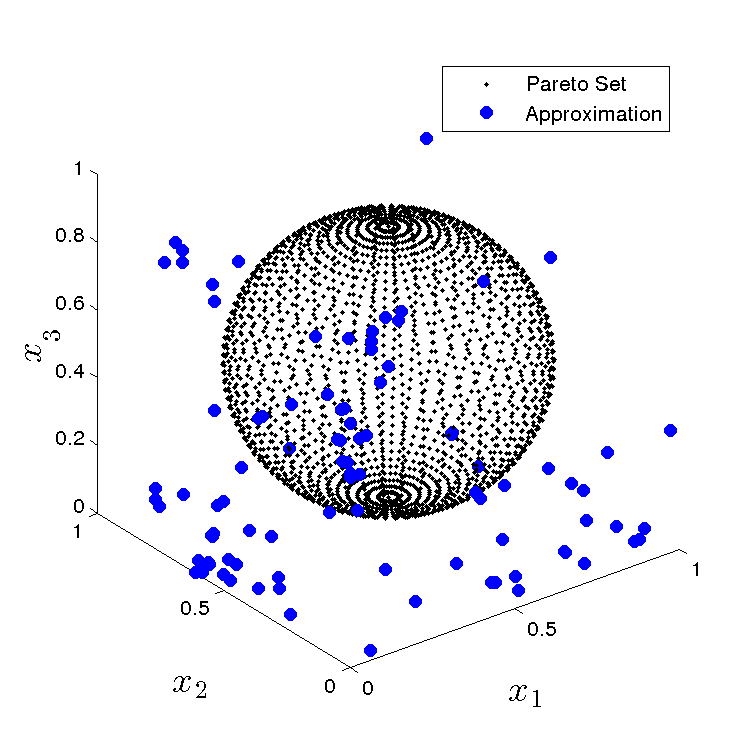

Supplement: Multimedia component 1 [file mmc1.zip › DataInBrief/Images/ps_HV_MTS_H1_DTLZ2_k4.png]

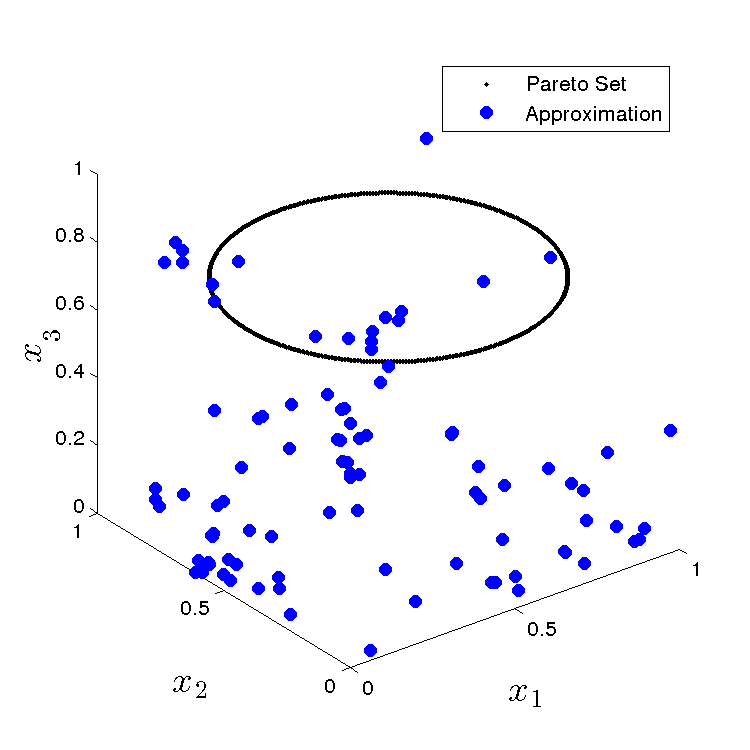

Supplement: Multimedia component 1 [file mmc1.zip › DataInBrief/Images/ps_HV_MTS_H2_DTLZ2_k4.png]

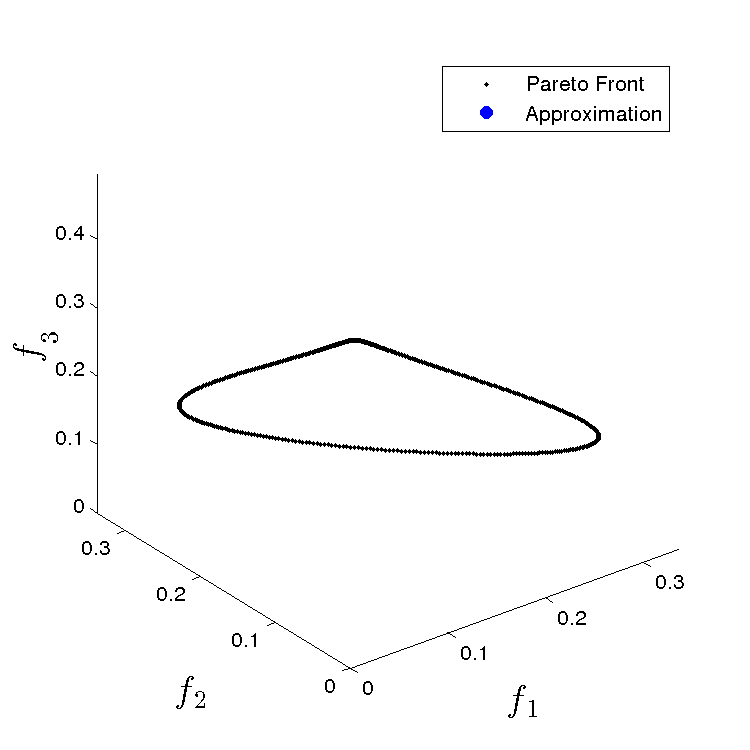

Supplement: Multimedia component 1 [file mmc1.zip › DataInBrief/Images/pf_DP_GDE3_H1_DTLZ1_k3.png]

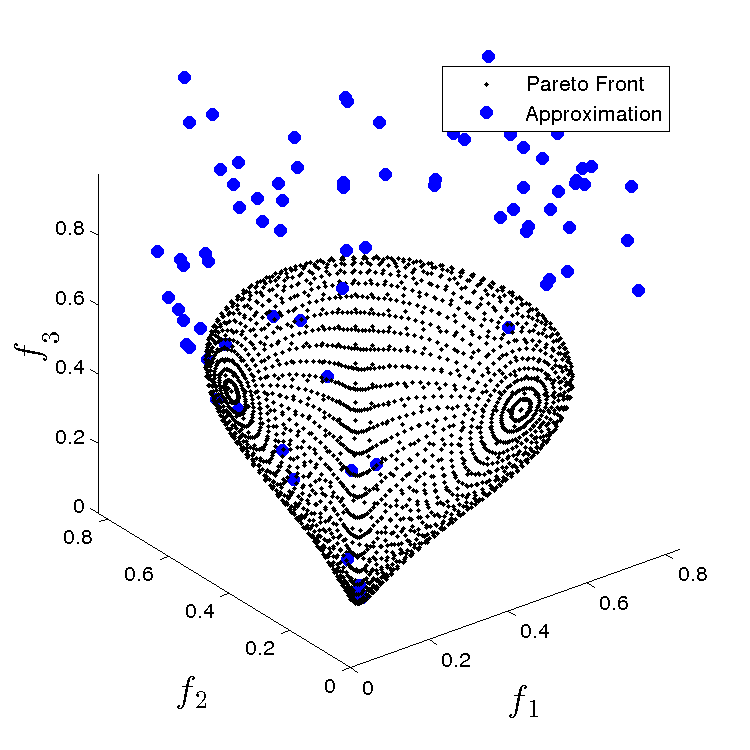

Supplement: Multimedia component 1 [file mmc1.zip › DataInBrief/Images/pf_DP_GDE3_H1_DTLZ2_k4.png]

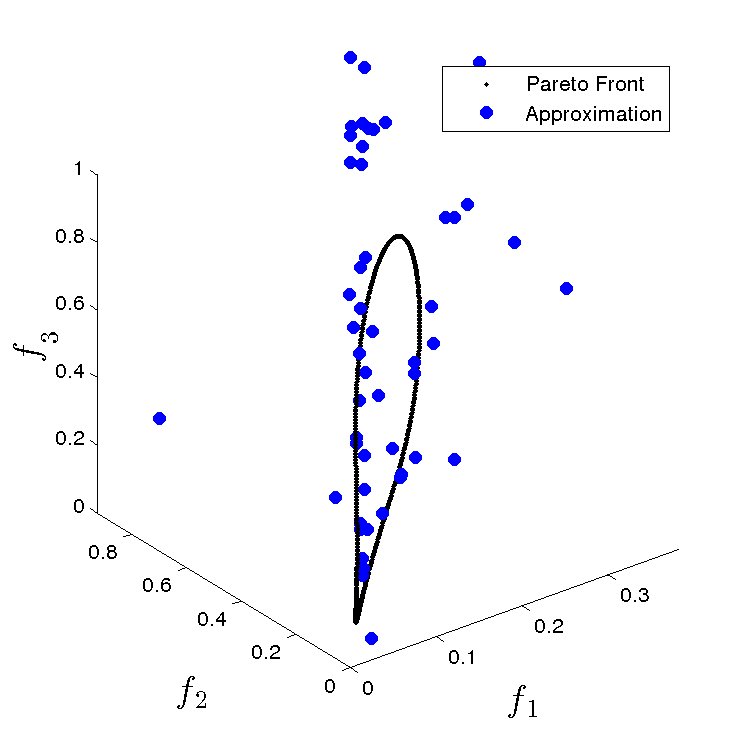

Supplement: Multimedia component 1 [file mmc1.zip › DataInBrief/Images/pf_DP_GDE3_H2_DTLZ2_k4.png]

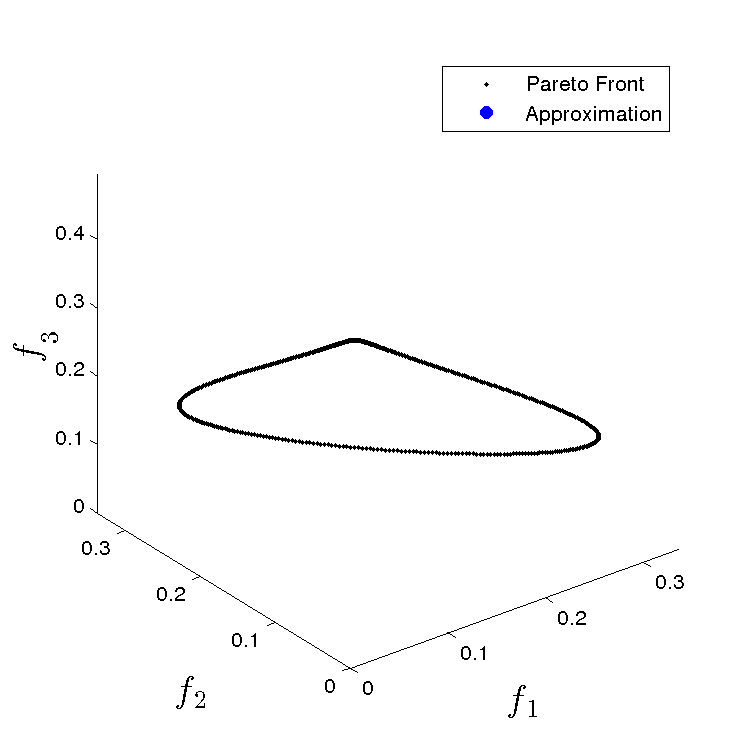

Supplement: Multimedia component 1 [file mmc1.zip › DataInBrief/Images/pf_HV_GDE3_H1_DTLZ1_k3.png]

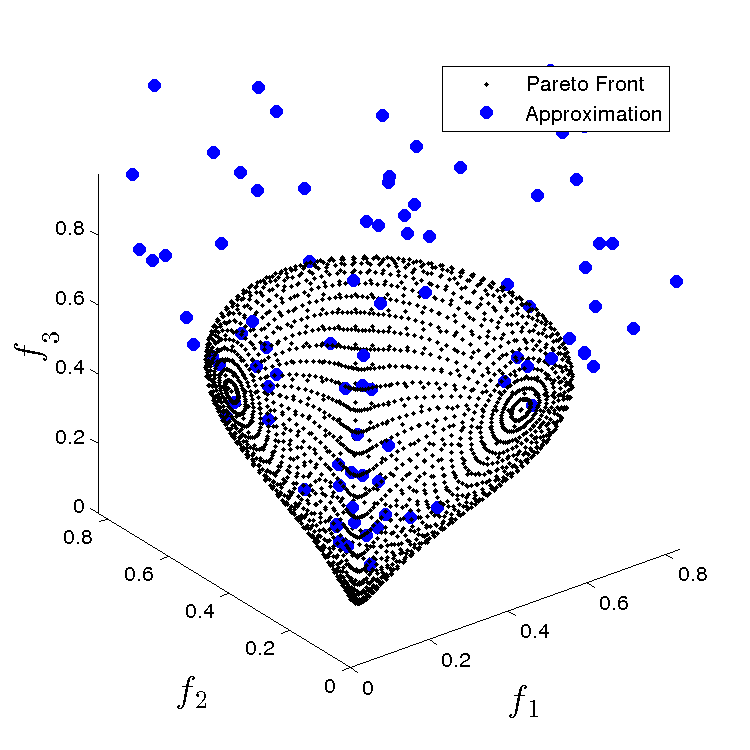

Supplement: Multimedia component 1 [file mmc1.zip › DataInBrief/Images/pf_HV_GDE3_H1_DTLZ2_k4.png]

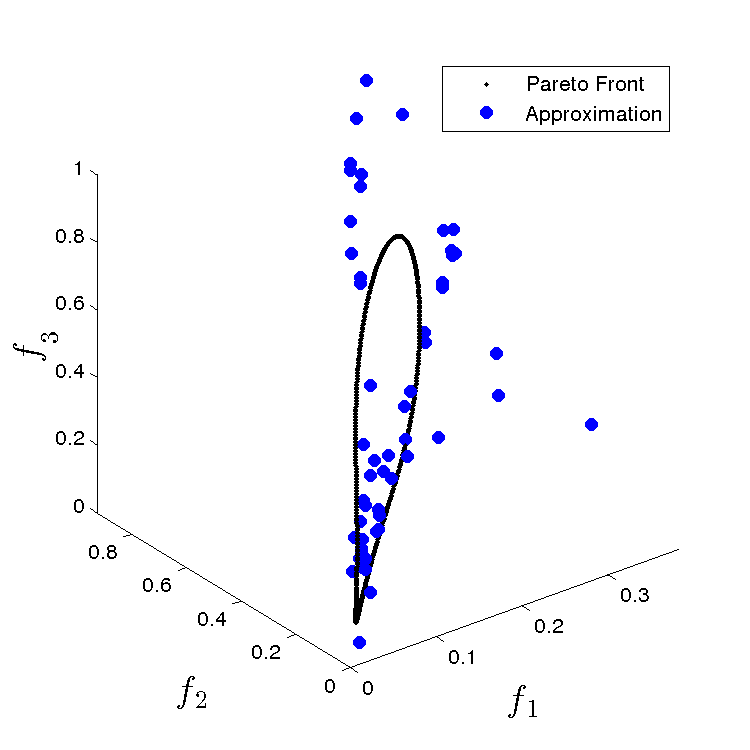

Supplement: Multimedia component 1 [file mmc1.zip › DataInBrief/Images/pf_HV_GDE3_H2_DTLZ2_k4.png]

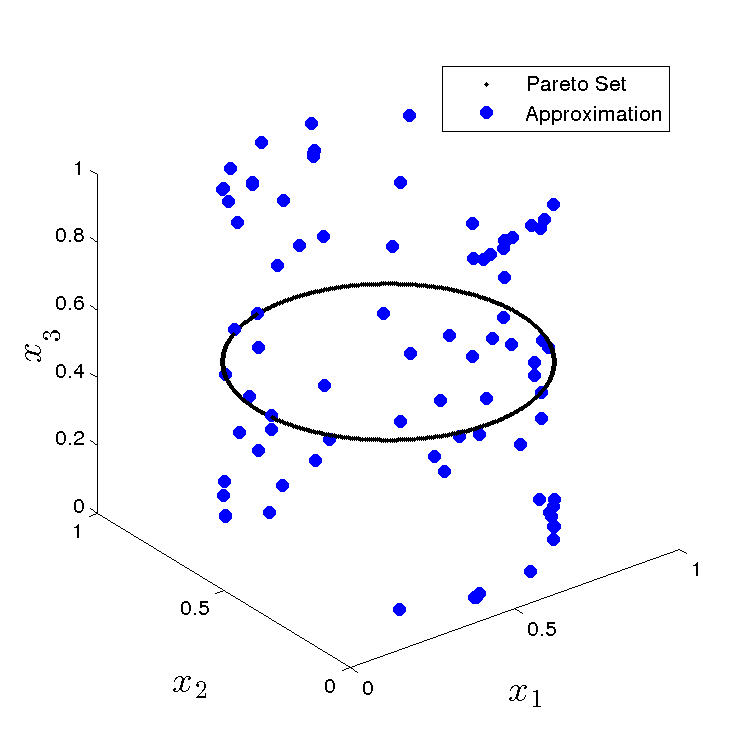

Supplement: Multimedia component 1 [file mmc1.zip › DataInBrief/Images/ps_DP_GDE3_H1_DTLZ1_k3.png]

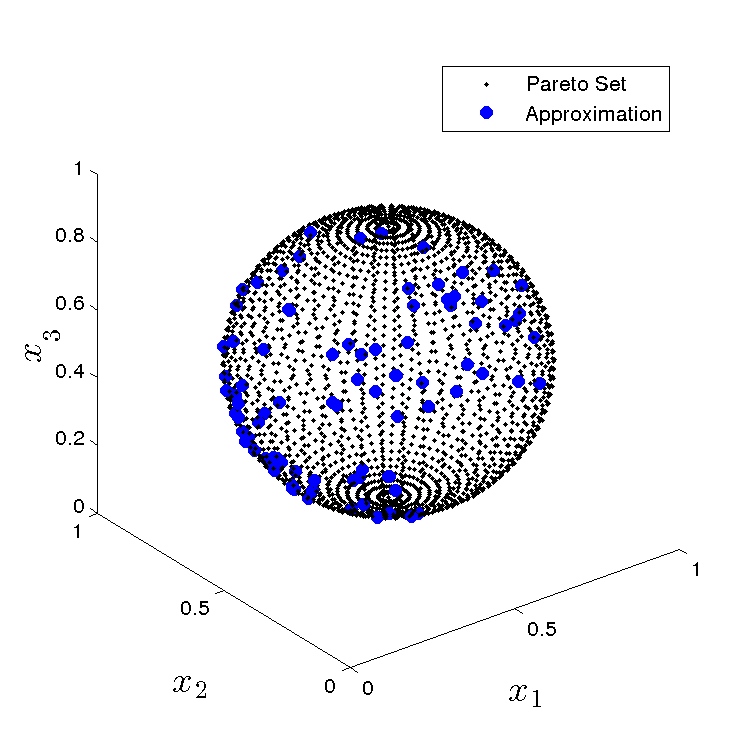

Supplement: Multimedia component 1 [file mmc1.zip › DataInBrief/Images/ps_DP_GDE3_H1_DTLZ2_k4.png]

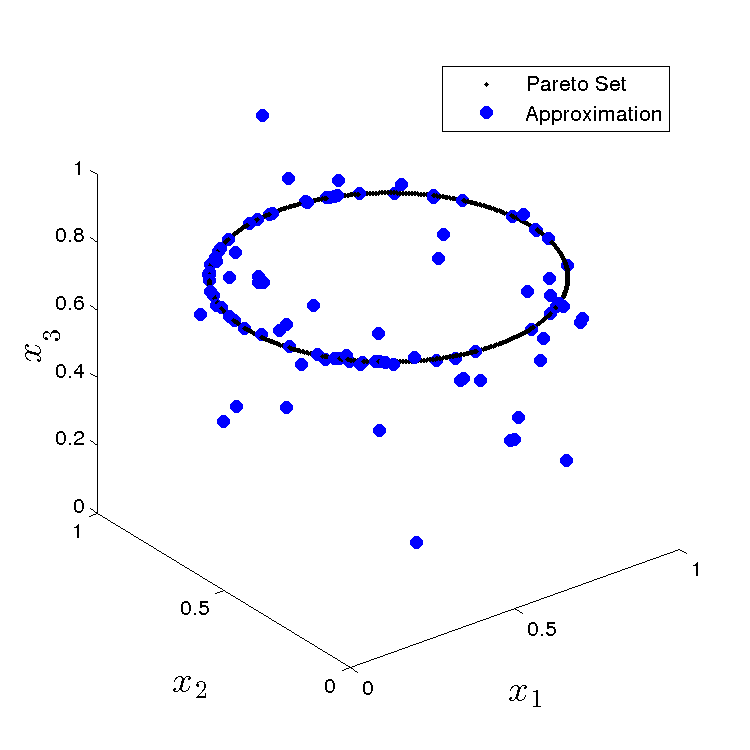

Supplement: Multimedia component 1 [file mmc1.zip › DataInBrief/Images/ps_DP_GDE3_H2_DTLZ2_k4.png]

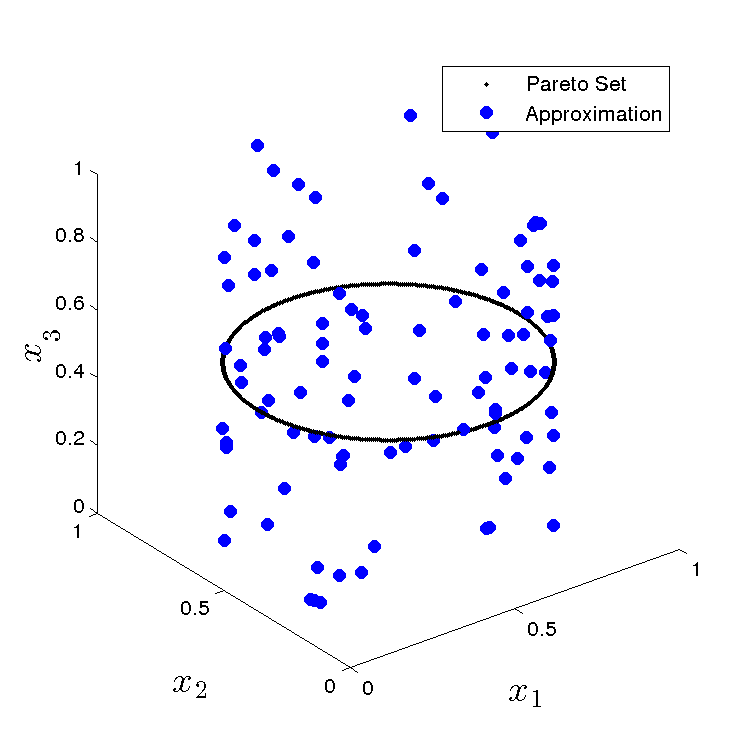

Supplement: Multimedia component 1 [file mmc1.zip › DataInBrief/Images/ps_HV_GDE3_H1_DTLZ1_k3.png]

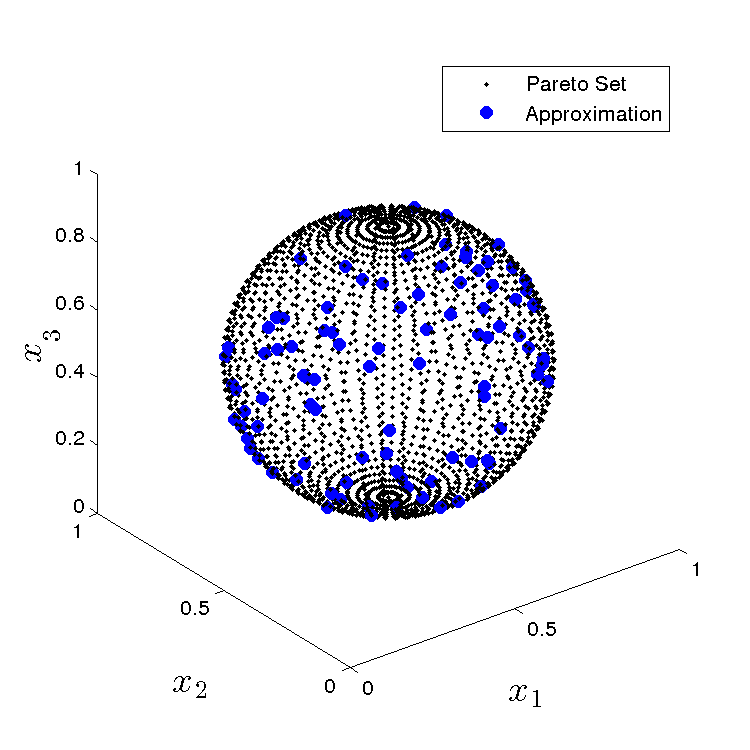

Supplement: Multimedia component 1 [file mmc1.zip › DataInBrief/Images/ps_HV_GDE3_H1_DTLZ2_k4.png]

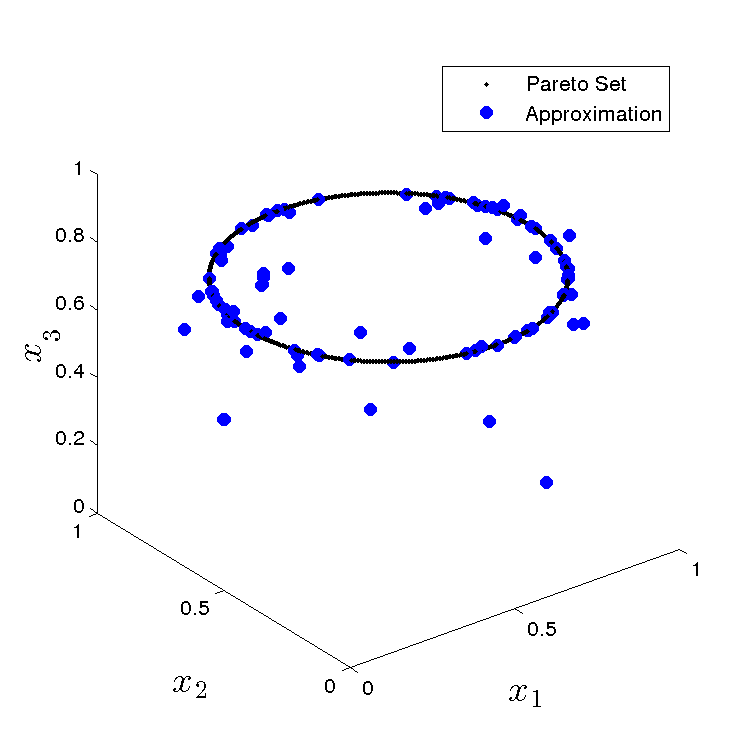

Supplement: Multimedia component 1 [file mmc1.zip › DataInBrief/Images/ps_HV_GDE3_H2_DTLZ2_k4.png]

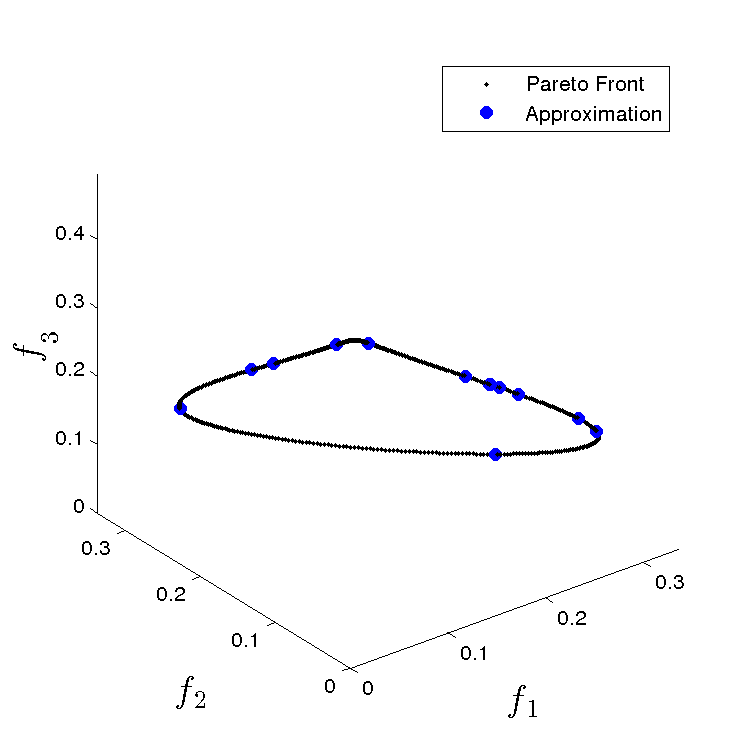

Supplement: Multimedia component 1 [file mmc1.zip › DataInBrief/Images/pf_DP_MOEADD_H1_DTLZ1_k3.png]

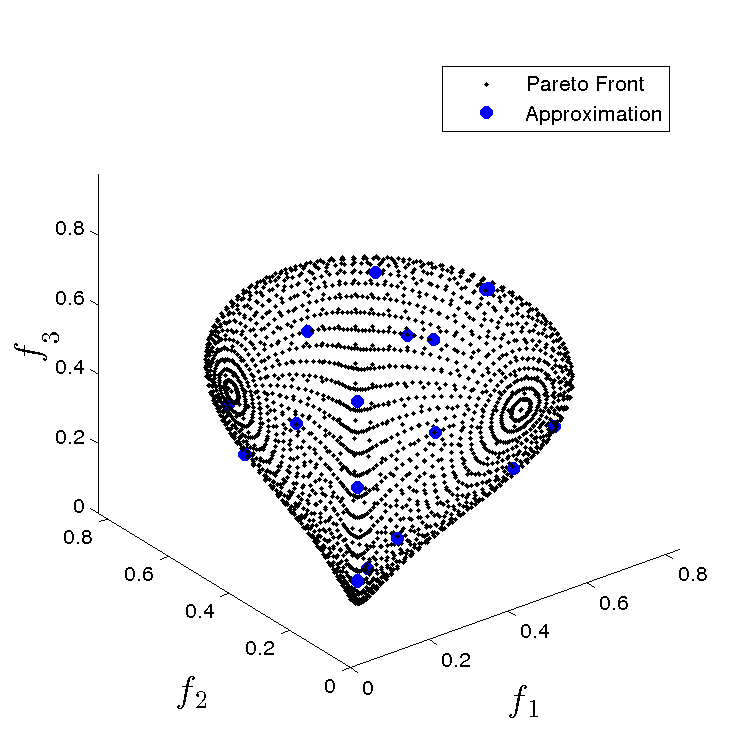

Supplement: Multimedia component 1 [file mmc1.zip › DataInBrief/Images/pf_DP_MOEADD_H1_DTLZ2_k4.png]

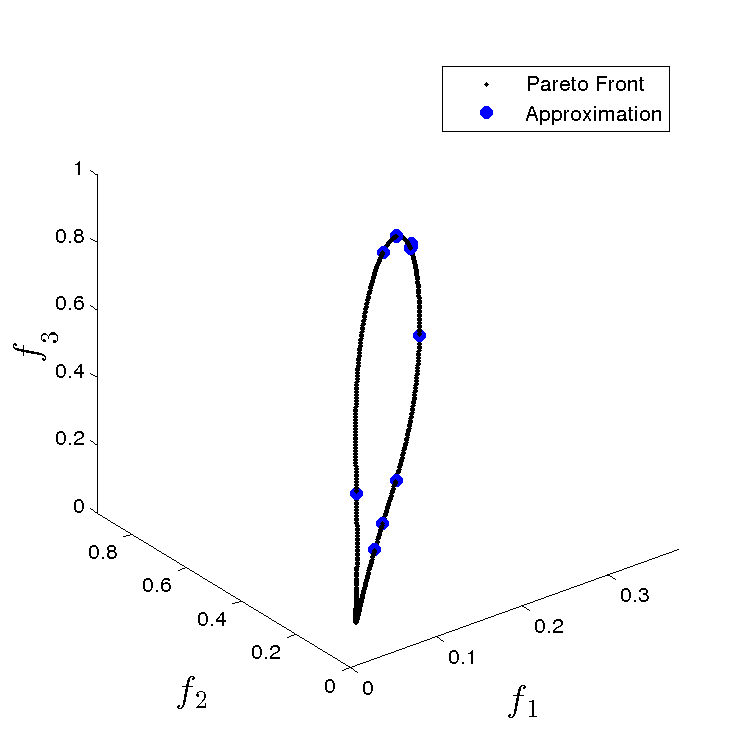

Supplement: Multimedia component 1 [file mmc1.zip › DataInBrief/Images/pf_DP_MOEADD_H2_DTLZ2_k4.png]

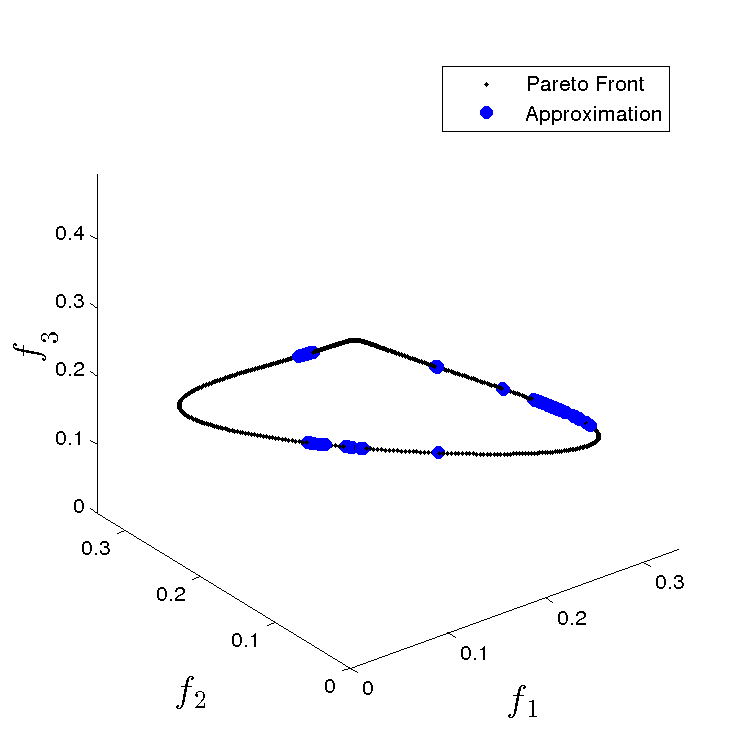

Supplement: Multimedia component 1 [file mmc1.zip › DataInBrief/Images/pf_DP_NSGAII_H1_DTLZ1_k3.png]

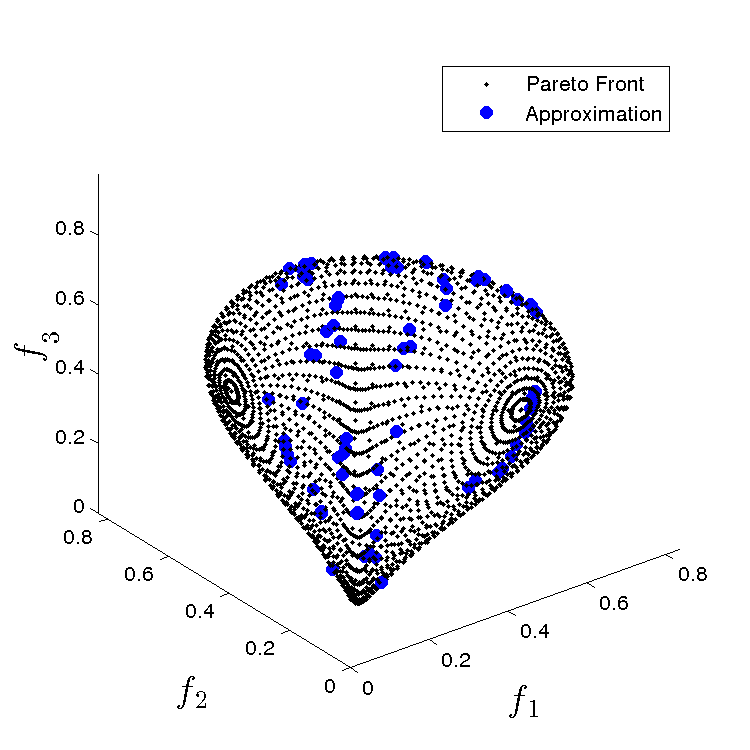

Supplement: Multimedia component 1 [file mmc1.zip › DataInBrief/Images/pf_DP_NSGAII_H1_DTLZ2_k4.png]

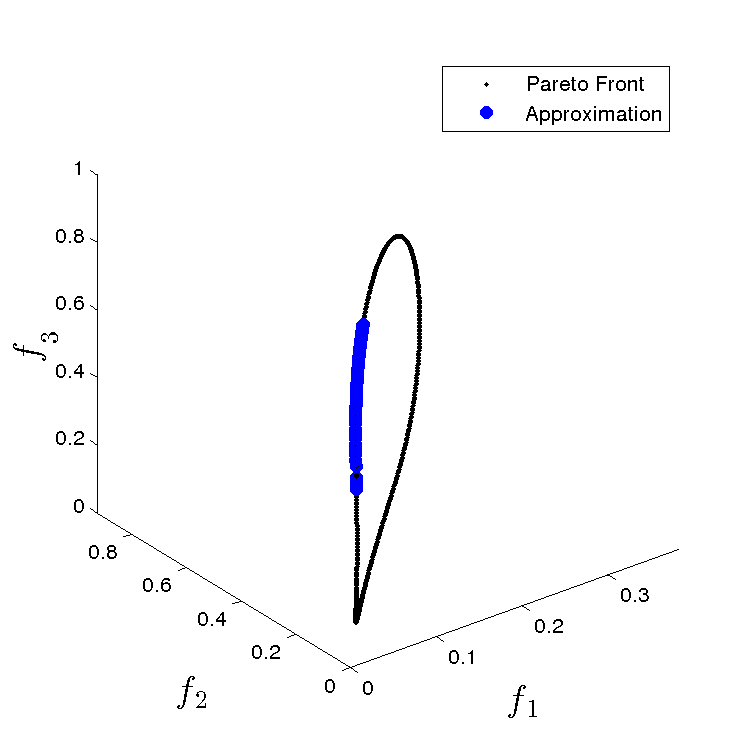

Supplement: Multimedia component 1 [file mmc1.zip › DataInBrief/Images/pf_DP_NSGAII_H2_DTLZ2_k4.png]

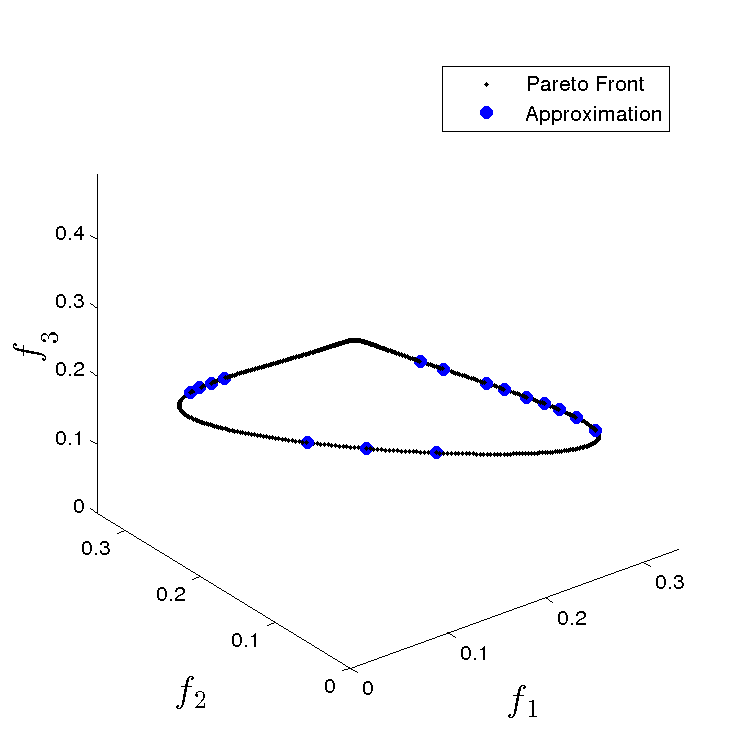

Supplement: Multimedia component 1 [file mmc1.zip › DataInBrief/Images/pf_HV_MOEADD_H1_DTLZ1_k3.png]

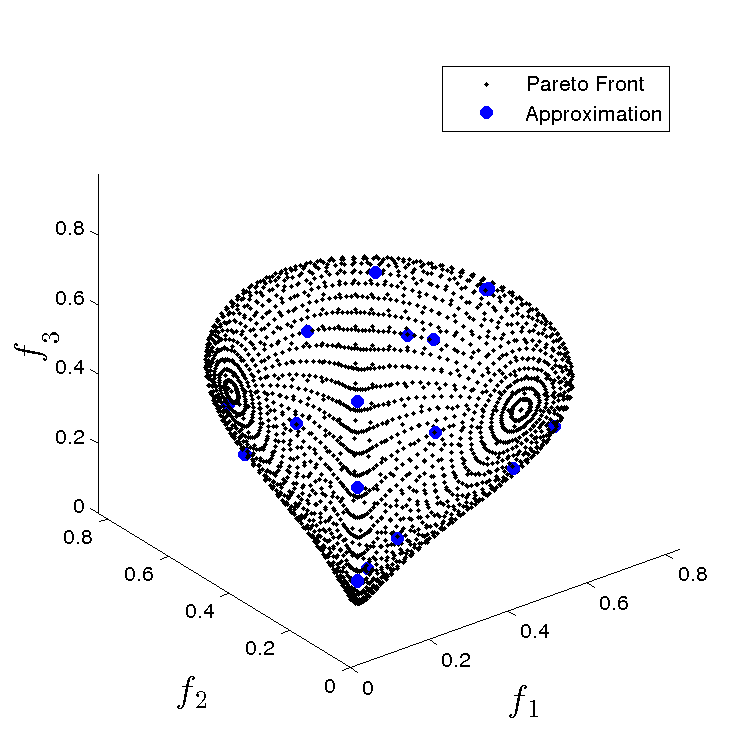

Supplement: Multimedia component 1 [file mmc1.zip › DataInBrief/Images/pf_HV_MOEADD_H1_DTLZ2_k4.png]

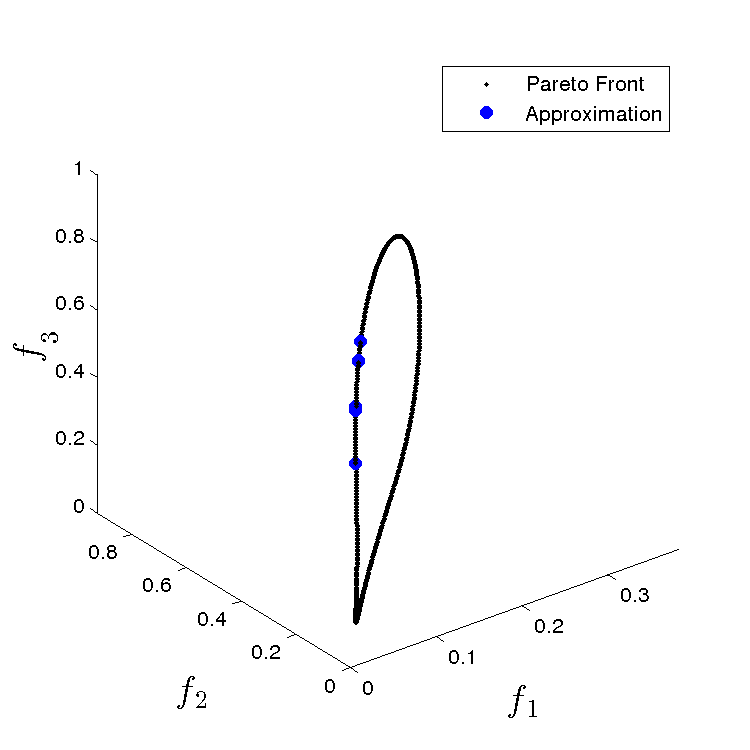

Supplement: Multimedia component 1 [file mmc1.zip › DataInBrief/Images/pf_HV_MOEADD_H2_DTLZ2_k4.png]

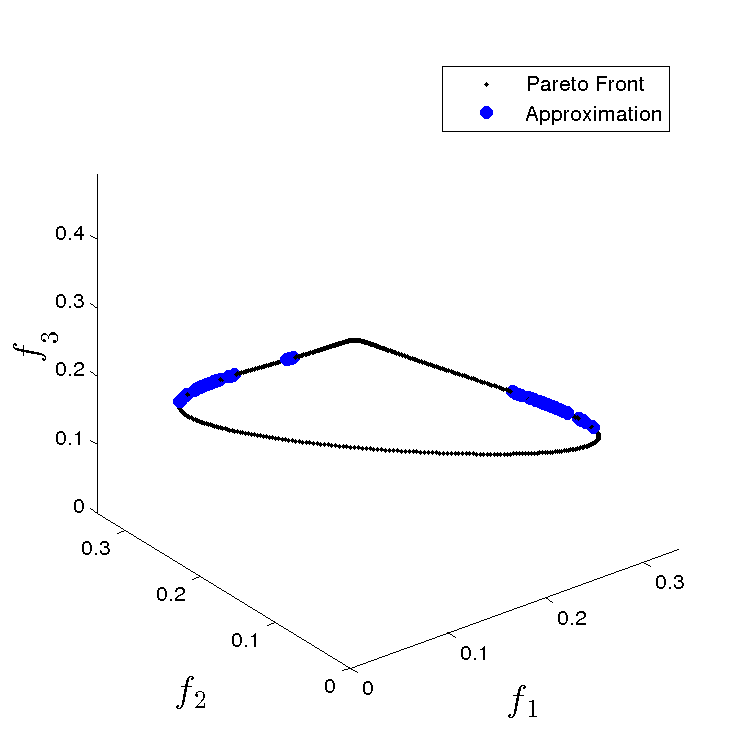

Supplement: Multimedia component 1 [file mmc1.zip › DataInBrief/Images/pf_HV_NSGAII_H1_DTLZ1_k3.png]

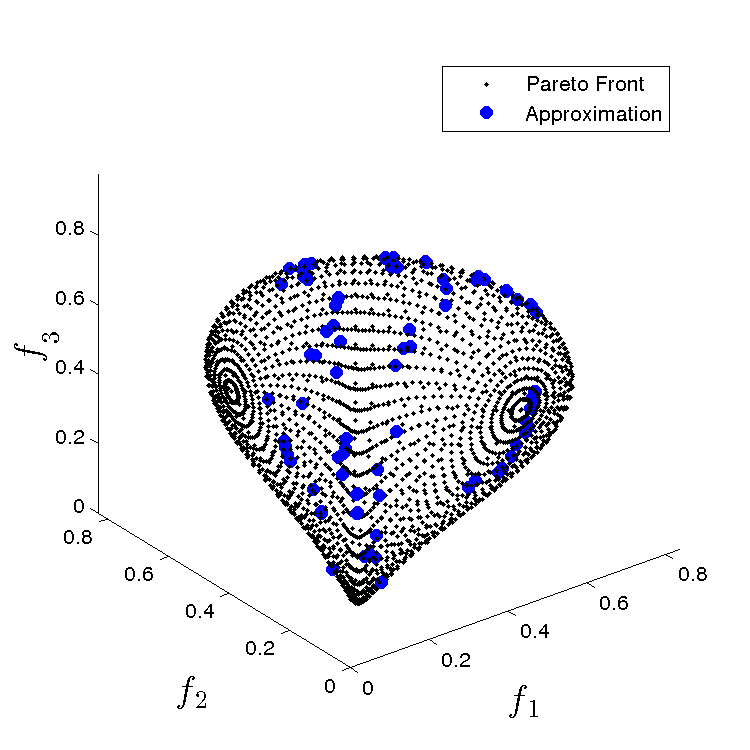

Supplement: Multimedia component 1 [file mmc1.zip › DataInBrief/Images/pf_HV_NSGAII_H1_DTLZ2_k4.png]

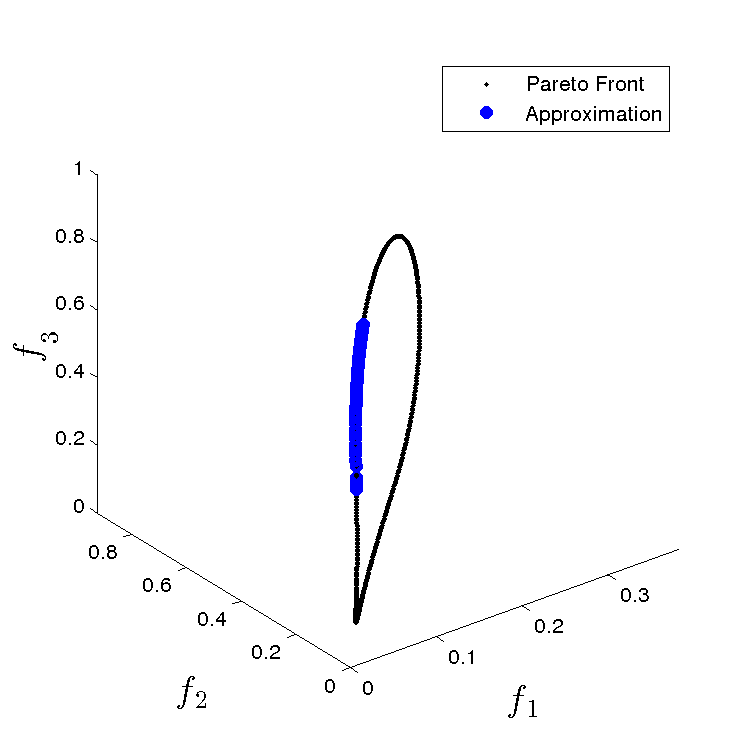

Supplement: Multimedia component 1 [file mmc1.zip › DataInBrief/Images/pf_HV_NSGAII_H2_DTLZ2_k4.png]

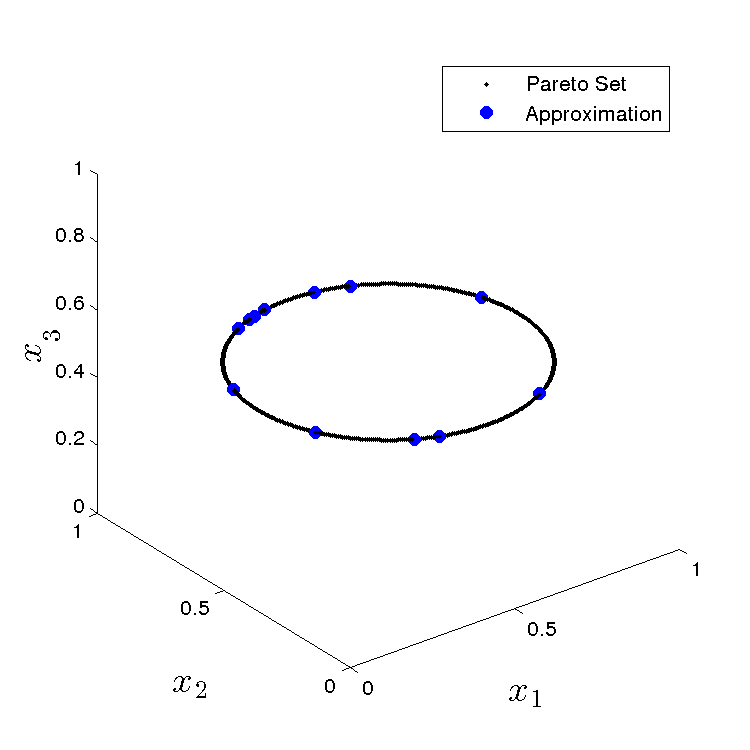

Supplement: Multimedia component 1 [file mmc1.zip › DataInBrief/Images/ps_DP_MOEADD_H1_DTLZ1_k3.png]

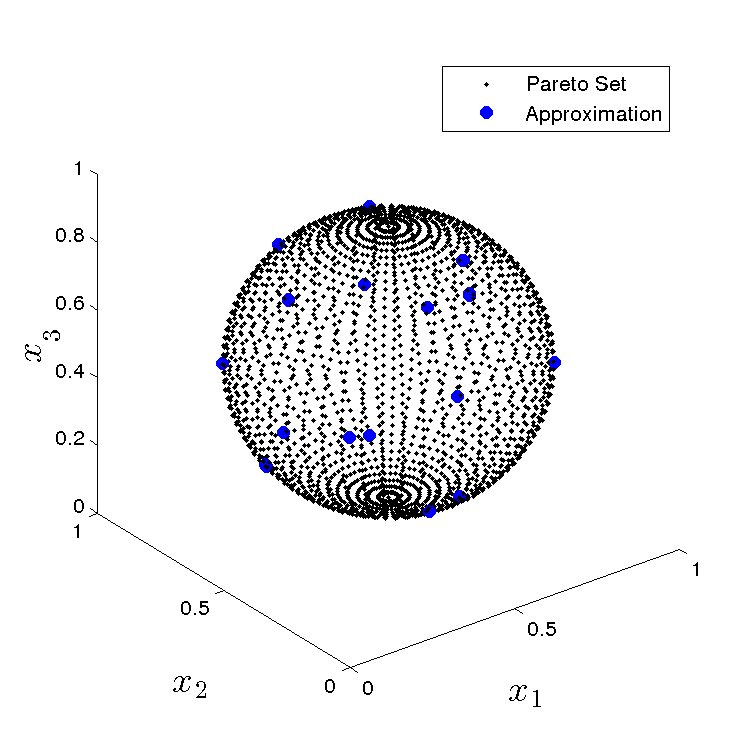

Supplement: Multimedia component 1 [file mmc1.zip › DataInBrief/Images/ps_DP_MOEADD_H1_DTLZ2_k4.png]

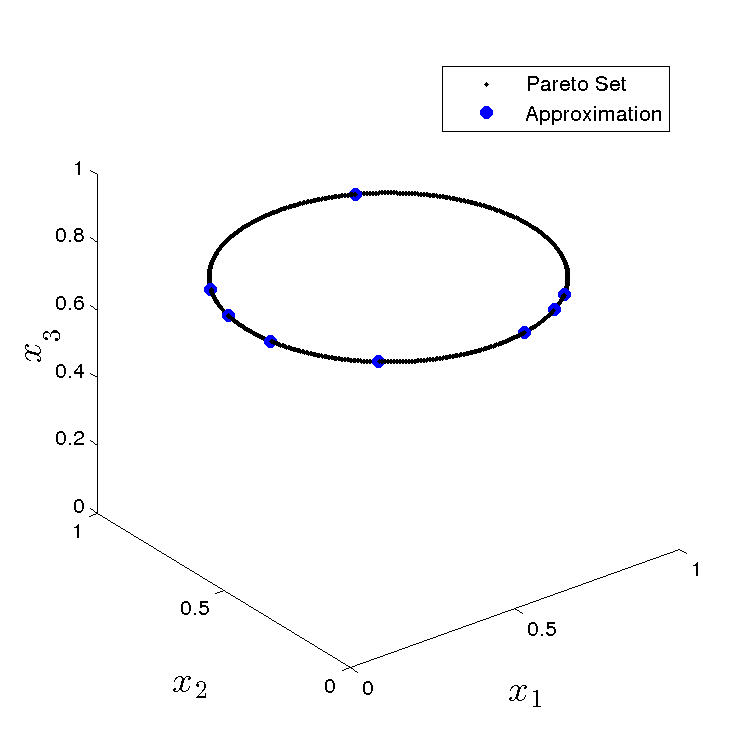

Supplement: Multimedia component 1 [file mmc1.zip › DataInBrief/Images/ps_DP_MOEADD_H2_DTLZ2_k4.png]

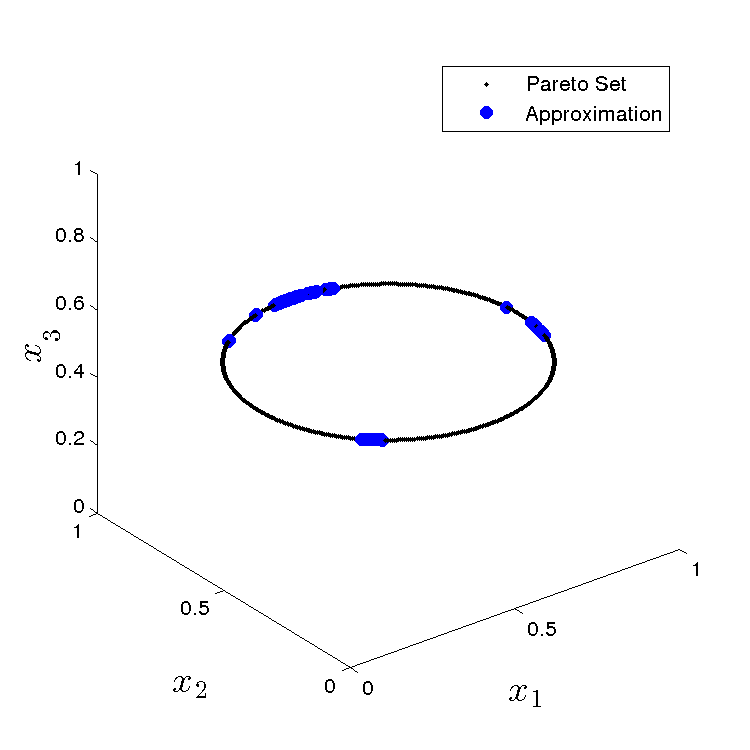

Supplement: Multimedia component 1 [file mmc1.zip › DataInBrief/Images/ps_DP_NSGAII_H1_DTLZ1_k3.png]

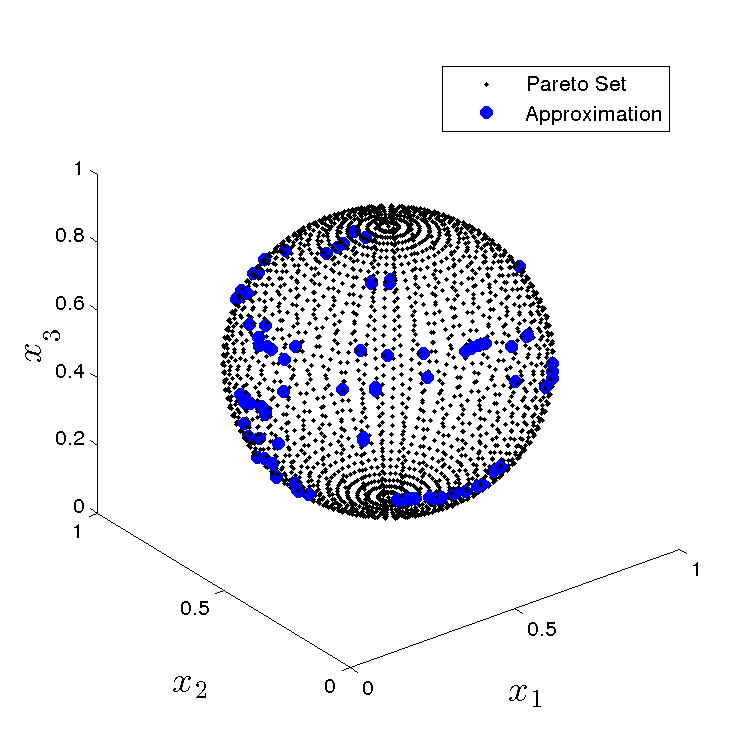

Supplement: Multimedia component 1 [file mmc1.zip › DataInBrief/Images/ps_DP_NSGAII_H1_DTLZ2_k4.png]

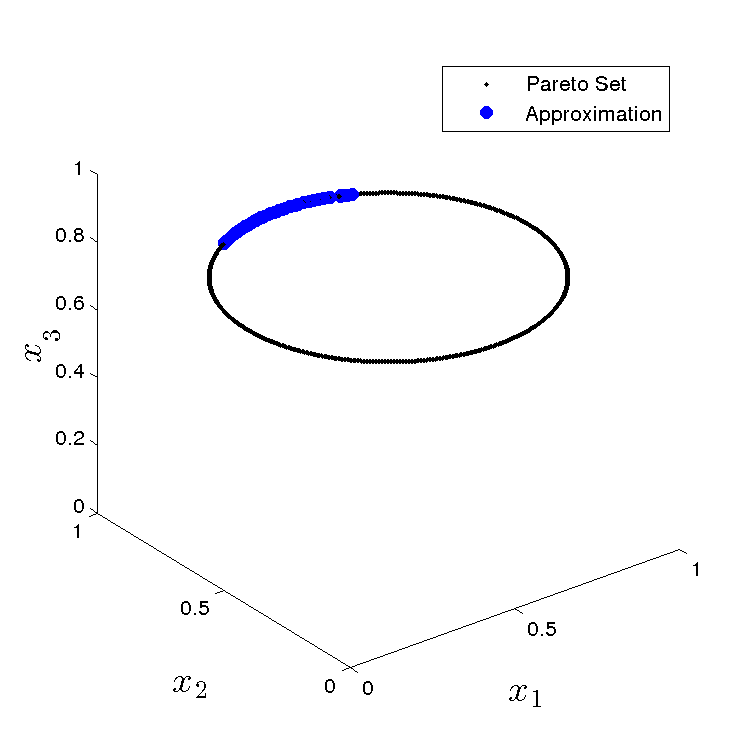

Supplement: Multimedia component 1 [file mmc1.zip › DataInBrief/Images/ps_DP_NSGAII_H2_DTLZ2_k4.png]

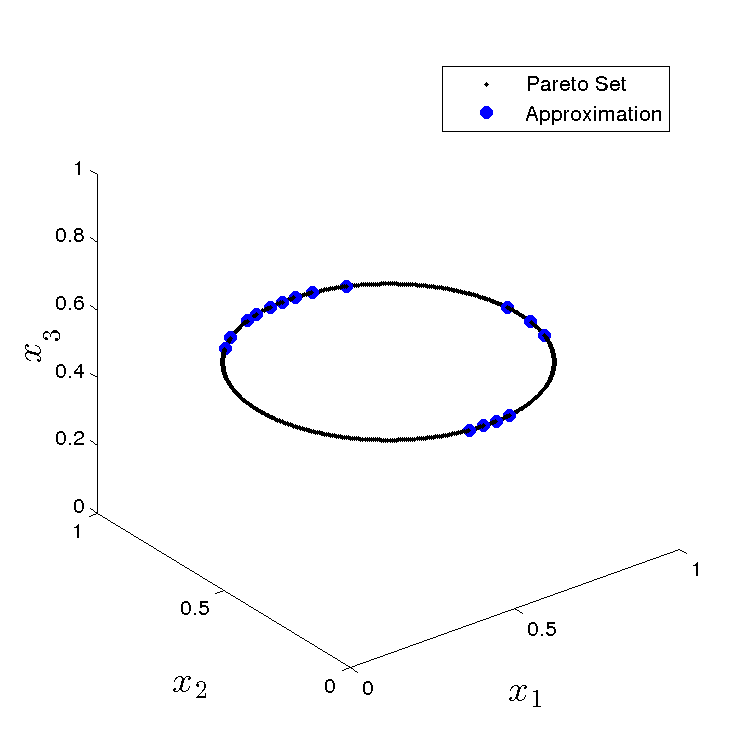

Supplement: Multimedia component 1 [file mmc1.zip › DataInBrief/Images/ps_HV_MOEADD_H1_DTLZ1_k3.png]

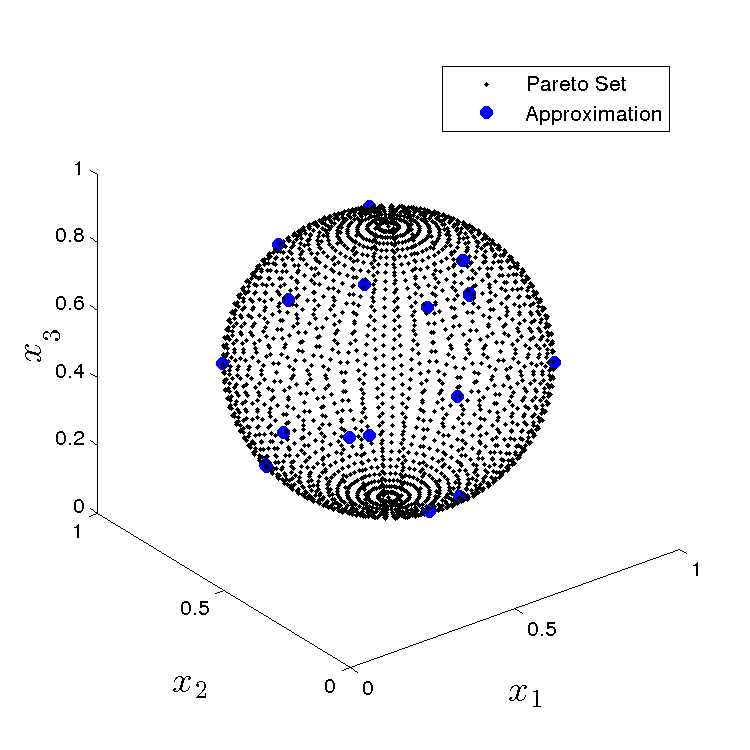

Supplement: Multimedia component 1 [file mmc1.zip › DataInBrief/Images/ps_HV_MOEADD_H1_DTLZ2_k4.png]

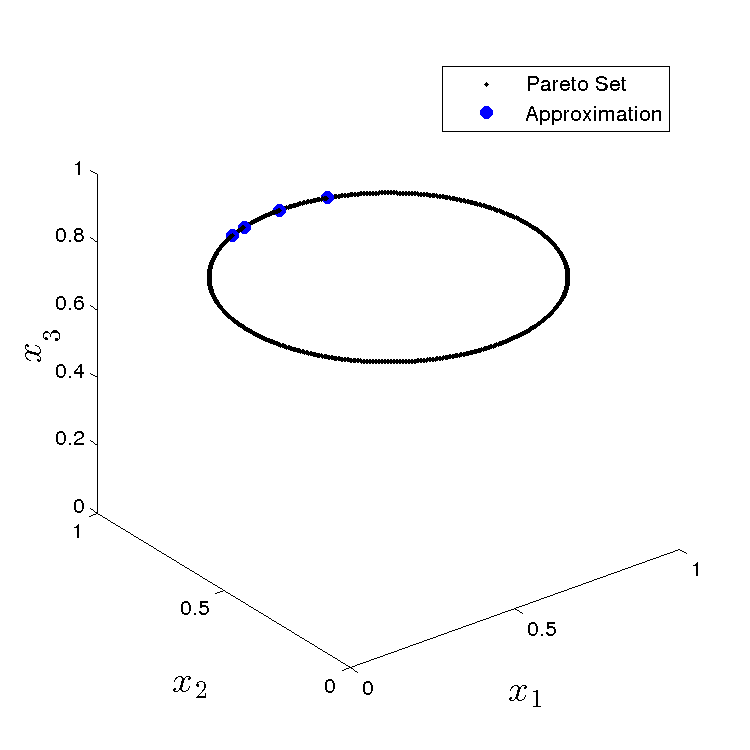

Supplement: Multimedia component 1 [file mmc1.zip › DataInBrief/Images/ps_HV_MOEADD_H2_DTLZ2_k4.png]

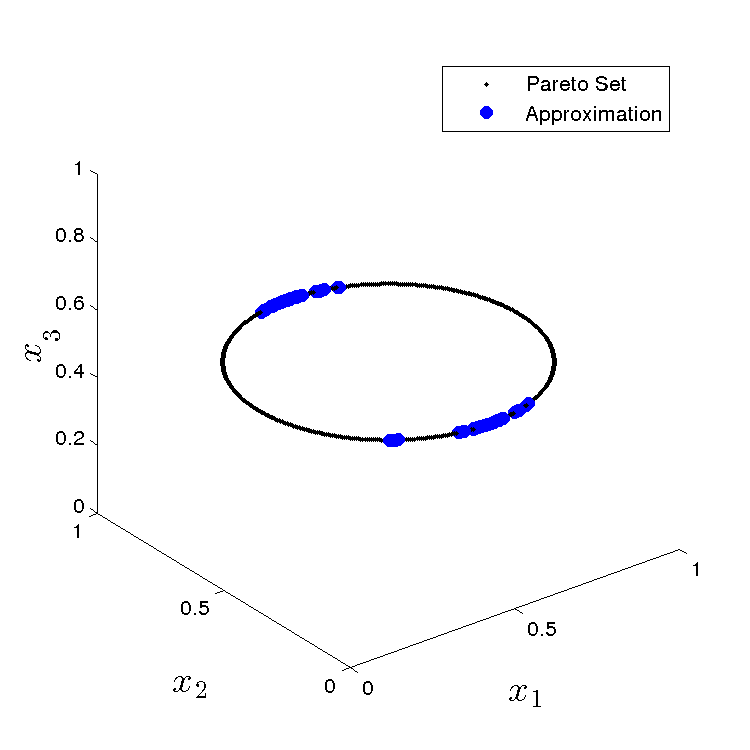

Supplement: Multimedia component 1 [file mmc1.zip › DataInBrief/Images/ps_HV_NSGAII_H1_DTLZ1_k3.png]

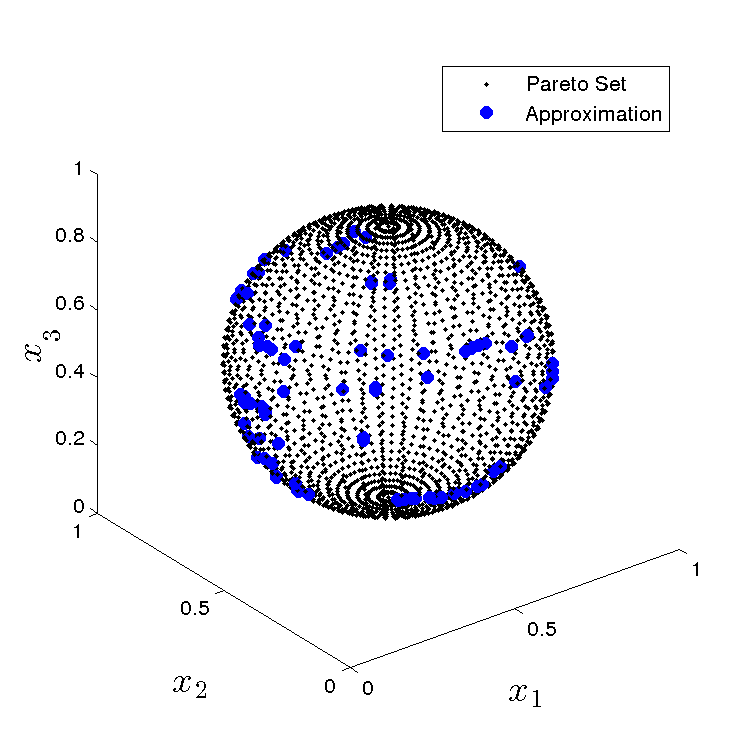

Supplement: Multimedia component 1 [file mmc1.zip › DataInBrief/Images/ps_HV_NSGAII_H1_DTLZ2_k4.png]

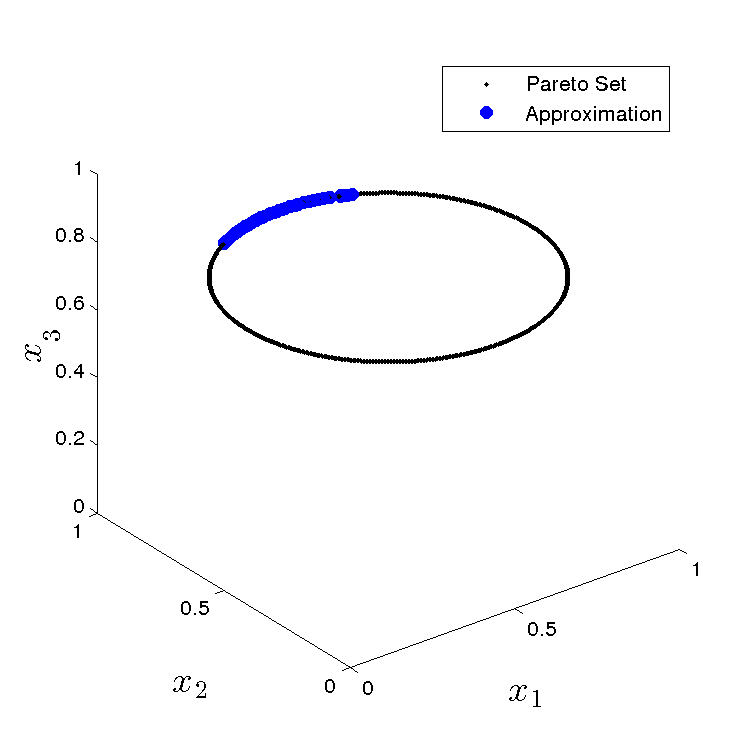

Supplement: Multimedia component 1 [file mmc1.zip › DataInBrief/Images/ps_HV_NSGAII_H2_DTLZ2_k4.png]

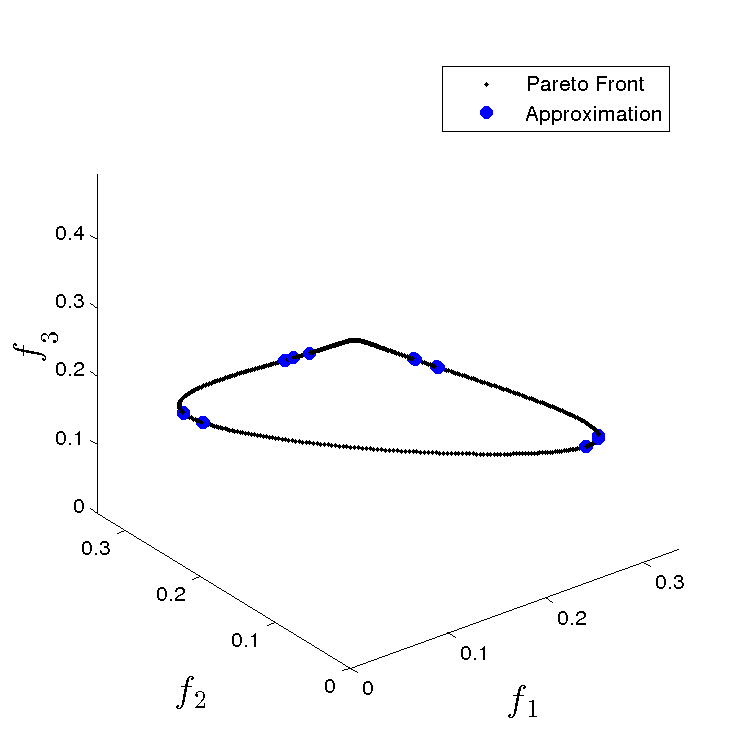

Supplement: Multimedia component 1 [file mmc1.zip › DataInBrief/Images/pf_DP_NSGAIII_H1_DTLZ1_k3.png]

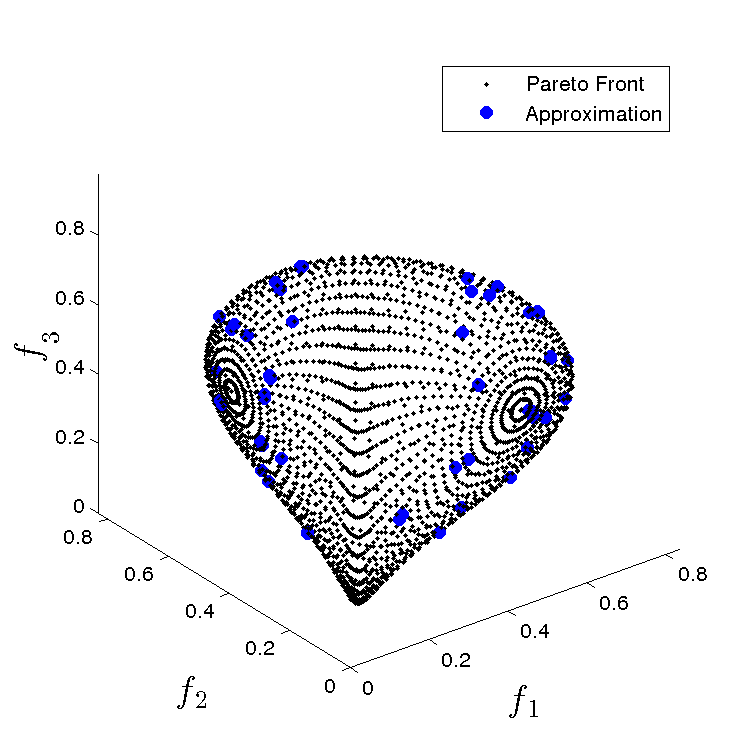

Supplement: Multimedia component 1 [file mmc1.zip › DataInBrief/Images/pf_DP_NSGAIII_H1_DTLZ2_k4.png]

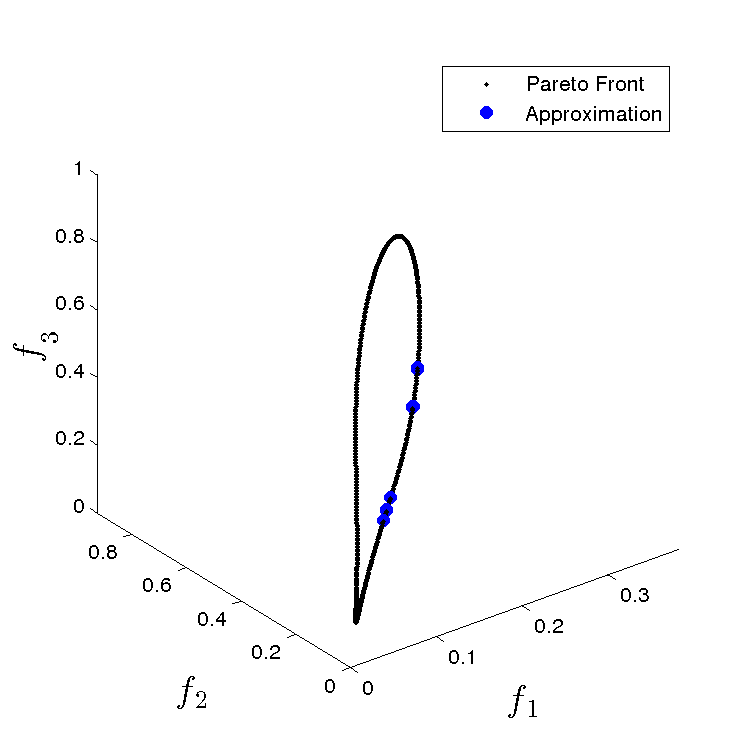

Supplement: Multimedia component 1 [file mmc1.zip › DataInBrief/Images/pf_DP_NSGAIII_H2_DTLZ2_k4.png]

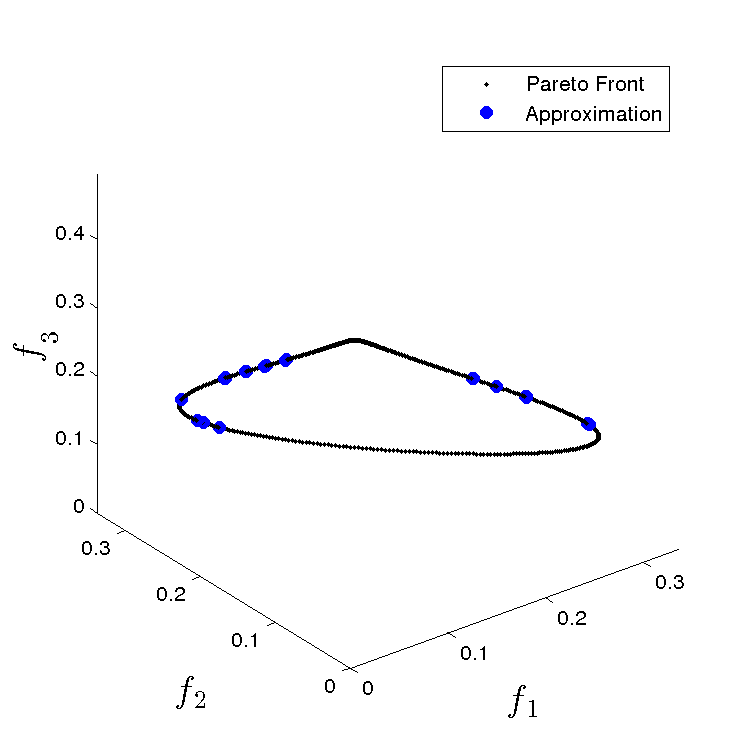

Supplement: Multimedia component 1 [file mmc1.zip › DataInBrief/Images/pf_HV_NSGAIII_H1_DTLZ1_k3.png]

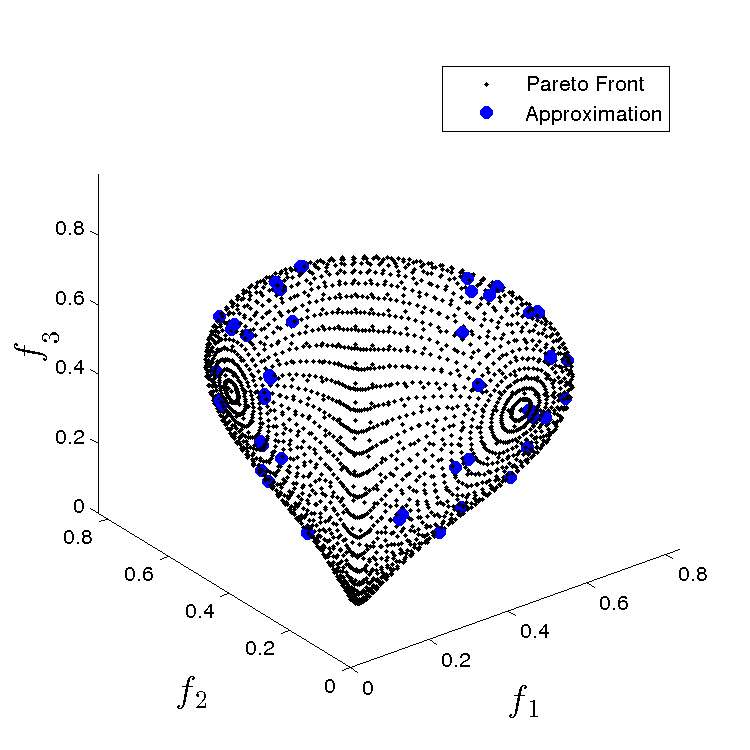

Supplement: Multimedia component 1 [file mmc1.zip › DataInBrief/Images/pf_HV_NSGAIII_H1_DTLZ2_k4.png]

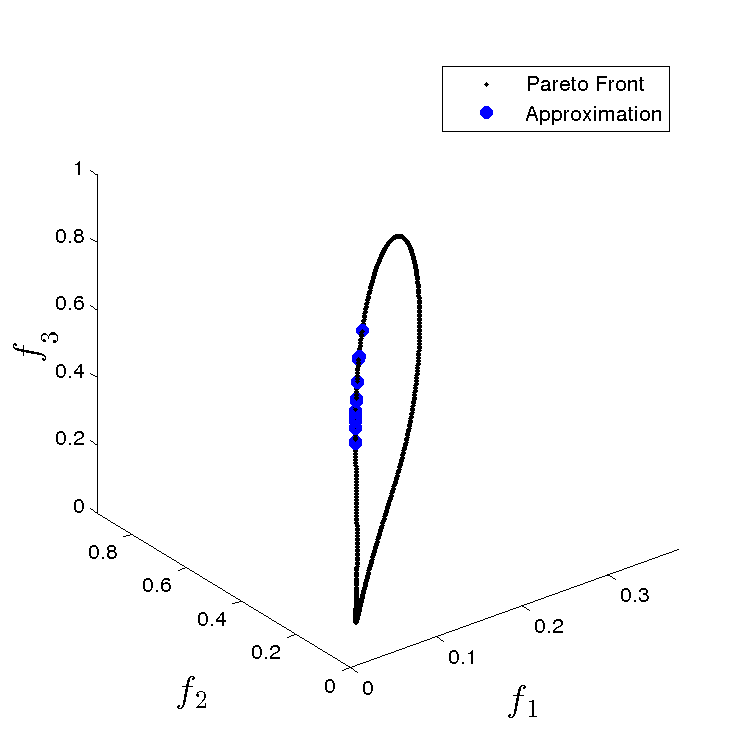

Supplement: Multimedia component 1 [file mmc1.zip › DataInBrief/Images/pf_HV_NSGAIII_H2_DTLZ2_k4.png]

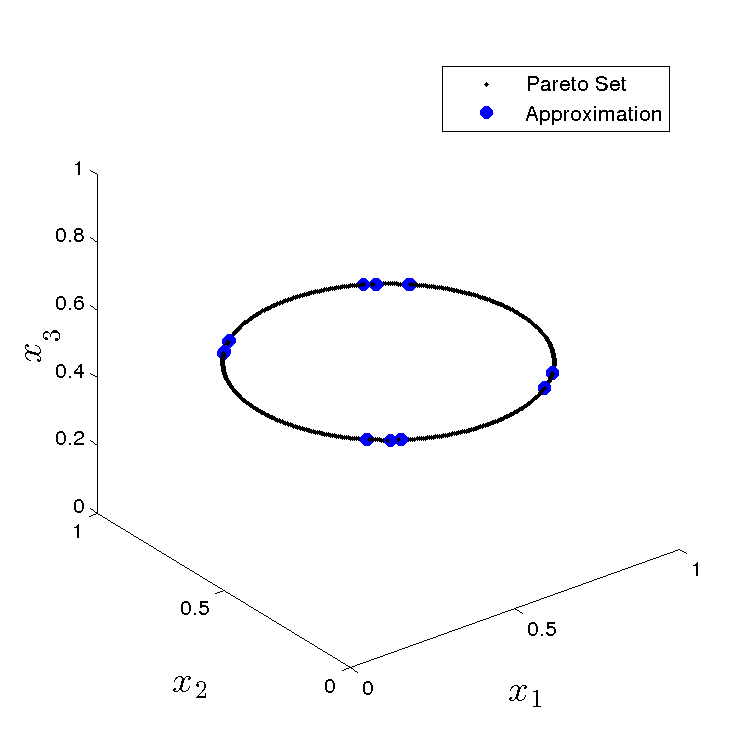

Supplement: Multimedia component 1 [file mmc1.zip › DataInBrief/Images/ps_DP_NSGAIII_H1_DTLZ1_k3.png]

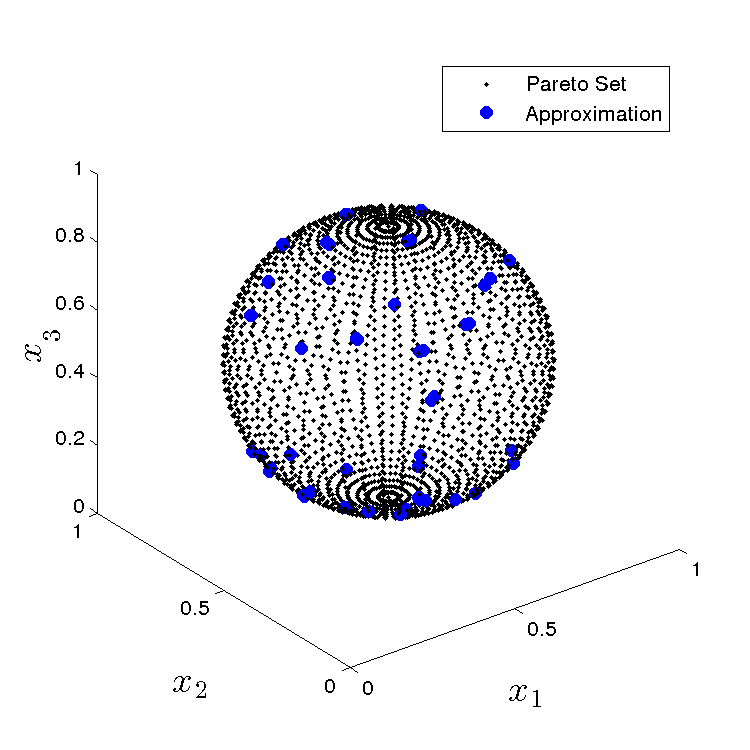

Supplement: Multimedia component 1 [file mmc1.zip › DataInBrief/Images/ps_DP_NSGAIII_H1_DTLZ2_k4.png]

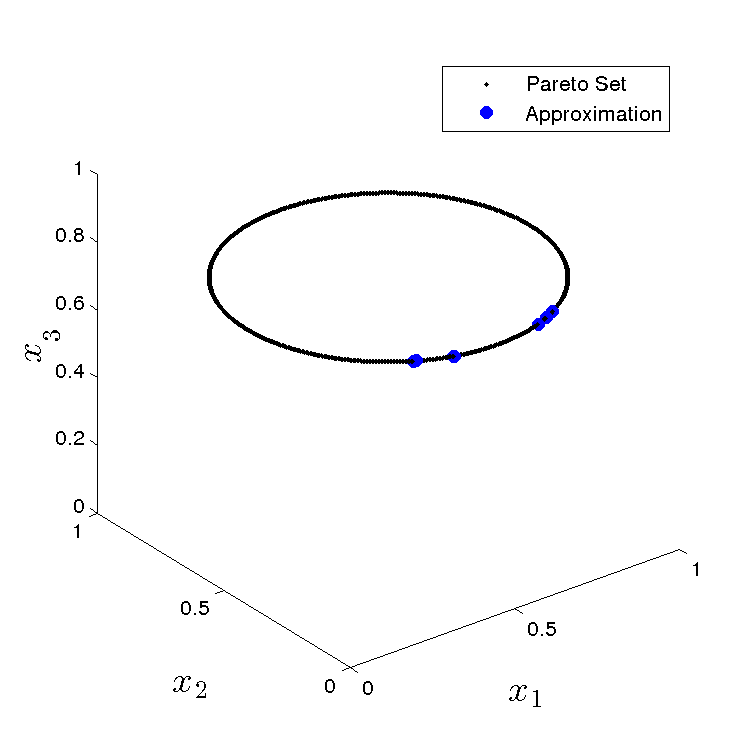

Supplement: Multimedia component 1 [file mmc1.zip › DataInBrief/Images/ps_DP_NSGAIII_H2_DTLZ2_k4.png]

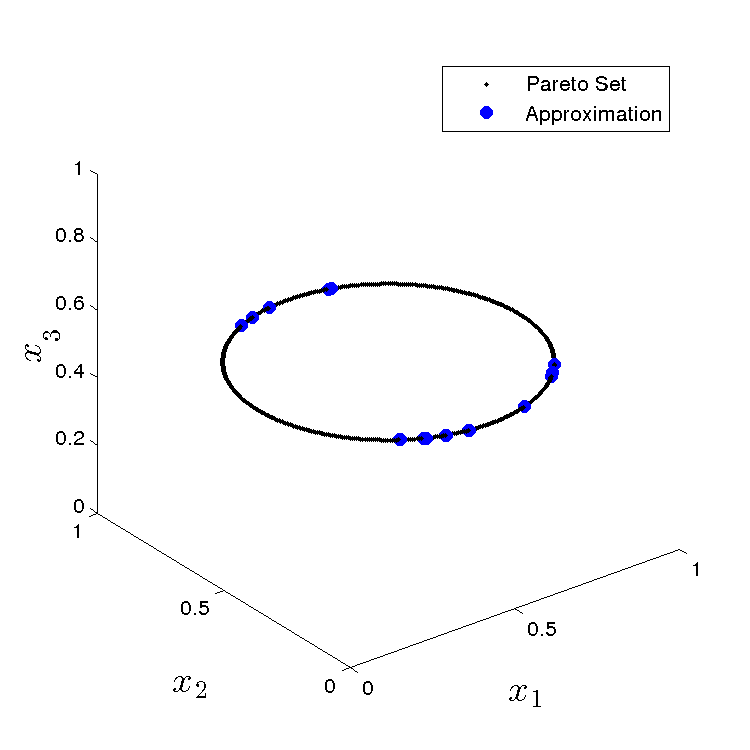

Supplement: Multimedia component 1 [file mmc1.zip › DataInBrief/Images/ps_HV_NSGAIII_H1_DTLZ1_k3.png]

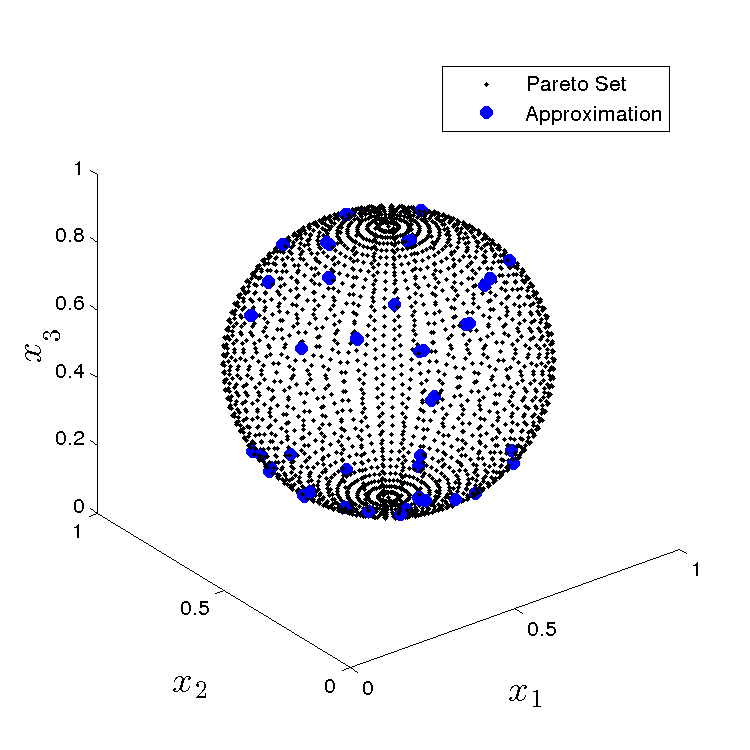

Supplement: Multimedia component 1 [file mmc1.zip › DataInBrief/Images/ps_HV_NSGAIII_H1_DTLZ2_k4.png]

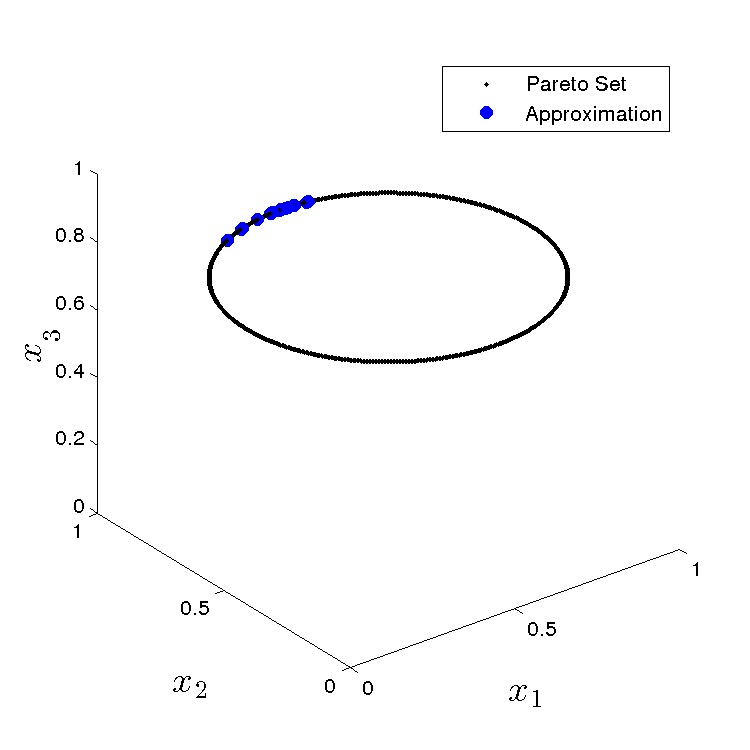

Supplement: Multimedia component 1 [file mmc1.zip › DataInBrief/Images/ps_HV_NSGAIII_H2_DTLZ2_k4.png]

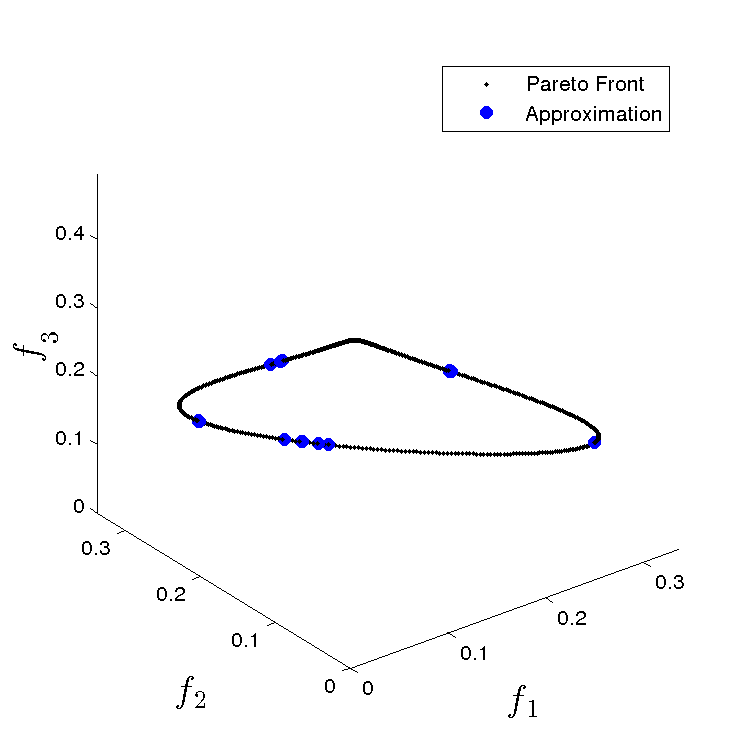

Supplement: Multimedia component 1 [file mmc1.zip › DataInBrief/Images/pf_DP_ANSGAIII_H1_DTLZ1_k3.png]

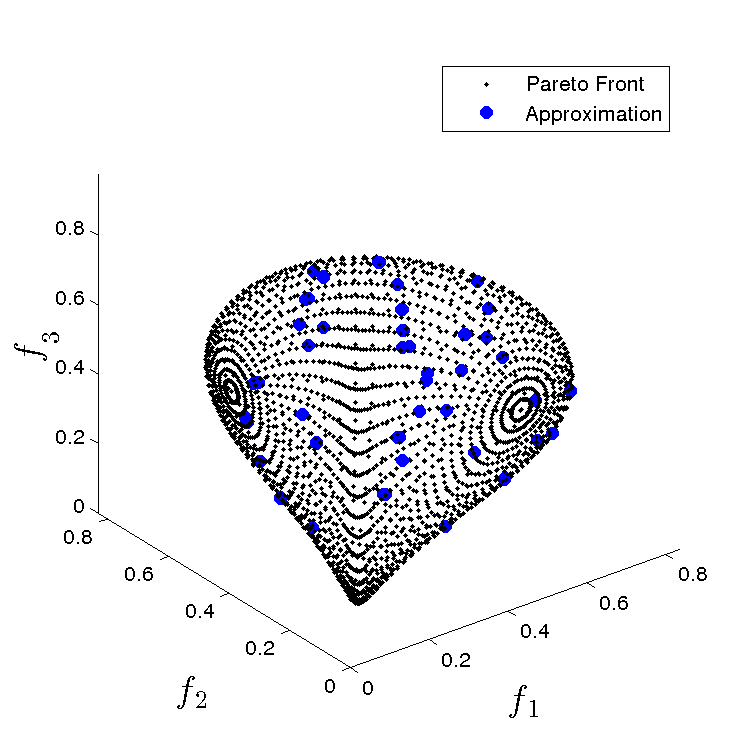

Supplement: Multimedia component 1 [file mmc1.zip › DataInBrief/Images/pf_DP_ANSGAIII_H1_DTLZ2_k4.png]

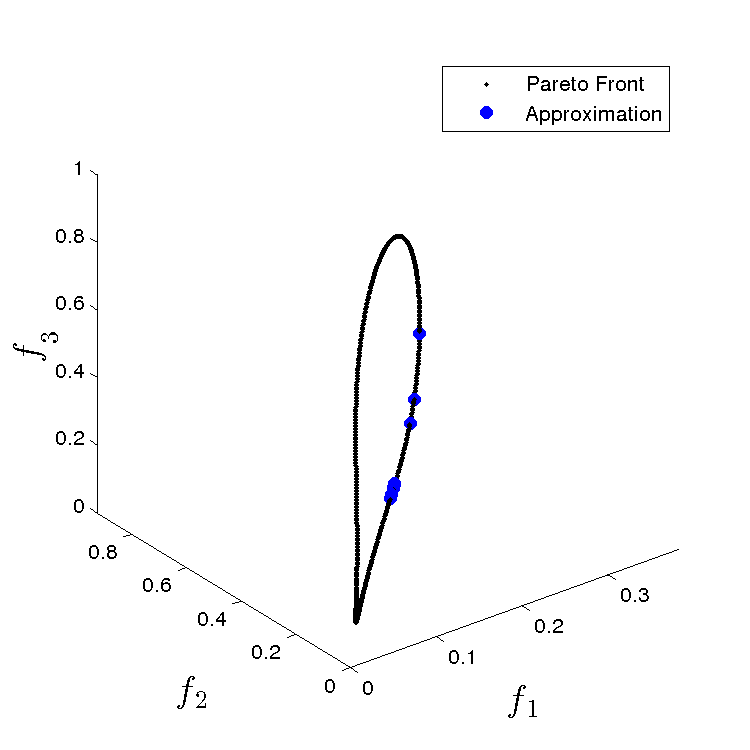

Supplement: Multimedia component 1 [file mmc1.zip › DataInBrief/Images/pf_DP_ANSGAIII_H2_DTLZ2_k4.png]

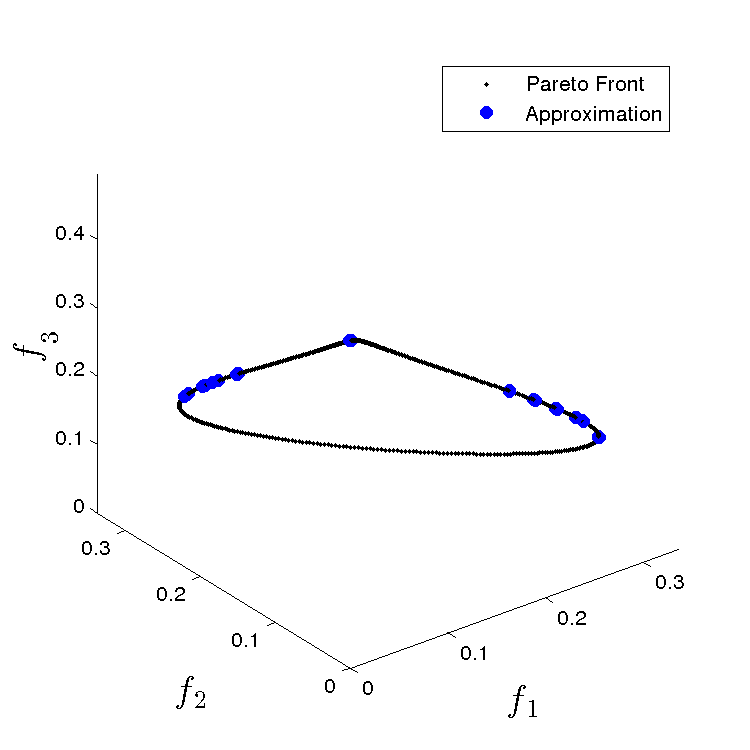

Supplement: Multimedia component 1 [file mmc1.zip › DataInBrief/Images/pf_HV_ANSGAIII_H1_DTLZ1_k3.png]

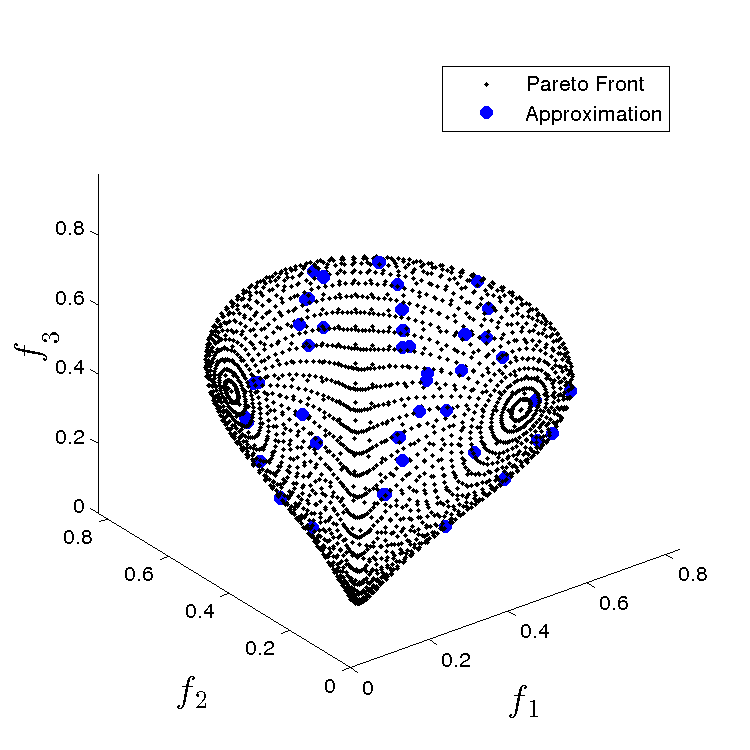

Supplement: Multimedia component 1 [file mmc1.zip › DataInBrief/Images/pf_HV_ANSGAIII_H1_DTLZ2_k4.png]

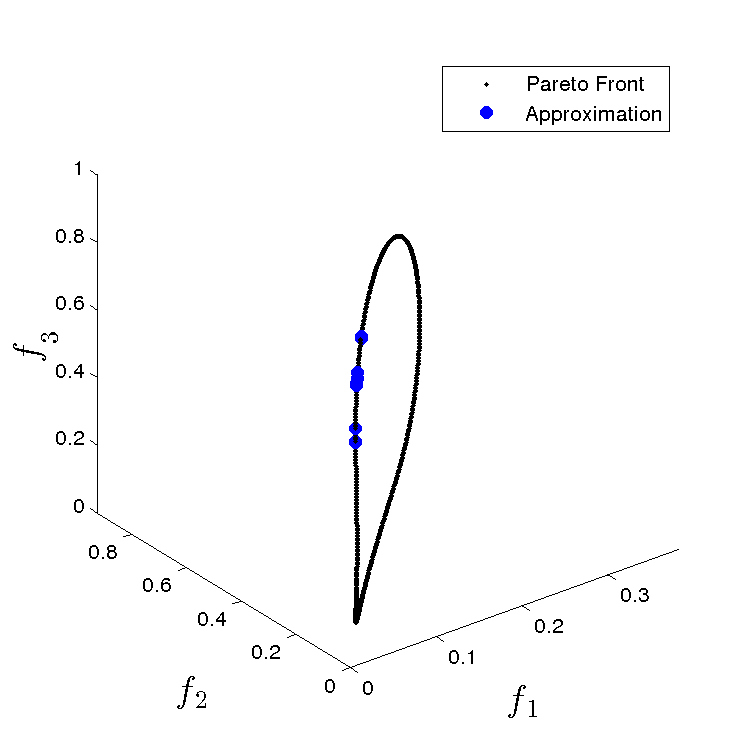

Supplement: Multimedia component 1 [file mmc1.zip › DataInBrief/Images/pf_HV_ANSGAIII_H2_DTLZ2_k4.png]

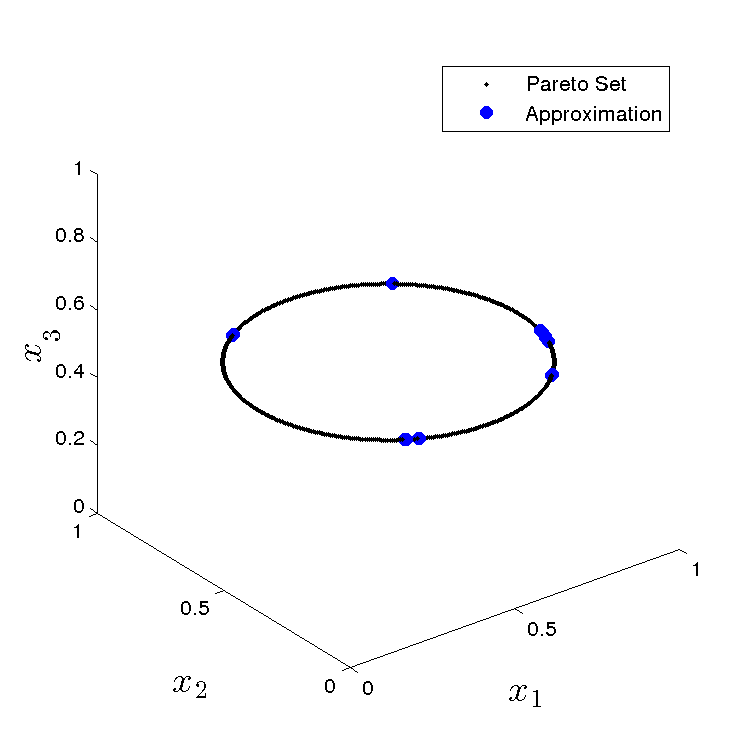

Supplement: Multimedia component 1 [file mmc1.zip › DataInBrief/Images/ps_DP_ANSGAIII_H1_DTLZ1_k3.png]

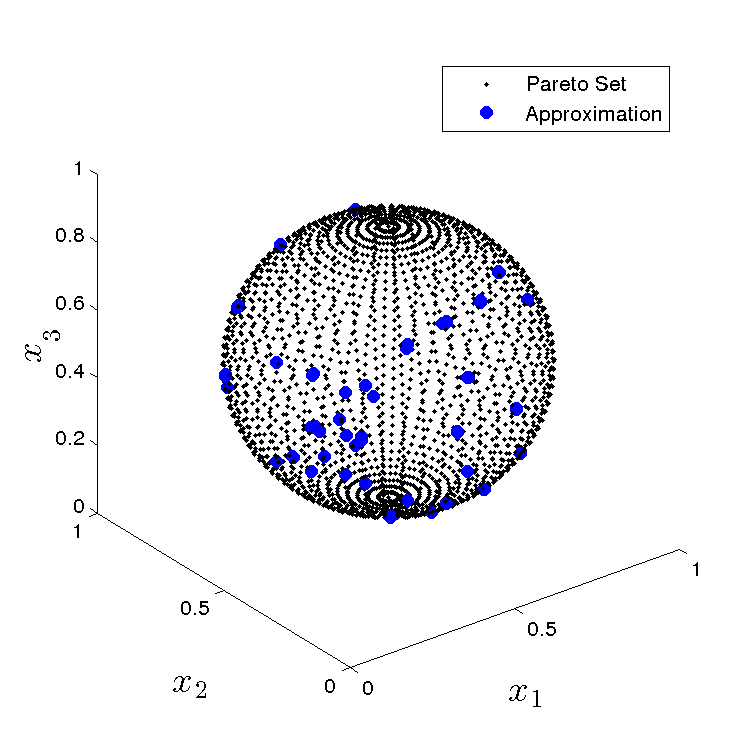

Supplement: Multimedia component 1 [file mmc1.zip › DataInBrief/Images/ps_DP_ANSGAIII_H1_DTLZ2_k4.png]

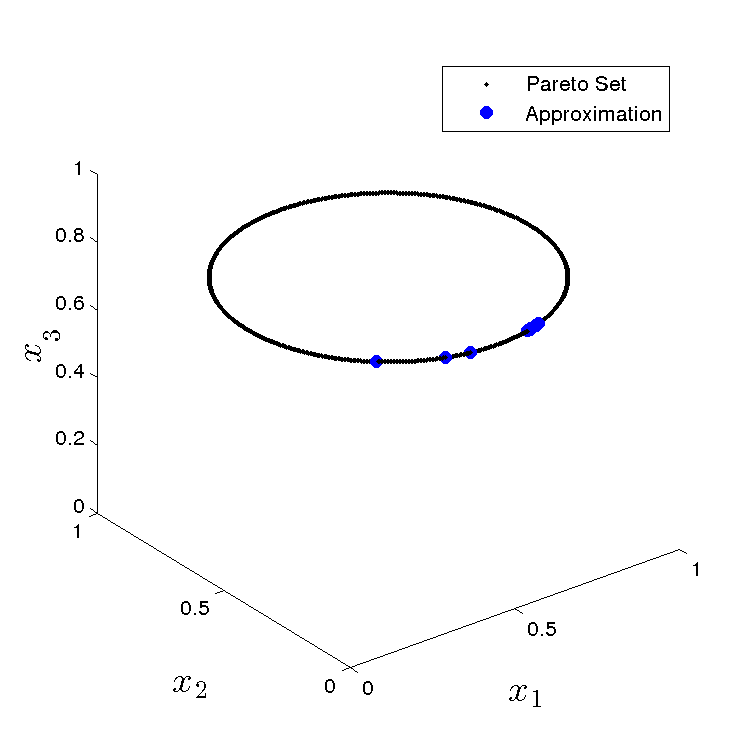

Supplement: Multimedia component 1 [file mmc1.zip › DataInBrief/Images/ps_DP_ANSGAIII_H2_DTLZ2_k4.png]

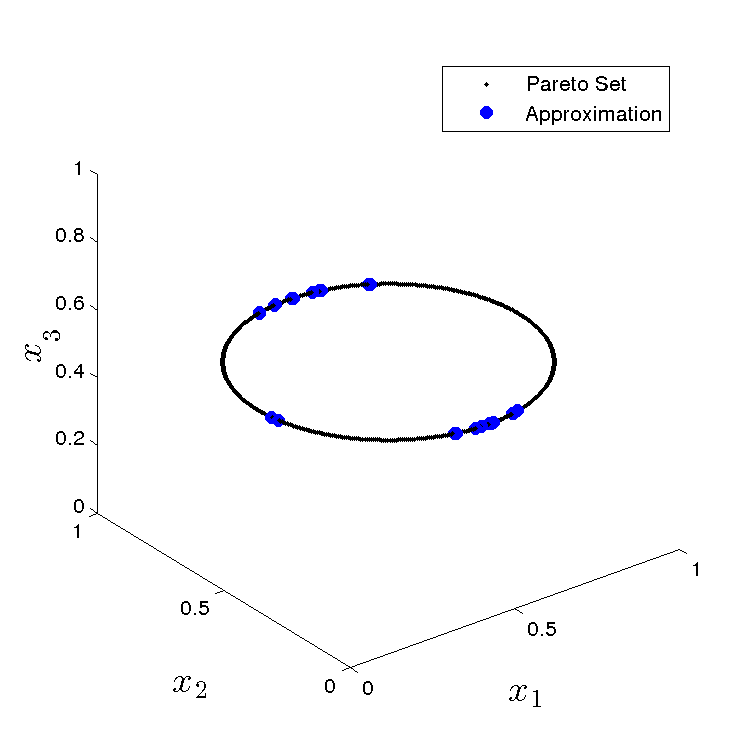

Supplement: Multimedia component 1 [file mmc1.zip › DataInBrief/Images/ps_HV_ANSGAIII_H1_DTLZ1_k3.png]

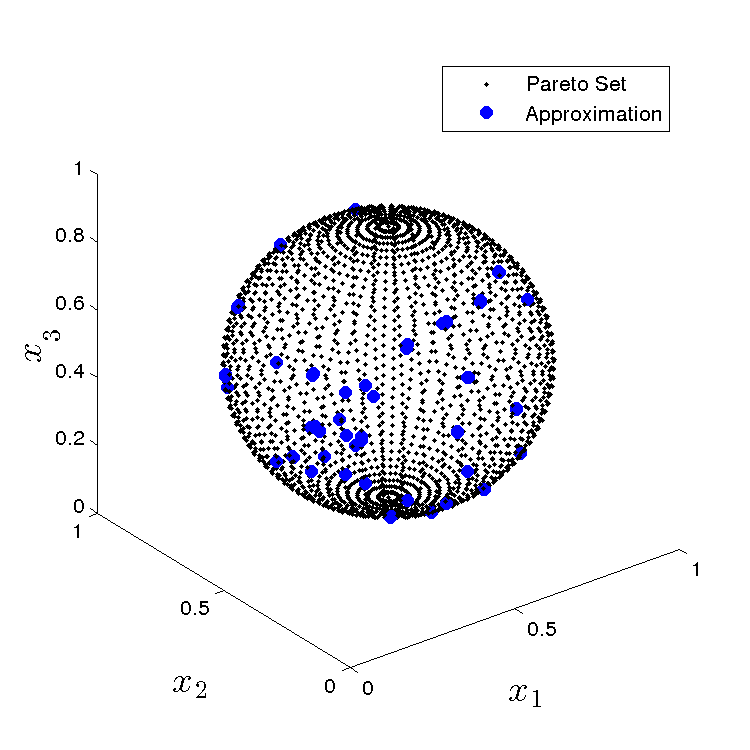

Supplement: Multimedia component 1 [file mmc1.zip › DataInBrief/Images/ps_HV_ANSGAIII_H1_DTLZ2_k4.png]

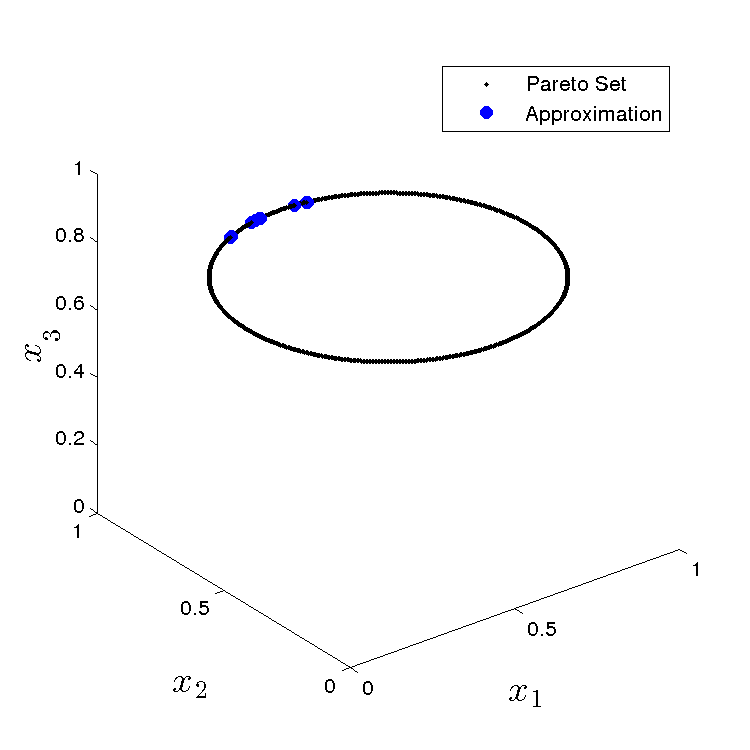

Supplement: Multimedia component 1 [file mmc1.zip › DataInBrief/Images/ps_HV_ANSGAIII_H2_DTLZ2_k4.png]
